# Supplementary material for: Newborn screening timeliness quality improvement initiative: Impact of national recommendations and data repository
Source: PLoS One. 2020 Apr 2;15(4):e0231050. doi: 10.1371/journal.pone.0231050 (PMC7117765; doi:10.1371/journal.pone.0231050)
Supplement: S1 File — (PDF) [file pone.0231050.s001.pdf]

PDSA Personality Test

For each item please rank order the options. Use each number (1, 2, 3, or 4) once for each set of responses.

1. Please rank the following 4 options. Four is the option that you find MOST DESIRABLE while 1 is the option you find LEAST DESIRABLE.
   _____ a. I like to keep things moving quickly.
   _____ b. I like to know the who, what, and when of a project.
   _____ c. I like to analyze data.
   _____ d. I like to check items off my lists and action plans.

2. Please rank the next 4 options. Four is the option that you find MOST LIKE ME while 1 is the option you find LEAST LIKE ME.
   _____ a. I like to generate ideas for next steps.
   _____ b. I am creative.
   _____ c. I like to discuss what I have learned.
   _____ d. I like to complete tasks.

3. Please rank the next 4 options. Four is the option that you find MOST LIKE ME while 1 is the option you find LEAST LIKE ME.
   _____ a. I do not need to be involved in the details of a project.
   _____ b. I often try to keep the big and small picture in mind when approaching a project.
   _____ c. I feel pressured under deadlines.
   _____ d. I do not have to contribute an idea to a project to feel like I have made a contribution.

4. Please rank the next 4 options. Four is the quote that you find MOST LIKE ME while 1 is the quote you find LEAST LIKE ME.
   _____ a. "Where are we headed, what's next?"
   _____ b. "Who's responsible for the task and when will it get done?"
   _____ c. "Who has endorsed these measures, what is included in the data?"
   _____ d. "Just tell me what to do, and I will get it done."

Now add up the scores for:

Your "A" responses: _______

Your "B" responses: _______

Your "C" responses: _______

Your "D" responses: _______

This test was created by Yvonne Kellar-Guenther and is based on Roni Christopher's PDSA Personality sheet. Your PDSA Personality by R. Christopher is licensed under a Creative Commons Attribution-NoDerivs 3.0 Unported License. For more on this test email ykellar-guenther@ciinternational.com or Yvonne.kg@gmail.com

How to Use This Tool

This test was designed to help groups working on a CQI project.

First, have your team each do their own PDSA Personality Assessment. They need to then add up their scores. Here is the key:

A is the Actor
B is the Planner
C is the Studier
D is the Doer

Notes on the scoring – Your score is not good or bad. There is no right or right PDSA personality. The highest score you can get in one area is 16. The lowest score is a 4. The higher score area is your preference or your strength. This is the part of the PDSA cycle you can more easily do and you may want to spend more time in this phase. The lower score area is what you want to do the least. If your scores are evenly spread out you are someone who is aware of all the parts and sees value in the whole PDSA process. You can help your team avoid getting stuck in one place! Also, you should note people can change over time. Just because you are an actor today does not mean that in 6 months you actually score higher as a planner.

Team score – The value in this tool is not in knowing what you are like as an individual, rather it is in knowing what your team is like. Is there a prevalent PDSA personality on your team? If yes, note that you team could get stuck in one phase and may need some prompting or even assistance to complete other pieces (maybe you need a studier).

If your team is made up of all different types of PDSA personality just note that you may have more conflict. Some may want to spend more time planning while others want to skip to action because they "know". This tool can help your team understand why the tension may exist and show that everyone has a role to play in the PDSA process but you have to be supportive when you are not in your "go-to" phase.

This test was created by Yvonne Kellar-Guenther and is based on Roni Christopher's PDSA Personality sheet. Your PDSA Personality by R. Christopher is licensed under a Creative Commons Attribution-NoDerivs 3.0 Unported License. For more on this test email ykellar-guenther@ciinternational.com or Yvonne.kg@gmail.com
