# Supplementary material for: Newborn screening timeliness quality improvement initiative: Impact of national recommendations and data repository
Source: PLoS One. 2020 Apr 2;15(4):e0231050. doi: 10.1371/journal.pone.0231050 (PMC7117765; doi:10.1371/journal.pone.0231050)
Supplement: S2 File — (PDF) [file pone.0231050.s002.pdf]

Timeliness Toolkit for Expanding Newborn Screening Services

A TOOLKIT TO IMPROVE NEWBORN SCREENING TIMELINESS BY EXPANDING LABORATORY AND COURIER OPERATIONS TO HOLIDAYS AND WEEKENDS

This project is supported by the Health Resources and Services Administration (HRSA) of the U.S. Department of Health and Human Services (HHS) under grant number UG8MC28554, Improving Timeliness of Newborn Screening Diagnosis, for $5,400,000. This information or content and conclusions are those of the author and should not be construed as the official position or policy of, nor should any endorsements be inferred by HRSA, HHS or the U.S. Government.

NewSTEPs 360
A Partnership between The Colorado School of Public Health and The Association of Public Health Laboratories™

astho™ 75 years 1942 – 2017

march of dimes®
A FIGHTING CHANCE FOR EVERY BABY™

ACKNOWLEDGEMENTS

NewSTEPs 360, a collaboration between the Colorado School of Public Health (CoSPH) and the Association of Public Health Laboratories (APHL) would like to recognize the following partners and state newborn screening programs that generously supported the development of this toolkit.

Mei W. Baker, MD, FACMG, Wisconsin State Laboratory of Hygiene

Janis Biermann, March of Dimes

Natasha Bonhomme, Baby's First Test of Genetic Alliance

Paige Bussanich, MS, Association of Maternal and Child Health Programs

Melanie Lockhart, March of Dimes

Christi Mackie, Association of State and Territorial Health Officials

Fizza Gulamali-Majid, PhD, Laboratories Administration, Maryland Department of Health

Tonya McCallister, Oklahoma Newborn Screening Lab

Amelia Mulford, Baby's First Test of Genetic Alliance

Richa Ranade, Association of State and Territorial Health Officials

Jacklyn Seisman, Baby's First Test of Genetic Alliance

Scott M. Shone, PhD, RTI International

Kate Taft, MPH, Association of Maternal and Child Health Programs

Kelly Umana, Association of Maternal and Child Health Programs

EXECUTIVE SUMMARY

Each year, 12,000 babies with serious, but treatable conditions grow up healthy because of newborn screening (NBS). NBS is a vital public health program that identifies newborns at risk of developing critical disorders that may not show symptoms at birth, but can cause permanent disability or death if not detected and treated in the first few days of life. It is a complex system that involves families, birthing facilities, laboratories, follow-up programs and healthcare providers. Any delay in the newborn screening process may jeopardize the health and survival of affected babies.

In November 2013, the article Deadly Delays in the *Milwaukee Journal Sentinel* found evidence of serious delays in newborn screening programs across the country due to laboratory closures on weekends and holidays, limited use of courier services to transport samples from hospitals to the NBS lab, and little to no consequences for hospitals that sent late samples.

Over the past 2-4 years, many states have implemented a number of changes in newborn screening practices in order to improve health outcomes for newborns. The most significant of these changes include solutions to address courier transit times and the expansion of NBS laboratory operating hours. In particular, allowing labs to remain open six to seven days per week, greatly increases the lab's ability to receive and process specimens on the weekends. It has been demonstrated that states who have expanded newborn screening lab hours are more likely to achieve the 95% timeliness reporting goal. [1]

The *Timeliness Toolkit for Expanding Newborn Screening Services* was developed by NewSTEPs 360, a collaboration between the Colorado School of Public Health (CoSPH) and the Association of Public Health Laboratories (APHL) to support states and territories to make improvements to their NBS courier and laboratory operations. This toolkit is designed to assist NBS programs, advocates, legal, and public health professionals to shape appropriate policies and communicate the value of adding weekend and holiday operations in order to improve timeliness in newborn screening.

The *Timeliness Toolkit for Expanding Newborn Screening Services* is an interactive online toolkit that is housed on the NewSTEPs website (https://www.newsteps.org/toolkits/timeliness-toolkit-expanding-newborn-screening-services). The toolkit has eight sections with embedded links to supplemental resources, case studies, videos, webinars, and example templates (Table 1). The material contained in this toolkit are designed to be customized based on the needs of the individual state or newborn screening program. This is a "living" toolkit that will be modified as new information and resources becomes available.

[1] For more information, see the 2016 NewSTEPs Timeliness Report submitted to the United States Government Accountability Office.

Table 1: Description and Intended Use of Toolkit Resources and Materials

Section 1: Making the Case for Change: A Tip Sheet for NBS Programs	This tip sheet, developed in partnership with the Association of State and Territorial Health Officials (ASTHO), is intended for NBS program staff to help develop and make the case for changes and improvements to state and/or health department leadership. The tip sheet includes links to ASTHO state stories, Return on Investment (ROI) tool, and a sample fact sheet.
Section 2: Policy Guide	Although all states and territories have laws that require screening, changing those laws and policies governing NBS can vary by state. This Policy Guide, developed in partnership with the March of Dimes, is designed for advocates who may be new to state NBS policy and provides a "how to" on the options and general steps to take towards changing the laws and regulations to meet NBS timeliness goals.
Section 3: Law and Regulations Decision Tree	One of the keys to make changes to your NBS program is to first map out the existing laws and regulations that guide screening in your state. This decision tree, developed in partnership with ASTHO, is designed to guide users to determine where in the legislative or regulatory process they currently are and the next steps needed to make changes in their NBS policy or program.
Section 4: "Cookbook" Policy Guide for NBS Staff	This section, developed with the March of Dimes, provides a guide for NBS staff with important steps to consider when making policy changes, including tips on how to write a policy statement.
Section 5: Communication Materials	Provides a list of communication tools and examples that can be used during the advocacy process. This section includes a PowerPoint template of talking points, and the *Newborn Screening: Achieving Timeliness Issue Brief,* developed by the March of Dimes, providing information, data, and potential policy solutions specific for expanding NBS services.
Section 6: Overview of NBS Weekend and Holiday Operations	This section contains five tables summarizing state specific processes for weekend and holiday operations: 1) specimen delivery; 2) result reporting; 3) laboratory staffing; 4) follow-up staffing; and 5) technical support. For more information about state's implementation processes for expanding NBS operations, the toolkit contains 10 case studies that are highlighted throughout the sections. These narratives provide a roadmap to what and how to implement weekend and holiday operations.
Section 7: Steps and Considerations for NBS Program Staff	Before your state expands NBS services, staff should review this list of considerations to determine logistics, staffing, costs, and approval process considerations at the programmatic level. This list should be used by NBS program staff to make sure that all bases are covered before moving forward with implementing expanded operations with the NBS lab, follow-up and courier.
Section 8: Newborn Screening Timeliness Simulator	The Newborn Screening Timeliness Simulator allows NBS programs and hospitals to evaluate the time it takes dried blood spot specimens to reach the NBS laboratory from the time of birth. Users can replicate the simulation used in Michigan birthing hospitals [1] or use their own data (aggregate or hospital level) and experiment by changing variables related to collection time, courier pick-up times, and laboratory hours to see how these changes affect timeliness. NBS programs can compare findings to determine where in the collection and delivery processes causes delays and identify strategies to make improvements for timely laboratory receipt.

1. Cochran AL, Tarini BA, Kleyn M, Zayas-Caban G. Newborn Screening Collection and Delivery Processes in Michigan Birthing Hospitals: Strategies to Improve Timeliness. Matern Child Health J. 2018;22(10):1436-43. Epub 2018/04/05. doi: 10.1007/s10995-018-2524-z. PubMed PMID: 29616441.
