# Supplementary material for: Newborn screening timeliness quality improvement initiative: Impact of national recommendations and data repository
Source: PLoS One. 2020 Apr 2;15(4):e0231050. doi: 10.1371/journal.pone.0231050 (PMC7117765; doi:10.1371/journal.pone.0231050)
Supplement: S3 File — (PDF) [file pone.0231050.s003.pdf]

BUILDING BLOCKS

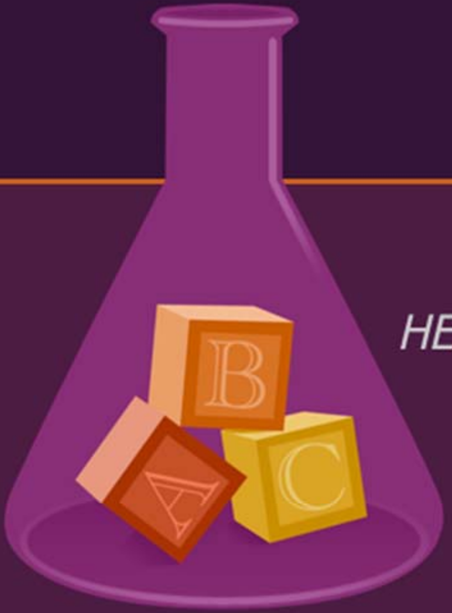

NEWBORN SCREENING

HEALTH IT IMPLEMENTATION

GUIDE AND TOOLKIT

NewSTEPs
A Program of the Association of Public Health Laboratories™

DCLS

J MICHAEL CONSULTING

This project is supported by the Health Resources and Services Administration (HRSA) under grant # UG8MC28554 Improving Timeliness of Newborn Screening Diagnosis $1,800,000. Its contents are solely the responsibility of the authors and should not be construed as the official position or policy of, nor should any endorsements be inferred by HRSA, HHS, or the US Government.

This publication was developed by J Michael Consulting, LLC, in collaboration with the Virginia Division of Consolidated Laboratory Services (DCLS) and the Newborn Screening Technical assistance and Evaluation Program (NewSTEPs), a partnership between the Association of Public Health Laboratories (APHL) and the Colorado School of Public Health (ColoradoSPH). Its contents are solely the responsibility of the authors and do not necessarily represent the official views of APHL or ColoradoSPH.

Version	Release Date
1	8/31/2017
2	5/21/2018

Table of Contents

Table of Contents 3

Table of Figures 5

Introduction 6

Organization 7

Section I: Getting Ready 9

Section I Introduction 9

1. Initiate and Plan the Project 10

Summary 10

Tasks 10

1.1 Initiate Project 10

A. Identifying a Lead 11

B. Business Case 11

C. Project Charter 11

D. Data Flow Diagram 12

E. Readiness Assessment 12

F. Cost Estimates 13

G. Authorizing the Project 15

1.2 Develop a Project Management Plan 15

1.3 Identify Project Team and Stakeholders 15

2. Define the Message 19

Summary 19

Tasks 19

2.1 Understand the Standard 19

2.2 Constrain the Standard 20

2.3 Perform the Gap Analysis 20

2.4 Create the Implementation Profile 21

2.5 Define Conformance 21

2.6 Identify Vocabulary 22

3. Identify a Technical Solution 25

Summary 25

Tasks 25

3.1 Gather Technical Documentation 25

3.2 Design Technical Solution 26

A. Existing Components of the Technical Architecture 27

B. Standard Terminology Mapping, Translation and Maintenance 28

C. Data Import / Extract from Source System(s) 28

D. Transformation of Source System Data Elements 28

E. Secure Transport / Receipt of Messages 28

F. Data Privacy 28

4. Prepare to Send and Receive Messages 29

Summary 29

Tasks 29

4.1 Analyze Laboratory Workflow 29

4.2 Update LIMS 32

4.3 Configure Integration Engine 32

5. Set Up Validation Method 35

Summary 35

Tasks 35

5.1 Test Planning and Performance 35

A. Unit and Functional Testing 35

B. System and Integration Testing 36

C. Performance and Stress Testing 37
D. Resources and Expertise 37
Section I Conclusion 37
Section II: Managing Relationships with Hospitals 39
6. Partner with Hospitals 39
Summary 39
Tasks 40
6.1 Identify Hospitals 40
A. Who and How Many 40
B. What to Tell Hospitals 41
C. Assess the Hospital's Readiness 42
6.2 Communicate with Partners 42
6.3 Mitigate Delays 43
A. Project Authorization 43
B. Partnership Documents 44
C. Hospital IT 45
Section II Conclusion 45
Section III: Implementing a Data Exchange with a Messaging Partner 47
Section III Introduction 47
7. Establish Connectivity 47
Summary 47
7.1 Develop Messaging Policy Document 48
7.2 Review and Sign Off on Security Policy 48
7.3 Establish and Test Connection 48
8. Complete Message Validation 49
Summary 49
Tasks 49
8.1 Perform Test Cases 49
A. Consume Test Orders 50
B. Send Test Results from LIMS 50
8.2 Incorporate Changes 50
8.3 Re-perform Test Messages 51
9. Preparation and Go-Live 53
Summary 53
Tasks 53
9.1 Train Laboratory Staff in New LIMS Functions 53
9.2 Cut Over to Production 54
A. Schedule Go-Live Event 54
B. Develop Transition Plan 54
9.3 Close Out 54
A. Turn Off Paper Reporting 54
B. Retire Legacy Systems 55
C. Celebrate 55
D. Schedule Post Go-Live 55
E. Post Go-Live Communication 55
Section III Conclusion 55
Section IV: Operations and Maintenance 57
10. Transition to Operations and Maintenance 57
Summary 57
Tasks 57
10.1 Communication Post Go-Live 57
10.2 Monitor Data Exchange 58
10.3 Evaluate and Improve Process 58
10.4 Perform Change Control 59

10.5 Fulfill Ongoing Training Needs 60
Section IV Conclusion 61
Building Blocks Next Steps 61
Acknowledgements 61
Abbreviations and Terms 63
Appendix A: Tools Reference Guide 1
Introduction 1
Tools Appendix Index 2
Appendix B: SME Matrix 1
Appendix C: Case Studies 1

Table of Figures

Figure 1: Guide to the Guide 8
Figure 2: Creating a Fully-Constrained Implementation Profile 23
Figure 3: Sample Architectural Diagram 28
Figure 4: Three Levels of Workflow Analysis 32
Figure 5: Thoroughly Test Mock Orders 50
Figure 6: Change Control Workflows 60
Figure 7: Detailed Description of Subject Matter Experts (SME) B-1
Figure 8: Subject Matter Experts (SME) by Chapter B-2

Introduction

The NewSTEPs 360 project, funded by the Health Resources and Services Administration (HRSA), works with newborn screening (NBS) programs to improve the timeliness and accuracy of NBS from birth to results reporting. Because the immediacy of results could mean life or death for a newborn, the NewSTEPs mission includes activities to implement health information technology solutions and electronic messaging. The use of electronic messaging for NBS speeds the results process, thus providing the most efficient, accurate and earliest care to our youngest citizens.

Several NBS programs have implemented, or are in the process of implementing, NBS messaging using program-specific methodologies with varying levels of success. To address the different approaches and inconsistent results of NBS messaging projects, NewSTEPs 360 partnered with the Virginia Division of Consolidated Laboratory Services (DCLS) and J Michael Consulting (JMC) to assemble a resource guide that offers the NBS community practical instructions and best practices for implementing a NBS electronic data exchange.

Building Blocks: Newborn Screening Health IT Implementation Guide and Toolkit is intended to fill a critical void in the world of newborn screening. The purpose of the Guide is to provide practical information to project teams that are instituting electronic messaging for NBS programs. The Guide uniquely addresses the diverse audiences related to initiating and sustaining this project and clearly outlines the steps needed to stand up electronic messaging with partners from start to finish. The detailed content of the Guide speaks to all those involved in this kind of project—laboratory professionals, project managers, hospital administration, subject matter professionals, nurse managers, and project stakeholders, among others.

The Guide is written primarily from the perspective of a state public health laboratory implementing electronic test orders and results (ETOR) with at least one hospital. In certain cases, it may be the NBS program that is managing the implementation. Nevertheless, throughout this document, we refer to the "laboratory" as the responsible entity for stylistic simplicity and because the laboratory will be the entity most affected by the new processes. In our hypothetical implementation, the laboratory has opted to utilize the Health Level 7 (HL7) 2.5.1 standard to accomplish this ETOR. It is assumed that the laboratory will design messages based on the HL7 Laboratory Results Interface (LRI) and Laboratory Orders Interface (LOI) Implementation Guides developed by the Standards and Interoperability (S&I) Framework. HL7 is generally considered to be the common standard for electronic public health messaging, but the laboratory may choose an alternative form of data exchange, such as a web portal. While HL7 messaging is the focus of this Guide, the majority of the advice offered applies to any type of data exchange implementation.

To provide targeted help for a variety of disparate professionals, the Guide is designed to be modular in use. This arrangement allows project teams or individual contributors to go directly to the portions of the guide that pertain to their specific needs, regardless of their focus. In other words, users can approach the Guide in an à la carte fashion. For example, a project manager may not need to take a deep dive into the nuts and bolts of message orders, while a subject matter expert (SME) may appreciate the more granular details related to HL7 messaging. Readers can use the SME Matrix described below to navigate to the content that is most relevant to them.

Organization

Arranged in four ***Sections***, the Guide is broken down by ***Chapters*** that trace the process and outline the requirements needed to stand up electronic messaging with partners successfully. Within each chapter lie specific ***Tasks*** to be completed. Each chapter opens with a table that provides an at-a-glance look at the dependencies, resources, and timeline and informs users about specific tools, key outcomes, and case studies related to the tasks included. Again, users may choose to skip those sections, chapters, or tasks that do not apply to their roles, programs, or timelines.

Section I describes the project initiation and planning that the laboratory will need to accomplish before working with a hospital partner to implement NBS messaging. Section II lays out strategies and considerations for managing the laboratory's relationship with hospitals and for helping hospitals work the project through their internal approval process. Section III summarizes the steps that laboratories and hospitals will need to accomplish in order to set up the connection and test the data exchange from both a structural and a content perspective. Finally, Section IV walks the reader through the continued operations and maintenance that the laboratory and hospital will need to set in place over the long term.

The ***Appendices*** that follow enable the reader to use the Guide more dynamically and to access the tools referenced throughout the Guide. Appendix A: Tools Reference Guide lists out and describes the tools mentioned in the body of the Guide. For each tool, the appendix indicates where to go for more information and examples. Note that the Building Blocks team developed several of these tools specifically for the implementation of HL7 NBS messages. Appendix A provides links to access these tools online. Appendix B: SME Matrix sorts the content of the Guide by the SMEs who will need to be involved in each step of the process. In other words, SMEs can use this appendix to quickly identify the chapters that discuss the activities that they will perform on the project. The project manager can also use this appendix to plan when and where to pull in different personnel resources over the course of the implementation. Many members of the NBS community contribute to the accounts presented in Appendix C: Case Studies. These studies are drawn from the actual experiences of laboratories as they work towards NBS messaging. To the extent possible, these case studies have been cross-referenced with topics covered in the Guide.

Note that the Building Blocks Guide also includes a ***List of Abbreviations*** and a ***Glossary*** to provide clarity on specific terms used in the text. While many of these terms are identified in the text, this resource provides a comprehensive reference for the industry-specific language used in the Guide.

The following graphic provides a visual overview of this breakdown to help readers navigate the Guide more easily.

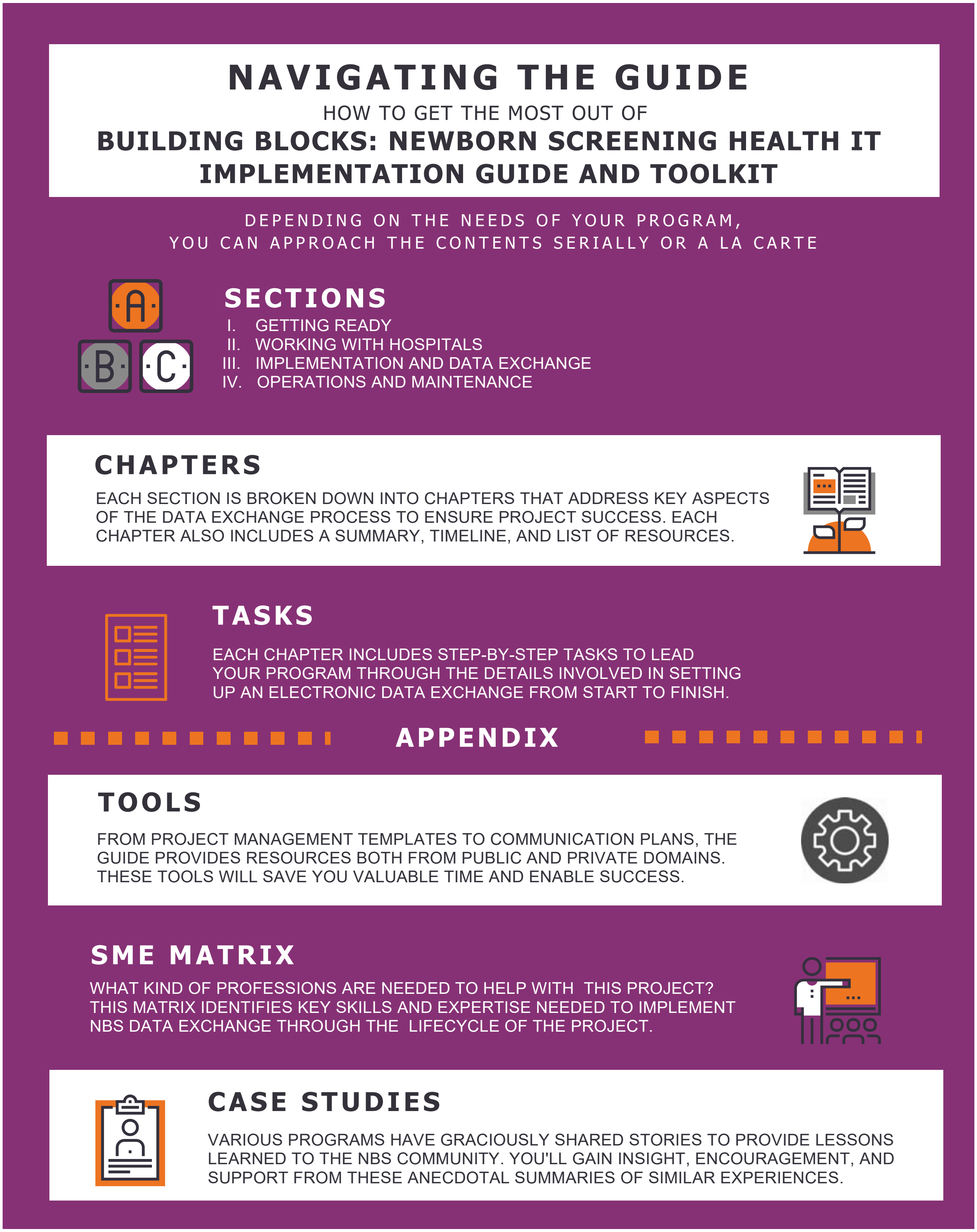

Figure 1: Guide to the Guide

Section I: Getting Ready

Section I Introduction

The activities described in Section I help guide laboratories in initiating successful implementation of NBS electronic messaging. The first step in preparing for any health IT implementation is to assemble internal stakeholders, agree on the scope and objectives of the project, and assess the laboratory's readiness to take on this project. The laboratory must create documentation and project artifacts to help describe the project to the decision makers who can authorize the project. A significant effort in this planning stage may focus on cost and resource estimates and identifying funding.

Next, the laboratory must evaluate and prepare its internal workflow, messaging capabilities, vocabulary, and systems to accommodate electronic messaging. At this stage, the laboratory should select a messaging guide and define the message content and requirements clearly. The laboratory should also diagram the technical solution that it will use to enable the laboratory systems to receive and process test orders and generate and send results. In concert with these activities, the laboratory is likely building relationships with hospital partners; these efforts are summarized in Section II. Once the steps described in Section I have been accomplished, the laboratory will be ready to begin implementing NBS messaging with hospital partners.

1. Initiate and Plan the Project

Summary

For a large-scale data exchange implementation project to be successful, it is essential that the NBS laboratory invest time at the beginning to create a set of project management documents that define the scope of the project and all parties involved, lay out the anticipated timeline, and identify milestones and factors to measure progress and success. A project management plan will establish a solid foundation for the project, present the consensus strategies for managing all aspects of the project and help the team guide the project through its entire lifecycle.

Dependencies	None	
Personnel Resources	Lab Leadership<br>Project Manager<br>Business Analyst	Lab Program SME<br>Technical SME<br>Other SMEs consulted as necessary, for example hospital SME
Timeline	The timeline to complete the planning activities and formally initiate the project will depend on the laboratory's state of readiness regarding funding, technical capabilities, and resource availability. This initial stage may span a few months or more than a year.	
Tools	Business Case<br>Communication Plan<br>Example Budget<br>Message Flow Diagram<br>PHII Communications Toolkit	Project Charter<br>Project Management Plan<br>Project Schedule<br>Risk Management Plan<br>Stakeholder Matrix
Key Outcomes	The project team will have a portfolio of fully-developed project artifacts, such as a business case, project charter, and/or message flow diagram, and a project management plan and associated documentation. The team will have obtained authorization to formally initiate and fund the project and will be ready to begin work.	
Case Studies	below<br>below	

Tasks

1.1 Initiate Project

The Project Management Institute's authoritative Project Management Body of Knowledge (PMBOK) identifies project initiation as the first phase in a project lifecycle.[1] The team uses this initial phase to draft a tentative plan, articulate the overall objectives of the project, and obtain authorization from key decision makers. The duration and level of effort in this first activity will vary by laboratory. It may be a single meeting or require several months of discussions. In general, it is advised that the laboratory anticipate the initial project planning process to take at least two to three months.

[1] *Project Management Book of Knowledge.* 2013. Fifth Edition. Project Management Institute.

A. Identifying a Lead

As soon as the laboratory has determined that NBS messaging is a priority, the laboratory director or division chief should identify a project manager to take the lead on the planning. The effort will move forward more quickly with a dedicated individual to coordinate stakeholders and advocate on the project's behalf. This individual, whether a member of laboratory leadership, a laboratorian, an IT resource, or other SME, should have strong communication and organizational skills and possess a basic understanding of both laboratory and IT processes. This individual should have the authority to pull the necessary internal resources together and should be prepared to see the project through the initiation and planning stages.

In the initiation phase, the project manager will facilitate meetings of various stakeholders and help the laboratory navigate the early decision-making process. As part of this process, the project manager will develop artifacts that describe the project at a high level. This preliminary documentation may include a business case, a project charter, a message flow diagram or other artifacts. These artifacts should be well documented and easily accessible to current and future laboratory staff. As project teams change, strong documentation will ease transitions between staff.

B. Business Case

A business case summarizes the justification for starting a new project. It defines the problem that the project is attempting to address and explains the proposed solution. It often provides both quantitative (e.g., decreased cost) and qualitative (e.g., improved customer experience) benefits of the project. For example, in the case of NBS electronic messaging, the problem being addressed may be a delay in testing NBS specimens due to manual demographic data entry. The business case in this instance should include information about the percentage of births each hospital represents within the state and basic information about the electronic health records (EHRs) that they use.

The business case should also include a cost benefit analysis. This analysis may describe the potential benefits to hospitals implementing the NBS data exchange, in addition to the benefits to the NBS program, both in terms of money saved long-term, the number of potential newborns saved, improvements to work processes, and time saved in testing NBS specimens. The value received for all involved parties by completing the project should be readily understood by the decision makers and backed up with documentation, if possible. While this analysis may not be comprehensive, it is important to document the estimated monetary and non-monetary costs of implementing NBS electronic messaging, as well as the cost of doing nothing, such as risks associated with delayed testing and/or reporting. Laboratorians and technical SMEs can provide input on the costs associated with assessing and changing laboratory workflows, purchasing and implementing new software, modifying the laboratory's system architecture, and long-term maintenance and operation costs.

C. Project Charter

In many ways, the project charter builds on the business case. It itemizes the objectives of the project and the general roles and responsibilities of each party. It begins to hone in on the project scope and timeline. If the laboratory has any target milestone dates, these should be documented. For example, does the laboratory intend to have one hospital in production by the end of the fiscal year, or some percentage of all NBS orders submitted electronically within two years? Keep in mind that outside factors may affect this timeline (e.g., system upgrades, funding deadlines).

For NBS messaging, it is also important that the project charter identifies the specific data-messaging standard (e.g., HL7), the specific implementation guide for that messaging data standard, and the terminology data standard to be implemented (e.g., Local Observation Identifiers Names and Codes [LOINC]). The charter should also identify the approach that the laboratory will use to constrain the implementation guide for use by that laboratory in that specific use case (see Define the Message for more details on this process). The project manager can use the project charter to present the details of this initiative to laboratory leadership and to other stakeholders.

D. Data Flow Diagram

It is highly recommended that the laboratory also draft a tentative data flow diagram as part of this exercise. The project manager should draw on technical SMEs to help develop this diagram and explain the laboratory's current data exchange capabilities. It may be that the NBS program can leverage existing solutions to facilitate NBS messaging. The data flow diagram will begin to flesh out the details of the transport mechanism and the specific laboratory information systems that will be used to send and receive the NBS messages. See Identify a Technical Solution for an example diagram and more information on how to identify and design a technical solution for NBS messaging. In addition, the laboratory will need to confirm whether messaging partners will exchange data directly or if they will use a health information exchange (HIE) to route the message. The laboratory can review this message flow diagram with IT leadership to identify potential system modifications or integrations, security concerns, and other issues.

E. Readiness Assessment

Before beginning the implementation in earnest, it is advised that laboratories take time to assess the readiness of their systems and the availability of resources. This task should be completed in parallel with the project charter since it will reveal key tasks needed for a successful project. From a technical perspective, the laboratory's infrastructure (i.e., the hardware, software, network resources, and related systems) should have the minimum requirements to generate, validate, transport, and receive HL7 messages. The laboratory should also have a test environment that mimics the workflows of the production environment and allows the LIMS administrator to evaluate intended changes before deploying them to the live system. The relationship of the systems in the test environment should mirror those of the production environment. The laboratory must also be able to support whatever mechanism is chosen to transport the data between the laboratory and the hospitals.

> "Programs serve different public health needs, but we learned from talking with other programs about pitfalls and successes in informatics. Most tools and lessons learned can be adapted to all public health electronic reporting projects. This is a lesson we can learn over and over again."
>
> Newborn Screening (NBS) Program at the Minnesota Department of Health (MDH); Case Study #1

In addition to these technical considerations, the project manager will need to review the IT team's calendar to coordinate the implementation of NBS messaging with any planned system upgrade or other major IT projects. The project manager should also engage the laboratory's legal and IT security SMEs during this assessment to inventory any significant considerations that will need to be resolved before the project can be authorized or that will need to be addressed at a later stage.

In many ways, the Guide serves as a checklist that laboratories can use to assess their readiness to move forward with the implementation of NBS messaging. Laboratories can use it to evaluate steps already taken as well as those yet to be completed. While not every task will need to be completed in sequential order, most steps will need to be accounted for at some point during the implementation process. Therefore, the laboratory should be prepared to meet the technical requirements and message specifications necessary to establish the data exchange.

In addition to the technical development, the laboratory can expect substantial changes to accessioning, processing orders, and verifying and sending results. See Prepare to Send and Receive Messages) for a summary of the questions that the laboratory should consider when assessing current workflows. The laboratory staff will need to plan and prepare for these changes.

The project team should carefully review this entire Guide to determine the laboratory's readiness to implement the test order and result messages. The laboratory is also encouraged to review and complete the APHL Informatics Self-Assessment Toolkit to evaluate the laboratory's overall informatics capabilities. The technical team may need to enhance certain areas of the laboratory's technical infrastructure before the implementation begins.

F. Cost Estimates

A serious concern for laboratories is how to produce a realistic budget estimate for a large-scale messaging project. Unfortunately, given the unique circumstances of each laboratory, it is impractical to estimate average costs. Not all costs will apply to all laboratories. Moreover, the level of effort required to complete the various tasks will depend on the laboratory's systems, contracts, staff expertise, and other considerations.

Rather than presenting overall project estimates, therefore, this Guide provides a list of factors that the laboratory should review in budgeting for this project. In addition, Virginia DCLS has provided an Example Budget that laboratories can use as a reference and model.

<u>Internal Staff Resources</u>

The Appendix B: SME Matrix in Appendix B summarizes the types of internal personnel resources that each step of the project will require. The laboratory can use this matrix to estimate staff assignments based on the scope of each task.

<u>Centralized Versus In-House IT Resources</u>

Some states have set up a shared services model to deliver IT support to all areas within the department of health, or even across multiple agencies. The laboratory may need to develop a plan for funding this IT support. Similarly, if the laboratory uses the State Health Information Exchange (HIE) as its point of connection for NBS messaging, it may be necessary to include HIE setup and maintenance costs in the project estimate.

<u>Vendors</u>

While some laboratories will be able to rely predominantly on in-house resources, others will need to bring in vendors to update their systems. Most laboratories use a LIMS to record and track their NBS testing activities. This project will likely require updates to the LIMS in the form of modifications or additions of certain data elements. Many laboratories use specialized software to translate and

transform the data coming out of the LIMS into an HL7 message that can be sent to a recipient. This message broker or integration engine also performs the same function to allow the LIMS to consume an incoming message. The laboratory may have a contractual arrangement with the LIMS or integration engine vendor and may need to engage the vendor's services to make the required updates. The laboratory will need to factor the costs and schedule for these services into the overall project plan and budget.

Some vendors offer an application to hospitals that assists in collecting and configuring the EHR data needed to submit electronic orders to the laboratory. These applications interface with the EHR to collect auxiliary NBS data and, in many cases, create and send the electronic NBS message to the laboratory. Many laboratories and hospitals have found this service beneficial for several reasons. First, it takes the burden off the hospital IT team and may speed up implementation. Second, the vendor has the primary responsibility for engaging the messaging partners and providing technical assistance and training for both sides. Third, this approach leverages work that has already been done to interface with other laboratory and hospital systems. Nevertheless, relying on third-party software limits how much the laboratory can tailor the process or message to match existing workflows. Both the hospital and the laboratory are also then dependent on the vendor not only for the implementation but also for continued maintenance and operations. The laboratory should also be cautious about assuming that all hospital systems will agree to use third-party vendors for NBS messaging. The laboratory risks having to implement and maintain data exchanges through the vendor as well as with hospitals that opt out of using the software solution. Finally, this software will likely require licensing, a cost which will have to be negotiated between the laboratory, the vendor and hospital partners.

<u>Sustainability</u>

Keep in mind that data exchanges require continued operations and maintenance once they are in production. NBS messages may arrive at the hospital at any time, and the laboratory may need to arrange for a help desk or other on-call services. In addition, the laboratory staff should monitor and review and verify both the data feed and the contents of the message as well as plan and roll out updates. The laboratory will need to assess and account for the continuing expenses associated with NBS messaging to make the data exchange sustainable.

Maintaining project documentation is an important as laboratory staff turnover. By keeping project documentation up-to-date and readily accessible the project manager will streamline the onboarding and transition of staff when it is needed. The project team should consider using a document storage and sharing software to manage these documents (ex. SharePoint)

<u>Funding</u>

Some laboratories are committed to replacing legacy systems with electronic NBS messaging and begin work on the project without a clear cost estimate. Others insist on obtaining a definitive source of funding for the project in advance.

Many laboratories use the business case to develop a grant application. Laboratories can strengthen an application by identifying and partnering with a particular pilot hospital partner to demonstrate that they have laid the groundwork for a successful project. Keep in mind that identifying and procuring a

funding stream, such as a grant, may take considerable time. The project may be put on hold for several months or longer while the laboratory waits for funding to come through.

The laboratory may determine that the cost of NBS messaging can be rolled into testing fees. The funding approach will depend on the laboratory's administration and business processes. Regardless, it is advisable that laboratory leadership review the costs associated with NBS messaging and agree on a strategy, both for the implementation and long-term maintenance.

G. Authorizing the Project

By the end of this first set of tasks, laboratory leadership should have formally authorized the project as it is described in the business case or project charter and identified a funding stream. Each laboratory will have its own policies and protocol around the exact mechanism for project authorization. Once the project is authorized, leadership should assign a project manager who will guide the implementation through to completion.

1.2 Develop a Project Management Plan

A well-defined project management plan (PMP) is a set of documents that addresses a host of considerations for effectively managing a project. It lays out objectives and milestones as well as resources and cost, so everyone knows what to expect. It documents the approach for tracking and managing all aspects of the project, including schedules, changes, resources, risks, communications, etc. In many instances, the PMP will formally document many of the decisions that the laboratory has already made and summarized in the project charter, the stakeholder matrix, and other artifacts. The PMP should be considered a living document (or set of documents), as the project team will continually reference and update the plan throughout the life of the project. The PMP will serve as a strategic guide to help the team respond to changing circumstances over the course of the project.

The Appendix A: Tools Reference Guide in Appendix A provides examples of project management plans. Note, however, that many states and agencies require staffs to use specific templates to document project planning and management activities. Moreover, to secure funding and authorization, the project manager must ensure that the project documentation includes all the information and details as mandated by the jurisdiction. The project manager should carefully review the requirements within the jurisdiction as the team begins to prepare the project management plan and associated documentation.

1.3 Identify Project Team and Stakeholders

A complex data exchange implementation project will involve many diverse stakeholders, including internal laboratory staff and external partners. Therefore, it is highly recommended that the project manager develops a stakeholder matrix early on to list out the stakeholders, their impact and influence over the project and their specific needs or interests. The project team will repeatedly refer to this matrix during the planning stages as well as throughout the project lifecycle.

Internal Stakeholders

The project manager should define the roles and personnel resources needed at different stages of the project, such as IT leadership (Chief Information Officer), a data standards SME, a LIMS administrator, other relevant technology system administrators, the NBS program manager, a network engineer and a

technical architect. The project manager will likely need to engage stakeholders beyond the laboratory as well, such as government officials, legal representatives, IT leaders and other leadership within the agency or the state HIE.

The Appendix B: SME Matrix in Appendix B provides a detailed list of the types of resources that may be required to complete the various activities associated with an implementation project. The project manager can use this matrix to identify individuals who will perform each required role on the project. In general, NBS program SMEs are primarily involved early in the process to reconcile HL7 fields with LIMS data while the laboratory's IT implementer is involved later to create and validate the HL7 message. The level of participation for each stakeholder will vary depending on the division of work within the laboratory and the subject matter expertise required for the activities. While certain stakeholders may serve in supplemental capacities, joining the project as their expertise is needed, the project would benefit from having a small, dedicated team made up of laboratorians and IT personnel who are assigned to the project for its entire lifecycle.

Engaging with these internal stakeholders early will provide the project manager with a vision of the feasibility of the project. This task may be accomplished most effectively through one-on-one conversations with laboratory and IT staff. The project manager should indicate the proposed project timeline and encourage individuals to discuss competing priorities.

> The NBS HL7 project took a long time to gain momentum. At the beginning of the HL7 NBS project, we underestimated the amount of work needed to complete such an endeavor. We later realized that we needed a person knowledgeable in NBS disorders who *also* had a working knowledge of the laboratory information management system (LIMS) to perform the data mapping.
>
> Michigan Newborn Screening Program; Case Study #7

<u>External Stakeholders</u>

Depending on the contractual arrangements that the laboratory holds with vendors, the project manager may need to engage external stakeholders, in other words, partners outside of the laboratory or health department system, such as LIMS vendors, third-party software vendors or others.

The project manager should meet early on with any vendors to discuss the project and estimate costs and the timeline for modifications. The vendor may have insight into similar projects at other client sites and may be able to provide valuable reusable components for the project. Note that some stakeholders may require a formal contract to secure their support.

<u>Hospitals</u>

While in the planning stages, laboratories should carefully read Section II: Managing Relationships with Hospitals of this Guide which describes strategies for "Managing Relationships with Hospitals." The project manager will need to engage hospitals early and often during the planning stages. In addition, the project team will likely need to produce some of the artifacts described in Section II to help explain the project to hospitals and obtain buy-in from them. Many laboratories have cited lack of hospital engagement as an obstacle to project success. It is strongly encouraged that hospitals be fully engaged and committed to the project before the work begins in earnest. The team should be aware that as they engage with multiple hospitals, each may have different requirements for the exchange process – it will not be a "one size fits all" solution.

Communication

The project manager should be able to leverage the project charter to explain to stakeholders the project's objectives and the types of resources that may be needed and to secure their commitment to the project. Keep in mind, though, that it will likely be necessary to communicate the objectives of the implementation project in non-technical language to stakeholders who lack a general knowledge of laboratory informatics. It is often difficult to explain informatics design and requirements to administrators, financial and legal experts, or even to laboratorians. The Public Health Informatics Institute (PHII) recently released the PHII Communications Toolkit, which provides recommendations for how to present and discuss informatics concepts to non-technical audiences. The project manager may consider incorporating some of these communication strategies in the discussions with stakeholders.

2. Define the Message

Summary

Standards like HL7 make it possible for the laboratory to build to a common specification, ensuring that we are consistent with how data is reported for a specific use case like newborn screening. It provides a framework or blueprint from which to work. However, base standards lack the specificity to be used as-is. A successful implementation requires that these standards be constrained to meet the requirements of the implementer or newborn screening program. This specification, or implementation profile, is utilized by both sender and receiver and should be created with both partners in mind.

Creating an implementation profile requires a familiarity with the base HL7 standard, the mechanisms for its constraint, and an understanding of program requirements. This section will cover some of the key tasks and questions pertinent to developing a messaging guide. Further information on fundamental HL7 concepts may be found on the HL7 website. Vendors may be able to assist with these tasks or complete them in their entirety, and the laboratory may want to consider contracting with a vendor if needed.

Dependencies	Initiate Project	
Personnel Resources	Business Analysts<br>HL7 SME<br>Lab Program SME	LIMS Administrator<br>Project Manager<br>Vocab SME
Timeline	The time and resources necessary to create an implementation guide can vary. The implementer's degree of familiarity with the HL7 standard and knowledge of the NBS program's specific data requirements and business processes may affect the timeline. Previous experience with the process of profiling and any existing documentation can significantly reduce the time needed to complete this task.	
Tools	Baby Steps Toward Defining the Message<br>Example Message<br>Implementation Guides (LOI and LRI)<br>Implementation Profile<br>HL7 Viewer	Implementation Workbook (LOI and LRI)<br>Message Flow Diagram<br>Newborn Screening Coding and Terminology<br>Value Set Companion Guide<br>Messaging Test Cases
Key Outcomes	By the end of this task, the laboratory will have created a fully constrained implementation profile and mapped data elements to their appropriate location in the message.	
Case Studies	below<br>below	

Tasks

2.1 Understand the Standard

Clearly communicating expectations for how senders and receivers will process NBS orders and results is essential to successful data exchange. Working toward an agreement on a singular way of doing things reduces the time and resources needed to maintain unique interfaces with each messaging partner. An understanding of how these standards work is necessary to both leverage the existing framework and to

meet the laboratory's needs. However, the laboratory may have to be flexible with their partners and weigh the risks of accommodating one-off solutions versus the benefit of on-boarding partners.

While HL7 publishes multiple standards for data exchange, the public health community has most commonly adopted the HL7 2.5.1 version. Because HL7 is designed to be broadly applicable to many use cases, the HL7 2.5.1 standard is not defined at a granular enough level to support a specific use case like newborn screening. Further definition or constraint of the base is necessary to remove ambiguity and define exactly what data should be included in an HL7 message.

2.2 Constrain the Standard

To have a viable data exchange process, the data contained in the message must be appropriate to meet the needs of the laboratory and its partner. This means that the all data elements must be clearly defined as far as optionality and the type of data transmitted. Openness in HL7 may be described by the inclusion of optional data elements or those that are not entirely defined, which can be difficult to implement for a specific use case. The process of constraining or reducing the openness of a standard is called "profiling," and it is necessary to tailor the HL7 framework for a specific use.

Several initiatives have carried out work on a NBS profile. Recent efforts have incorporated the newborn dried bloodspot screening (NDBS) use case into the HL7 Laboratory Results Interface (LRI) and Laboratory Orders Interface (LOI) Implementation Guides developed by the Standards and Interoperability (S&I) Framework. This work built on the Public Health Informatics Institute (PHII) implementation guide for NBDS laboratory results, most recently updated in 2011. LRI and LOI are based off the HL7 2.5.1 standard and define requirements for electronic ordering and resulting of laboratory tests — specifically the implementation of laboratory orders and results interfaces in healthcare facilities. The creation of the NDBS profile component further constrains the standard for sending electronic laboratory orders and results for NDBS testing.

LOI and LRI are structured into groups of requirements called "profile components." Implementers may define their profile to a certain extent by their selection of these components. This first step will set programs up to further define or tailor the standard to their state's NBS program requirements. For more information on selecting these components see Baby Steps , a companion resource that is included in the Tool Reference Guide in Appendix A.

2.3 Perform the Gap Analysis

Selection of the appropriate laboratory profile components results in a constrained profile. However, the requirements of this profile probably will not yet meet the specific needs of the NBS program. For orders, the laboratory may collect only a subset of the data elements supported by the HL7 Orders Profile. Similarly, it may report only some of the data elements associated with the results. Further definition or constraint of this profile is needed to create an implementation profile that is specific to the laboratory's NBS program. To identify which data elements will need to be defined further, the team must determine which segments and fields the laboratory will use to send and receive NBS orders and results. This will require a gap analysis.

At a minimum:

- For laboratory orders, the gap analysis will likely include a comparison between the collection card and the LOI profile.

- For laboratory results, the gap analysis will compare results reports (usually paper) and the LRI profile.

The gap analysis should result in documentation that clearly captures which data elements will be collected for both orders and results and where there are discrepancies with the LOI or LRI profiles to which your messages will adhere. For more information on performing a gap analysis see Baby Steps .

The laboratory may share this documentation with partner hospitals at this point to assess whether 1) the hospital can realistically provide the requested data for the order; and 2) the hospital has the capability of receiving and consuming elements conveyed in the result message. Feedback from the hospitals may indicate that the laboratory will need to modify the guide. The laboratory should consider the hospitals partners in this process and work together to determine a fully implementable profile.

2.4 Create the Implementation Profile

With a completed gap analysis, the team is ready to create a program-specific implementation guide—a fully constrained profile that leaves no ambiguity as to which data elements should be sent and how they should be conveyed to meet the specific data requirements for the newborn screening program.

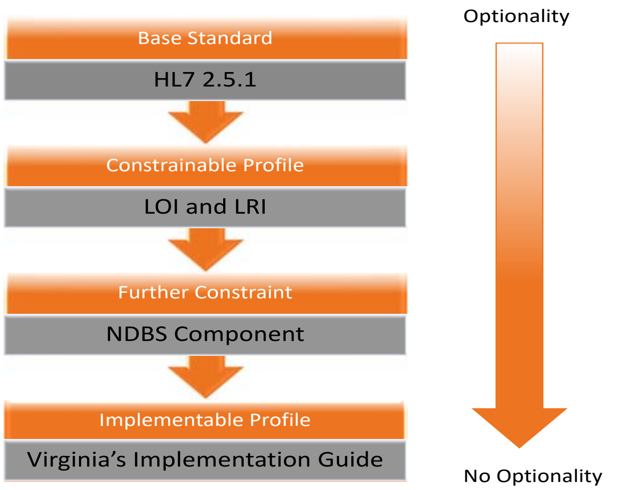

Figure 2: Creating a Fully-Constrained Implementation Profile

While there is no formal specification for how implementation guides should be formatted, most often, they mirror the look of the profile from which it is based. The document is edited where needed to reflect specific changes for the program. The implementation profile may include additional guidance or clarification on various parts of the document as needed. When we think of creating a constrained profile, however, many times we are referring to changes made to the static definition, which defines requirements for how the message should look in terms of segments and fields. For a more detailed description of how to create an Implementation Profile specific to the laboratory, see Tool #3, Baby Steps .

2.5 Define Conformance

As shown in the last several sections, a great deal of effort can go into not only understanding the standard but also applying it to a specific use case and then, finally, a specific implementation. The driving force behind this work is the idea that creating a granular, unambiguous definition allows both senders and receivers to build to a common goal, thereby reducing the need to maintain unique interfaces.

With so much effort put into defining the "rules," it is understandable that "conformance," or the adherence to agreed-upon rules, is paramount. At a basic level, the statement of conformance profiles in the message header says, "These are the rules I play by" and, by extension, "If you play by the same rules, we can play together."

However, what happens when the program is not able to follow the rules? For example, due to program business processes or application differences, the laboratory may be unable to support a data element that is identified by the NDBS component as required. Or alternatively, the laboratory may need to use a data element that has been marked as not supported (X). Using a strict interpretation of the rules, the laboratory will have been deemed non-conformant to the NDBS component. What does this really mean, and should you be worried?

The uniqueness of NBS programs in the United States makes the creation of a one-size-fits-all option almost impossible. The NDBS component should accommodate most programs with additional constraint of any "open" fields presumably making up the difference. Nevertheless, in reality, not everyone will be fully conformant and deviations from the standard may be negotiated between sender and receiver and thoroughly documented in the implementation guide. A certain amount of nonconformance is acceptable, as long as you have documented your deviations and all partners are in agreement.

> "The NBS Program learned that the hospitals were not capturing all fields in the Electronic Medical Record, and our staff needed to make some decisions about how critical those missing fields were to laboratory results. It stretched the staff to think outside the box regarding what they needed to have versus what was nice to have."
>
> Newborn Screening (NBS) Program at the Minnesota Department of Health (MDH); Case Study #3

The implications for those who must demonstrate conformance may be more significant. For example, vendors building to your specification would need to modify their specifications to promote a product as "conformant."

2.6 Identify Vocabulary

In the previous sections, we have looked at the profile or definition—specifically the segments and fields that structure the message. How these data elements are populated, however, is equally important and is determined by the field's format i.e. the datatype. Datatypes provide a standardized way of sending information such as names, dates, addresses, text, and more. Information conveyed using standardized identifiers are 'coded'.

Coded values, often referred to as "vocabulary," are essential to achieving interoperability. Just as HL7 provides a common structure, coded information allows sender and receiver to agree upon a common representation of data within that structure. For example, standardized codes for sex remove ambiguity. Senders and receivers can be confident that transmission of an "M" will always stand for Male, "F" for Female, etc. The values that may be used for each coded field are determined by the value set, a collection of codes that dictates the allowable content. The following resources provide more information on the existing standardized vocabulary that the NBS message will use:

- Value Set Companion Guide – This document defines detailed value sets for each field of the LOI and LRI guides. These values are expected to apply to the profile unless it has been specifically documented otherwise.

- Newborn Screening Coding and Terminology – The National Library of Medicine (NLM) has defined codes specifically for Newborn screening test Panels. Of particular value is the panel of Laboratory Observation Identifiers Names and Codes (LOINC), which the laboratory will need to describe many of the concepts contained in the result message.

The availability of the Value Set Companion guide and the NBS Coding and Terminology Guide is a great help, as standard codes have already been identified for the clear majority of concepts relevant for newborn screening orders and results. With the variability of data collected across newborn screening programs, however, it is unlikely that the existing value sets will adequately cover every value collected by every state.

When this occurs and an existing code is not available, local additions to a value set are permitted. Additions must be clearly communicated between senders and receivers and should be well documented. The Lab Value Set Companion Guide offers implementation guidelines which may be helpful for programs going this route.

As you map standard codes, it may be helpful to keep in mind the following:

- Where will my vocabulary be maintained? Within my laboratory information management system (LIMS) or within my integration engine?
- Who will be responsible for maintaining (updating, adding, retiring) values?
- Will you have a change management process for vocabulary?

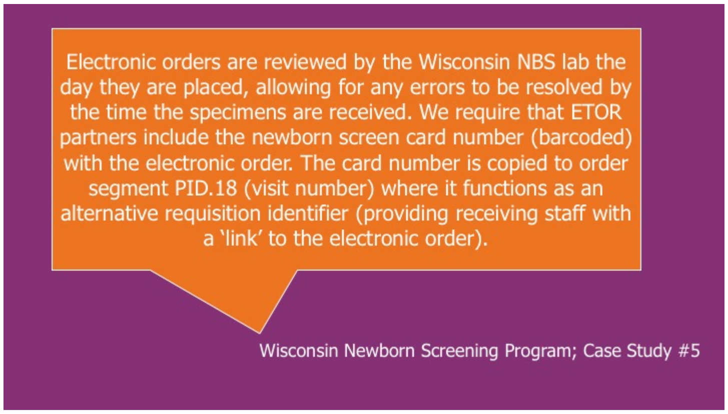

The work of mapping your local values to standardized codes is important—it will determine if you are accurately conveying the intended information. A vocabulary mapping document has been created to aid with this task. It includes NDBS associated value sets for fields relating to patient demographics, treatment history, order details, and results, among others.

3. *Identify a Technical Solution*

Summary

It may be necessary to revise the scope of the project based on the level of effort involved in modifying and integrating the laboratory's technical architecture. Therefore, the project manager should meet with IT subject matter experts who are familiar with the lab environment early in the project planning phase. The IT personnel should be familiar enough with the data entry process in a hospital or lab setting to identify the technical solution that the implementation project will use to process and parse an incoming message.

The level of effort needed for implementing these changes will vary greatly depending on whether or not the laboratory has an existing messaging infrastructure. The team may be able to re-use an existing infrastructure with little or no modification. However, if this is the laboratory's first messaging project, the technical development can take months to complete. Additionally, the contractual or staffing resources within the laboratory may require that a vendor or other third party complete the changes to the LIMS or other software applications; the schedule of these entities may also impact the timing of the development.

Dependencies	Initiate Project	
Personnel Resources	Business Analyst<br>Project Manager	Technical SME (lab or hospital data entry)<br>LIMS Admin
Timeline	Depending on the team's familiarity with electronic messaging, the design of the technical solution may take weeks or months to complete. This activity can be accomplished in parallel with other tasks and can be phased through the project as needed to meet the lab's implementation goals.	
Tools	Architectural Diagram	Message Flow Diagram
Key Outcomes	By the end of these tasks, the project team should have identified and planned out a technical solution, including a list of development work that will need to be accomplished.	
Case Studies	None	

Tasks

3.1 *Gather Technical Documentation*

The project team should assemble the available internal documentation that will allow them to fully understand the current technical architecture within the organization, initiate a gap analysis, and design an appropriate technical solution. The technical architecture is the set of systems and associated people and processes that will enable the current workflow to process NBS orders and generate and send results. Typically, this is a combination of automated and manual processes that span multiple systems or applications, including order entry applications, the LIMS, and systems involved in message transport, among others.

Artifacts that may be useful as the project team designs a technical solution include:

- The NBS collection device(s) or cards that the hospital uses to collect patient information and submit blood samples for testing.

- Current test result report format (may be PDF or digital).
- The Laboratory Orders Interface (LOI) message specification that the hospital will use to send the NBS order to the laboratory.
- The Laboratory Results Interface (LRI) message specification that the laboratory will use to send results to providers.
- An example data extract from the LIMS that the laboratory uses to generate the current test result report.
- Any diagrams, workflows, or other information that the laboratory has outlining or describing the systems which comprise the laboratory's technical infrastructure and how these systems are integrated.

3.2 Design Technical Solution

Once the team has gathered the appropriate documentation, it is time to design the technical solution that the laboratory will use to receive orders and send results. This design will translate the message flow diagram developed earlier in the project initiation and planning phase (Initiate Project) into a model technical architecture. The technical team will also rely on the results of both the gap analysis performed in Chapter 2 and the Workflow Analysis performed in Chapter 4. In addition, the team should plan to review the proposed solution with at least one partner hospital. The success of the design hinges on the ability and willingness of hospital partners to modify their own systems to support the data flow.

The key artifact developed by IT during this task is a holistic architectural diagram of the laboratory systems that will be involved in each step of the message generation and transport (for results) and the message receipt and import (for orders). The design should specify the use of such components as integration engines or data warehouses and explain the intended transport mechanism along with other enterprise services.

The architectural approach taken will vary based on multiple factors. It may be that the laboratory's technical architecture includes more than one system (e.g., the LIMS and a data broker such as Rhapsody or Mirth, or even custom code) that can perform the necessary functions. There is no one right answer. Some LIMS have native functionality to support terminology mapping and/or HL7 message processing. The availability and type of technical expertise will also impact the architectural decision. If the LIMS is not maintained by in-house staff, changes can be complex, costly, time-consuming, and subject to the scheduling of external resources. In this case, it may be more efficient for functions such as terminology translation and HL7 segment generation to be accomplished outside of the LIMS with either a broker or custom coding. Similarly, if centralized IT or outside vendors support data brokering, it is important to build a solution architecture that is configurable wherever possible. The appropriate solution will accommodate software and security constraints and will ideally build on the strengths of the laboratory's technical team. Note that message formats, standard codes, and validation rules change over time and must be maintained. A table-driven approach that minimizes hard-coding in the data broker will allow for easier and less expensive changes to the message.

In general, it is recommended that the laboratory adopt an architectural approach that is modular and loosely coupled. A system that is loosely coupled uses components that do not rely heavily on the design or definition of components in other systems. This allows for a single component to be changed without requiring changes to other components. The goal of such an architectural approach is to insulate the LIMS or other source systems from changes to the message and terminology. See Figure 3: Sample

Architectural Diagram below for a very simple model of an architecture. The diagram should clearly identify the following functions, along with the system performing those functions.

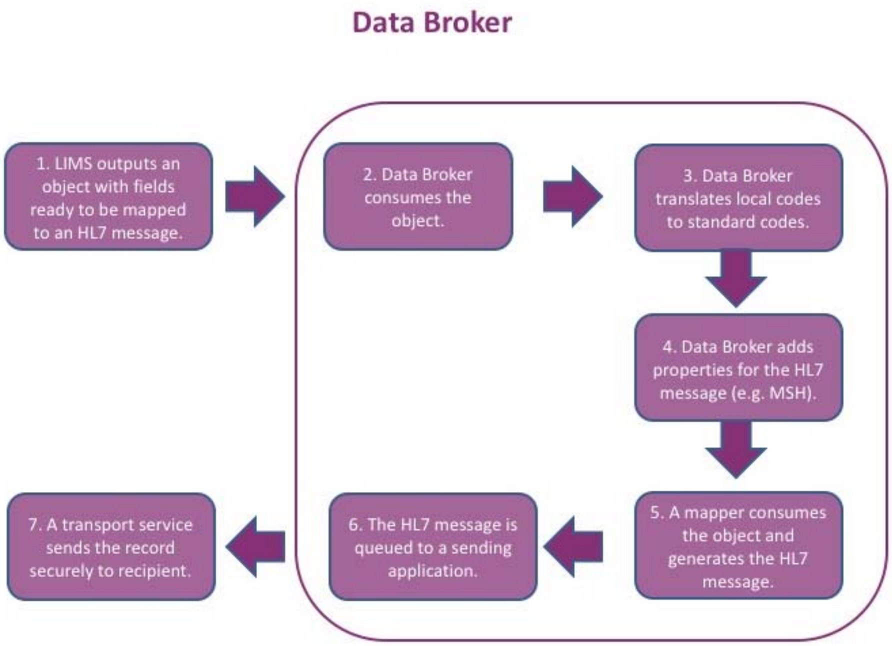

Figure 3: Sample Architectural Diagram

A. Existing Components of the Technical Architecture

The first factor to consider when designing a technical solution is the existence or absence of a current messaging solution at the laboratory. Most laboratories have developed an infrastructure to support standardized messaging of reportable conditions such as Electronic Laboratory Report (ELR) or Laboratory Response Network (LRN) standardized messaging. To the extent possible, the existing messaging infrastructure should be leveraged for NBS messaging.

Laboratories may encounter challenges in several areas when trying to repurpose the existing infrastructure:

- Many laboratories can ***send*** result messages from the LIMS, but NBS orders require that the LIMS can ***receive*** order messages.
- Incoming messages present additional security challenges for the technical staff as most IT departments are reluctant to create a hole in their firewall and open themselves up to cyber attacks.
- Many laboratories use a different LIMS for NBS than they do for other testing. Thus, the NBS team may not be able to leverage the model for data extraction and HL7 mapping from existing messaging projects.
- While the laboratory's existing technical architecture and messaging capabilities will inform the technical solution that is designed, keep in mind that it may be necessary for the technical team to develop new components as part of the overall solution.

B. Standard Terminology Mapping, Translation and Maintenance

Local codes and terms that are stored in the LIMS will need to be translated to standard codes as identified in the HL7 Implementation Guide. The technical design should account for where and how within the laboratory's architecture this translation will be accomplished.

C. Data Import / Extract from Source System(s)

The technical solution should indicate how the data contained in the order message will be imported into the LIMS (or other systems) and how the data needed to create the result message will be exported out of the LIMS (or other systems). The approach will depend on native LIMS functionality and the technical expertise available.

D. Transformation of Source System Data Elements

The data extract must be transformed into a structured HL7 message. Similarly, the incoming message must be transformed into a format that the LIMS can consume. In most cases, this transformation is accomplished with some type of data broker or integration engine, such as Rhapsody or Mirth.

E. Secure Transport / Receipt of Messages

The effective exchange of HL7 messages necessitates a bi-directional and secure messaging platform that can provide a common approach to security requirements (such as encryption and authentication), as well as a standard method for addressing and routing content. Such exchanges also require auditing capability and a consistent way to send and receive data exchange confirmations.

The transport mechanism that the laboratory chooses to send the order and result messages between the laboratory and the hospital will depend on the internal security requirements of both messaging partners. The NBS messaging team should work with the laboratory's internal technical team and clinical partners to determine the best method. Many hospitals prefer to set up a virtual private network (VPN). Other options for transport include CDC's Public Health Information Network Messaging System (PHINMS), Secure File Transfer Protocol (FTP), Direct, or the use of a messaging hub such as a state HIE or APHL's Informatics Messaging Services (AIMS) platform. It would be easier for the laboratory to maintain a single transport mechanism for all NBS messaging, but the capabilities of messaging partners may necessitate the implementation of more than one type of data exchange.

F. Data Privacy

Data that will be included in the NBS messages will include patient identifiable information. Because this information is protected, data security is a high priority. The Health Insurance Portability and Accountability Act of 1996 (HIPAA) and other legislative rules have exemptions for disclosures for public health activities and purposes when that disclosure is to a public health authority, foreign government acting with a public health authority, or a person exposed or at risk of contracting or spreading a disease.[2] The project team will need to consider HIPAA regulations as well as state privacy laws and regulations when designing the technical solution. Care should be taken specifically during the validation phases to ensure that no personally identifiable information is shared with unauthorized personnel or through a non-secure manner such as e-mail.

4. Prepare to Send and Receive Messages

Summary

Preparing the laboratory to send and receive electronic messages is a significant task that will require input and buy-in from multiple stakeholders. The changes needed to support electronic messaging will require updates to the LIMS, as well as modifications to the physical workflow in the laboratory. In many cases, the laboratory may need to work with a LIMS vendor to make these updates. It is very important that laboratory staff work with the vendor to document clear and concise requirements for changes. Upon completion of this chapter, the project team will have:

- Defined the changes needed in the laboratory workflow and LIMS to support electronic messaging at the laboratory.
- Completed the software changes needed to support electronic messaging.
- Configured the data broker to send and receive messages.

Dependencies	Define the Message	Identify a Technical Solution
Personnel Resources	HL7 SME<br>Lab Program SME<br>LIMS Administrator<br>Project Manager	Technical SME<br>Vocab SME<br>Other SMEs consulted as necessary
Timeline	The length of time needed for implementing these changes will vary greatly depending on the scope of the changes needed. It is reasonable to assume that this phase will take several months to a year to complete. It can be accomplished in parallel with other tasks and can be phased through the project as needed to meet the laboratory's implementation goals.	
Tools	APHL Informatics Self-Assessment Toolkit<br>IHE) QRPH White Paper	Requirements Documents<br>Workflow Assessment Tools
Key Outcomes	At the end of these tasks, the team should have documented the "as-is" and "to-be" workflows. The LIMS administrator will have updated the LIMS according to the requirements identified in earlier tasks, and the laboratory systems should be ready to accept and process an incoming order message and/or accept a data extract and generate a valid results message.	
Case Studies	below<br>below<br>belowbelow	

Tasks

4.1 Analyze Laboratory Workflow

Implementation of electronic laboratory orders and results will lead to significant workflow changes for the laboratory. Laboratory staff should expect changes to their job functions, responsibilities and knowledgebase to accommodate the modifications needed to support electronic messaging. Workload may decrease for a member of the data entry staff but increase for a LIMS administrator. The project team should be aware of the stress caused by these changes and engage with staff early and frequently to address concerns and provide solutions. The Tools Reference Guide in Appendix A provides information about Change Management Tools that the laboratory can use to ease this transition.

The first stage in workflow modification is to document the existing workflow and the changes that will need to take place to accommodate the electronic messages. In the long run, electronic messaging will replace manual processes for the majority of samples received, but laboratories should expect to continue to support both workflows to process any samples not submitted electronically (as well as a COntinuity of OPerations [COOP] process). Workflow analyses can be very complex, but at the heart of it is the mapping of a sample's journey through the laboratory. All areas will need to be considered when planning for the new process, including the laboratory workflow itself, the LIMS process, and the physical requirements. The current process is the "as-is" workflow and should demonstrate the physical process of receiving and testing a sample, as well as the staff who interact with a sample and the points at which there are interactions with the LIMS. The project team should take particular note of the processes of receiving specimens, performing demographic entry and reporting result since these will be the areas most impacted by a move to electronic messaging. The project team may want to shadow a member of the receiving, data entry, or reporting teams to be sure the entire scope of jobs duties performed is understood. See Appendix A for Workflow Assessment Tools.

Note: Workflow analyses may highlight other areas of the laboratory that may gain efficiencies with changes to workflow, even if these areas are not directly affected by a move to electronic messaging.

Once the "as-is" process is well understood and documented, the project team should then produce the "to-be" workflow diagrams, imagining what changes will come from implementation of electronic messaging. It is important to note that receiving an electronic message essentially moves the data entry process from the laboratory to the submitter. But, new processes for marrying the physical specimen and the electronic order will be required. The project team should plan how that transition will affect accessioning the sample.

> "In the typical workflow, the lab creates shell requisitions in the LIMS, and the specimens are accessioned simultaneously in bulk. Conversely, in the ETOR workflow, the electronic orders create requisitions, and the specimens are subsequently accessioned individually once they are received."
>
> Wisconsin Newborn Screening Program; Case Study #4

Questions the team might want to investigate at this point include:

- How will the laboratory identify the sample when it arrives? What identifiers will the laboratory use to link the sample with the electronic order?
- What pieces of data must the card include for the laboratory to accept it for testing?
- At what stage in the process will remote data import into the LIMS occur?
- What are the requirements for validating the electronic order imported matches the physical card?
- What are the requirements for acceptance of labels?
- How will discrepancies between the physical form data and electronic data be handled and by whom?
- Who will receive the cards? Will the same staff continue to perform this task after the transition to electronic messaging?

- Where will the receiving process take place? Is hardware available in the physical location needed to support the electronic message? For example, if a sample arrives with a barcode to identify it, are barcode readers available to the accessioning staff?

The project team should ask similar questions about the electronic reporting steps:

- What process changes (if any) are needed to generate electronic results from the LIMS?
- Does laboratory staff use a paper report for review purposes? If so, the workflow will need to provide another option for them to complete their review.
- How will the laboratory handle additional copies of a report?
- Will the laboratory send the report electronically to all recipients, or will the laboratory still need to generate a paper report in some instances?

During this analysis, the project team can document any business decisions that have been or need to be made regarding the sending and receiving of electronic orders. As the questions listed above are answered, these should be documented for future project needs. The Workflow Assessment Tools in Appendix A provide examples of the type of business decision documentation that the laboratory should assemble.

Like the “as-is” workflow, the “to-be” workflow should document who will be responsible for the workflow steps, as well as any interactions with the LIMS. These modifications to the sample processing workflow should drive the changes to the LIMS. Both receiving electronic test orders and sending electronic results messages require the use of the LIMS, and it is vital that the LIMS can support the sending and receiving of messages, both from a technical and usability standpoint. The base ability of a LIMS can vary widely, so LIMS modifications needed to support electronic messaging can also vary. To determine the changes needed (if any), a thorough analysis of the current state of the LIMS should be completed. Note that APHL has developed anAPHL Informatics Self-Assessment that the laboratory can use to assist in this analysis.

First, the project team should evaluate whether the current state of the LIMS can support the “to-be” workflow as documented. The team should document any clear development that will need to be done to support the new workflow. Examples of these types of enhancements can include additional LIMS modules for displaying electronic orders received or the ability to scan the samples received to log them into the LIMS. If multiple programs within the laboratory use the same LIMS, the project team may be able to reuse components and workflows from a program that has already implemented electronic test orders and results. Consolidating workflows will improve cross training, supportability, and data structure within the LIMS.

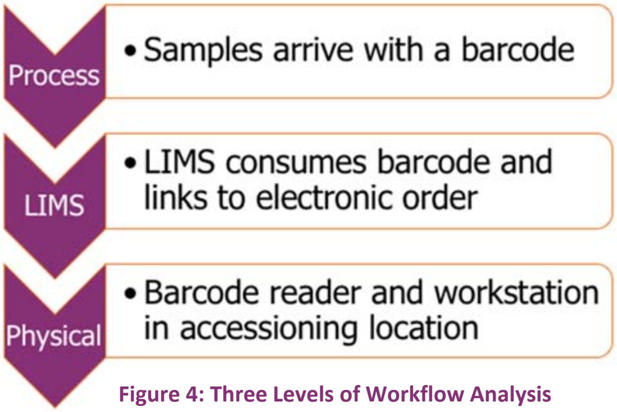

Figure 4: Three Levels of Workflow Analysis

During the evaluation of the “to-be” workflow and the LIMS, it may be helpful to evaluate whether the laboratory has the hardware needed to support this new LIMS use case. The team should document additional reliance on printers, barcode scanners, and workstations. It may be necessary to purchase or rearrange hardware to accommodate the new physical workflow.

Next, the project team should look at the components of the message itself to ensure all

aspects are captured by the LIMS. Importantly, while workflow analysis can begin before the tasks described in Define the Message are complete, the team will need to compare the message developed in Define the Messageto fully assess the LIMS changes required. Therefore, the tasks in Define the Messageand Identify a Technical Solution must be completed in concert. The NBS program may require the message and the LIMS to capture additional data fields. The LIMS may also need the tables needed to maintain the standard code sets identified in Define the Message. To send a results message that will report the results of multiple testing conditions, the LIMS will need capture results of different conditions in a similar manner and have all the necessary flags and triggers built in to alert the report recipient of a critical or abnormal value.

During this LIMS evaluation, it is important to document any interactions the LIMS may have with other laboratory systems. Billing, inventory, and other systems may all be affected by LIMS changes. The project team will need to identify any changes needed in these other systems and add these updates to the project plan.

The considerations identified in this chapter do not represent a comprehensive list of the changes that will need to be addressed during a laboratory process or LIMS workflow change. Rather, the project team should use this list as a starting point for documenting the changes that will be needed in a specific laboratory scenario. As noted in the summary table above and described in the Appendix A: Tools Reference Guide, several tools exist for assisting laboratories with assessing workflow.

4.2 Update LIMS

Once the LIMS changes have been identified, the project team must plan and prioritize these changes. If a LIMS vendor needs to make these changes, the team should engage with them early to assist with requirements gathering and scheduling changes. In some cases, a LIMS vendor will require specific documentation on the required change – often referred to as a Software Specification document (SSD). The team should ask for examples of these documents from the vendor so that they can be sure all the necessary information is included. The project manager will need to work out the details of cost, schedule, and contract with the vendor. The same considerations should be taken into account if the changes will be completed by internal staff. Whether a vendor is contracted, or an internal staff is responsible for the changes, these partners will have competing priorities that will need to be considered in the overall project timeline.

Scheduling the LIMS changes is a vital part of the overall project and should consider the overall project timeline. For example, if implementation of results messaging occurs before receiving orders, prioritize these changes first. The schedule must accommodate the testing and the training of the system users. Before the system is production ready, the LIMS standard operating procedures must be updated and distributed according to the internal laboratory requirements. All of these tasks need to be taken into account in the overall project timeline and budget.

4.3 Configure Integration Engine

Integration engines or data brokers such as Rhapsody or Mirth provide the capability to automate many steps in the messaging process. Integration engines can be used to map elements from an extract file to the appropriate HL7 segments and fields. Additionally, they can provide automated mapping of local codes to the standard terminology codes required by the implementation guide. Finally, an integration engine can also automate the message workflow by adding the message to the transport queue.

In what follows, we describe a message lifecycle for a data exchange that uses Rhapsody as its integration engine. This example is intended as illustrative. The laboratory can accomplish the same objectives using any available integration engine. A typical Message Lifecycle generally includes the following:

- The LIMS outputs an object (row in database or extract file) with fields ready to be mapped to a HL7 message.
- Rhapsody consumes the data as either an XML message defined by an xsd or a delimited file defined by a Symphonia EDI Parser control file (s3d).
- A mapper consumes the XML or delimited file and generates a HL7 message, in accordance with the implementation guide and based on the mapping lookup tables.
- Rhapsody translates local codes to standard codes as defined in the implementation guides.
- The Rhapsody route adds certain properties to the message, including:
    - Message type (for mapping to condition)
    - Message identifier
    - PHINMS information
    - Message status (errored or valid)
- The transformed HL7 message is queued to a sending application:
    - TransportQ_out table via database filter or communication point to be sent by the PHINMS client
    - Output directory via directory communication point that is polled by a sending application
- A transport service sends the message securely to the receiver.

During this task, the technical team will develop the integration engine functionality as defined in the technical solution to ensure that the laboratory systems are prepared to send and receive NBS messages. In addition, the team will populate the tables and develop the mapper filters to generate a valid electronic message as defined by the chosen implementation guide and applicable business rules.

5. Set Up Validation Method

Summary

The successful processing of order and result messages is dependent on conformance to the implementation profile. The laboratory will need to conduct extensive testing with data exchange partners to ensure that the message reliably transmits the correct information, and that internal validation tools identify and respond to message errors as expected. Upon completion of this chapter, the project team will have:

- Defined the testing needed to ensure the electronic data exchange supports the laboratory's needs.
- Developed a testing plan for the laboratory and its messaging partners to follow.
- Completed unit and functional testing of message validation.
- Planned to engage with hospitals for integration testing.

Dependencies	Initiate Project<br>Prepare to Send and Receive Messages	
Personnel Resources	HL7 SME<br>Lab Program SME<br>Project Manager<br>LIMS Admin	QA Staff<br>Testers<br>Other SMEs consulted as necessary
Timeline	The project team should anticipate spending a few weeks to create a test plan and document the testing needed for verifying the HL7 message compliance. The testing itself may take several months, as testers and developers will need to go through several iterations of messages.	
Tools	Example Message<br>Message Validation Template	Message Validation Feedback Template<br>Smart HL7 Viewer
Key Outcomes	At the end of these tasks, the team should have defined the testing needed to ensure the electronic data exchange supports the laboratory's need by developing a testing plan for the laboratory and its messaging partners to follow. Additionally, they should have completed unit and functional testing of message validation and planned to engage with hospitals for integration testing.	
Case Studies	None	

Tasks

5.1 Test Planning and Performance

A test plan is a comprehensive document or set of documents that lays out how system updates will be tested. Each phase of testing should have an individual or team responsible for its satisfactory completion. Test plans are designed to make sure the production system will be useful and accommodate testing scenarios. See the Tools Reference Guide for sample Test Plan components.

The test plan should include several categories of testing:

A. Unit and Functional Testing

Project staff should complete unit and functional testing internally before any other partners are engaged in the testing. This stage of testing will assess the major functional changes and developments made to meet the requirements of the implementation guide. Comparison to the paper report or submission card provides a baseline for expectations of what data should appear in the results message and order message.

Even before partners are fully engaged in the project, the laboratory should simulate and test its ability to receive an order message. The LIMS should be capable of loading an order message that meets the specifications in the implementation guide. The HL7 SME will need to assess each field to make sure it is populating in the correct place in the LIMS. This review can be a difficult task and requires a significant amount of patience and attention to detail, but it is an essential part of validating the incoming message.

In planning for this testing, the project team should consider the test cases they will need to perform. Examples of test cases may include a repeat test card, a collection card that is unsatisfactory, and cards that are linked to previous cards. Identification and testing of these scenarios is important for creating a workable electronic messaging system.

Once the team has completed updates to the LIMS and message broker, they should evaluate the HL7 messages produced for its adherence to the guide specifications. In this testing phase, the team should identify scenarios that represent certain testing outcomes, such as critical, abnormal and normal values, rejections and unsatisfactory results. The test cases should assess each scenario to ensure that the correct report values, alert flags, and triggers appear in the message. Like the testing performed for the consumption of the order message, this testing will be time-consuming and highly detailed. The testers should expect to have to revisit the development stages as issues are noted and retest the messages multiple times to identify all issues. The Tools Reference Guide provides evaluation and issues tracking tools (i.e., the Message Validation Template and the Message Validation Feedback Template) that may help the laboratory accomplish these tasks.

B. System and Integration Testing

System testing ensures data is properly sent and received between systems. The laboratory will need to develop a plan that tests the integration between its systems and those of its relevant messaging partners, including hospitals and health departments. If the LIMS interacts with any other laboratory systems, such as a billing system, inventory system, or others, the laboratory should also plan to test these integrations during system testing. Internal system testing can and should be performed before the partners are engaged so that when partner agencies are ready to test, the laboratory is confident that the internal process will be stable.

During system and integration tests, all the portions of the system(s) are tested together. Common and unusual scenarios should be identified and tested; some testers may think of this as a time to try to "break" the system. The team should review areas of concern or previous corrective action from paper reporting process during this time to make sure the electronic system will support these scenarios. Again, the project team should be prepared to revisit development efforts as issues are identified and retest once corrected.

Once system testing is complete and acceptable, the project team should engage with partners to perform parallel testing. Parallel testing involves running both paper and electronic data processes. The team should compare these data and note and correct any discrepancies as needed. The lab should

request screen shots of the hospital systems at order entry and at results receipt to ensure the data in the message is accurate in comparison to the hospital system. Generally, the team should not have to revisit any development efforts at this stage, since system testing should have identified most development issues. The laboratory usually performs parallel testing for a certain period of time until all parties are comfortable that the electronic system can fully replace the manual process.

C. Performance and Stress Testing

The laboratory should perform stress testing to verify that the system integration has the capacity to support the volume of testing required. This testing should simulate the peak volume of users and samples in the system at one time. Any degradation of system function or response should be noted and reported to the infrastructure team members to evaluate and correct.

D. Resources and Expertise

The test plan should identify responsible testers for each testing phase, as well as who will be responsible for approving the system for production. The development team should also be engaged at this phase since they will need to address any issues found during testing. The plan should also document how issues will be reported and the process for revisiting development so testers have a clear path for resolving issues.

While planning for testing, the project team should engage closely with the laboratory quality assurance (QA) staff. Close consideration should be given to making sure that the testing and associated documentation meet the laboratory's QA and accreditation requirements.

Section I Conclusion

By completing the steps outlined in Section I, the project team prepares the laboratory's personnel, technical infrastructure, and messaging capabilities to receive and send electronic NBS data exchange. At the end of this process, the project team will have determined the following:

- The scope, timeline, and overall management plan for the implementation project and the outreach approach for engaging hospital partners.
- The implementation profile that the laboratory will use to define the NBS electronic message.
- The changes that laboratory will need to make to the existing laboratory workflows and LIMS.
- The laboratory system(s) involved in receiving and processing the incoming test order message, as well as those generating and sending the outgoing results message and how these systems will integrate.
- The test plan that the laboratory will use to verify that the data exchange process is stable and the messages are valid.

While the laboratory may have to revisit and revise the decisions and artifacts developed in Section I, the preponderance of this foundational work should remain stable throughout the project lifecycle and while completing the subsequent activities described in this Guide.

It is important to stress again that the laboratory should not approach the tasks in each Chapter or even in each Section in a completely linear mode. The laboratory may be able to start certain Section II and III activities before all Section I activities have been completed. In some cases, a specific hospital partner may have been involved from the beginning of the process and have been invested in the planning and development of the electronic message. The hospital may be able to take preparatory steps of its own during this period. The key is that that the laboratory cannot fully implement the data exchange with

any hospital until the basic messaging framework has been established. Furthermore, as the laboratory begins to engage and onboard multiple messaging partners, it will be more efficient for the laboratory to already have a solid outreach strategy in place and be ready to begin implementation work. Section II describes this outreach.

Section II: Managing Relationships with Hospitals

6. *Partner with Hospitals*

Summary

When planning to implement a data exchange with hospitals, it is critical for the project team to think through the process that the laboratory should follow to engage the potential external messaging partner, confirm commitment, and communicate the exact specifications for the message and its transport. This planning will ensure consistency, although engagement levels will likely differ somewhat for each hospital.

The activities described in this section transcend many of the other tasks described in the Guide. A key to a successful exchange begins with the laboratory initiating a conversation with hospitals as soon as they begin to consider implementing NBS messaging. Moreover, the relationship with each hospital must be maintained throughout the entire project lifecycle and beyond, even after the data exchange is in production. Cooperation and buy-in from hospital partners is essential to the success of the NBS messaging project and, in general, should be secured before work begins in earnest at the lab.

This section provides a set of best practices and tips, collected in interviews with representatives from both laboratories and hospitals. Based on the particular circumstances within its jurisdiction, the laboratory must decide when and how much to engage hospitals in each step of the project, from initial planning, to designing a technical solution, to developing the message, and for continued operations and maintenance. This section can serve as a resource for those decisions.

Dependencies	Initiate Project	
Resources	Lab Leadership<br>Project Manager	Other SMEs consulted as necessary
Timeline	While the tasks described in this chapter will not require a significant level of effort on the part of the laboratory, they may take longer than any other step in the guide in terms of calendar time. Note that the project manager can initiate outreach and planning with hospitals even before Section I activities are completed.	
Tools	DURSA<br>Hospital Contacts Template<br>Informational Package<br>MOU	Partner Assessment<br>PHII Communications Toolkit<br>Project Charter<br>Stakeholder Matrix
Key Outcomes	The team should have a package of material to share with hospitals when the laboratory begins outreach to potential data exchange partners. The laboratory should have identified a project champion at the hospital who will help drive the project. Both parties should agree on an implementation timeline and the details of the data exchange, including the message guide and the technical solution. The hospital and laboratory should complete the appropriate partnership documents, such as an MOU or DURSA.	
Case Studies	below<br>below<br>below	

Tasks

6.1 Identify Hospitals

The first step in engaging hospitals is to identify possible hospital partners and discuss the opportunity with them. This outreach may take the form of individual conversations, a public webinar or both. Next, the laboratory should screen hospitals to determine whether they are ready to begin implementation. Keep in mind that hospitals may need to undergo a lengthy internal process to authorize the project. Therefore, outreach to hospitals may begin while the laboratory is still completing the activities described in Section I. Indeed, as the laboratory pursues funding to implement NBS electronic data exchange, it may be helpful to have already identified interested hospitals and have obtained an expressed commitment from them, such as a signed letter of intent.

> "Establishing and maintaining the pilot exchange of ETOR required a significant investment from both partners in the relationship, including changes in workflows."
>
> Wisconsin Newborn Screening Program; Case study #2

A. Who and How Many

It is simply not possible for the laboratory to onboard NBS messaging with all hospitals in the state at the same time. Therefore, the laboratory will need to develop a plan to engage with messaging partners. It is advisable to keep the scope limited at first. The laboratory's target milestones will determine the approach to prioritizing hospitals. The laboratory may prioritize hospitals based on the hospital's readiness or interest, the proportion of state births that the hospital represents, or other factors. If a key metric of the implementation is based on converting a certain percentage of NBS testing to electronic data exchange, or on covering a certain percentage of births, the laboratory may need to aggressively court these larger hospitals.

The laboratory may consider onboarding a single pilot hospital before expanding NBS messaging to other hospitals. In selecting this pilot, the laboratory may leverage an existing relationship with a hospital, particularly if the hospital has already worked with the laboratory's informatics team on another project or is known to have a savvy and proficient team.

Even after the first hospital is in production, the number of hospitals that the laboratory can implement simultaneously will depend on the resources available and on the capacity of the laboratory's informatics team and systems. The laboratory may consider assembling a group of hospitals that use the same EHR to effectively create a community of peers who are all implementing NBS messaging at the same time. Conversely, the laboratory may consciously work with a group that uses different EHRs to ensure that the chosen technical solution will work ecumenically.

The laboratory can coordinate with statewide associations and workgroups to reach specific hospital partners. The statewide hospital association may be able to help identify appropriate hospitals based on their profiles (e.g., birth volume, EHR systems, etc.). At a minimum, the association can disseminate information about the NBS data exchange project to member hospitals and gauge interest. Statewide EHR workgroups may serve a similar purpose. The laboratory may also consider scheduling a webinar to

discuss the project in more detail with the entire hospital community and solicit participation; the associations can help publicize the event. Ideally, hospitals will respond to this initial outreach and approach the laboratory. Hospitals that take the initiative are more likely to remain committed and see the implementation through to completion. However, it may be necessary for the laboratory to reach out more proactively to targeted hospitals that have a high sample volume or that serve a large proportion of the population.

B. What to Tell Hospitals

Whatever the approach to reaching out to hospitals, the laboratory will need to develop a presentation and an informational packet to share with potential messaging partners. The purpose of this packet is to introduce the hospital to the project at a high, non-technical level. The material in the packet should explain the public health purpose of the project, the obligations of the messaging partner, and the potential benefits for the hospital. The laboratory may use brochures, articles, webpages, or other marketing materials to convey this information. The project champion within the hospital can use this informational material to help hospital leadership understand and authorize the project. As an example, the Virginia DCLS has made Informational Package available to potential hospital partners through its website.

The laboratory should provide whatever information it can to help the hospital estimate the personnel resources and costs associated with this project. In general, the hospitals we interviewed consider the implementation of HL7 ETOR messages for NBS to be a fairly modest effort. The level of effort will depend on the technical solution that is chosen, the messaging capabilities of the hospital, and on whether the laboratory is using a third-party software vendor to stand up the interface. In addition to IT work, the nursing staff will need to assess and make changes to existing workflows. Staff will need to be trained on the new protocols, and on any new software applications that the hospital adopts. The project manager may ask hospitals to track the effort involved in onboarding NBS messaging so that the laboratory can make these (anonymized) metrics available to other partners.

Importantly, the packet should include material that reviews the public health importance of newborn screening and its impact on healthcare. In many cases, hospital staff performs NBS as a matter of routine and do not consider the broader implications. By educating the staff on the impact of NBS to patient outcomes, the laboratory can increase cooperation.

The packet should also stress the potential for NBS messaging to gain efficiencies, improve timeliness, eliminate clerical data entry, and ensure that results reach the right provider to facilitate timely follow-up, diagnosis, and treatment for affected newborns. In short, NBS messaging has the potential to save money and save lives. Some laboratories have developed a focused document that summarizes the return on investment to illustrate what hospitals gain by implementing electronic messaging.

Some laboratories have incentivized hospitals to participate in NBS data exchange by offering test kits or other inducements. If funding incentives are available, the team should have a clear plan for how to direct these incentives to partners. Additionally, the laboratory can offer hospitals softer incentives, such as publications, presentations, or other means of recognition. The informational package that is provided to hospitals should describe any incentives that are available.

C. Assess the Hospital's Readiness

As the laboratory begins working with a hospital, it is advisable that the laboratory assess the hospital's readiness to implement NBS electronic data exchange. The laboratory can request that the hospitals complete a partner assessment and, where appropriate, review the responses during an initial call.

> Lessons learned include when developing timelines, the team needs to consider competing EHR related projects and the availability of facility staff, such as network engineers for VPNs and ADT testers. The understanding and expertise of hospital staff influence the speed of a Newborn Admission Notification Information (NANI) implementation.
>
> Oz systems; Vendor Case Study #2

The assessment may be a high-level review of the hospital's systems and setup to confirm whether they have the minimum technical capabilities to implement this data exchange. The assessment should also inquire about any upcoming large-scale upgrades or releases that may affect the proposed timeline. If applicable, the assessment should ask if the hospital is interested in working with a third-party vendor to expedite the implementation process.

The laboratory may decide to delve more deeply into requirements in the assessment and initial call and review the message guide to evaluate the hospital's ability to meet the requirements in the constrained guide. Such a discussion should focus on the gaps that exist and whether the hospital would have to make significant EHR updates to address them. The laboratory may want to suggest third party vendors who will work with hospitals and assist in their onboarding and implementation. Third party vendors (ex. Oz) may be very good solutions for hospitals who do not have internal IT resources suitable for completing this project.

6.2 Communicate with Partners

It is highly recommended that the laboratory identifies and engages a project champion at each hospital who will advocate for the project, increase accountability, and function as the primary contact of communication between your institutions. This individual may be a member of laboratory or IT leadership, or a NBS nurse manager. Preferably, it should be someone who understands the hospital's NBS workflow and data exchange capabilities, as well as its administration and governance.

The project champion will help the project manager identify other contacts within the hospital who will work with the laboratory throughout the various stages of the project. In time, the hospital's technical architects and network engineers will need to set up the connection with the laboratory, and program staff will help review and validate test messages. Note that a Hospital Contacts Template has been included as part of the Building Blocks Toolkit in order to simplify the process of identifying the members of the hospital team. The project manager should update the stakeholder matrix with this information. As the project proceeds, it will be important to have a clear understanding the roles and responsibilities of each person.

It is important to maintain regular, open communication with the hospital throughout the implementation and once the data exchange is in production. During the implementation, the laboratory may choose to organize a peer call with all hospitals that are currently implementing NBS messaging, but individual checkpoints with each hospital are generally a more effective means of discussing and resolving issues, communicating progress, and spurring tasks towards completion.

It is a best practice for the laboratory to regularly solicit feedback from hospitals on the overall onboarding process and all aspects of the data exchange. The project manager may consider scheduling a post action interview with each hospital after the data exchange goes live. The laboratory can apply any lessons learned to improve the process with future partner hospitals. The project team should also review and update the onboarding package based on this feedback so that hospitals have the information they need to make decisions.

> In order to learn about the processes, challenges, and timelines of the electronic data exchange project at each hospital, the Virginia NBS Program needed to communicate with the hospital project team regularly…The Virginia NBS program has had success participating in shorter meetings with individual hospitals versus a monthly extended meeting with all project participants.
>
> Virginia Newborn Screening Program; Case Study #6

The laboratory will need to maintain the relationship with hospital partners even after the data exchange has gone live. Primarily, the laboratory must keep a list of hospital contacts up to date to troubleshoot any issues with the data feed. In addition, the change control system that the laboratory sets in place must include mechanisms for processing change requests from hospitals, communicating changes to the appropriate staff within the hospitals, and working with the hospital team to update the message. See Section IV: Operations and Maintenance for further discussion of the continuous monitoring and improvement activities that the laboratory will conduct.

6.3 Mitigate Delays

The most significant obstacles to completing the implementation are often not related to the technical aspects of the data exchange. Many factors can cause delays: the hospital may schedule an upgrade to the EHR or new leadership in a key position may choose to revise the project plan. Nevertheless, in conversations with us, laboratories returned repeatedly to three aspects of working with hospitals that can adversely affect the timeline of the project. First, the laboratory must convince hospital leadership to authorize the project. Second, the hospital must execute the relevant data sharing agreements, which requires a thorough legal review. Third, the project may linger on the schedule of the hospital's IT department for months while the IT resources are devoted to work orders with a higher priority.

A. Project Authorization

The hospital's project champion must guide the hospital through an internal process to review, approve, and authorize the project. This process can be quite time-consuming, with approval taking on average at least 6 months after the kickoff call with the laboratory. Based on the hospital's organizational structure and policies, the champion may need to obtain separate buy-in from different stakeholder groups, including IT, legal, the childbirth center and other interested programs. The laboratory can support the hospital during this process by providing an informational package that describes the purpose of the project and the responsibilities of the hospital. The laboratory may need to work with the hospital team to prepare a business case specific to the hospital, deliver presentations to hospital leadership, provide input on other documentation that the hospital develops, or answer questions. In particular, it may be helpful for the business case to identify cost savings for the hospital, as well as any incentives or disincentives for implementing the data exchange. The laboratory may also reach out to practicing neonatologists and local pediatricians in the area that can testify to the potential benefits to their patients and the need to make this project a priority.

B. Partnership Documents

As early as possible, the laboratory should clearly articulate the requirements of the project to hospital partners. The hospital will be expected to make resources available to work with the laboratory to implement and test the data exchange. The hospital's nursing, IT, and laboratory staff must work with the laboratory to roll out the new process and troubleshoot any issues. It is important for hospital leadership to understand and acknowledge the scope of the work required to fully implement the agreed upon data exchange. In at least one instance, the hospital cooperated to get result messages flowing from the laboratory to the hospital, but then demurred building order messages to share with the laboratory.

The laboratory may consider trying to formalize the hospital's commitment through a written agreement such as a project charter, even if this document is not legally binding. Laboratories have reported mixed results with this approach, as some hospitals decline to sign any agreement without subjecting the document to an arduous review process. Nevertheless, it is important for all parties to understand the role and responsibilities of the stakeholders involved.

Other scope decisions that need to be made include how to handle follow up and confirmatory tests or alternate collection locations. The logistics of these and other situations will need to be assessed, planned for, understood and agreed on by all parties. Laboratory leadership will also need to determine how flexible to be with hospitals in terms of amending the process and the message. The goal of NBS electronic messaging is to reduce the time and resources involved in sending and processing NBS test orders and results; the laboratory should carefully weigh any accommodations that the laboratory decides to make against this goal. It is unwise for the laboratory to appease hospital demands if the modifications create unmanageable accessioning processes or convoluted workflows. The case studies included in this Guide illustrate some strategies for balancing these concerns.

Once the project is authorized, the laboratory and hospital will probably need to sign other partnership documents such as a memorandum of understanding (MOU) or a Data Use and Retention Agreement (DURSA). The exact documents that will need to be executed will depend on the legal arrangements between the data exchange partners. At a minimum, the partners will need a messaging policy document of some kind that will lay out how the data will be treated, who owns the data, the security and HIPAA concerns of both parties, and other particulars. See Identify a Technical Solution and Establish Connectivity for more information on the specific details these agreements should contain. The laboratory may also consider signing a service level agreement with hospital partners to establish the support that the laboratory will provide once the data exchange is in production. The hospital's legal team will need to review these agreements in detail. Even if hospital leadership is on board with the project, the legal review can continue for several months. Often, the execution of the partnership documents constitutes the longest delay for the project.

Note that the requirements for data sharing agreements vary by jurisdiction. If state law requires that hospitals share data with public health agencies, the laboratory may not be obliged to sign data sharing agreements with hospitals. The project manager should consult with the agency's legal resources to understand the ramifications.

C. Hospital IT

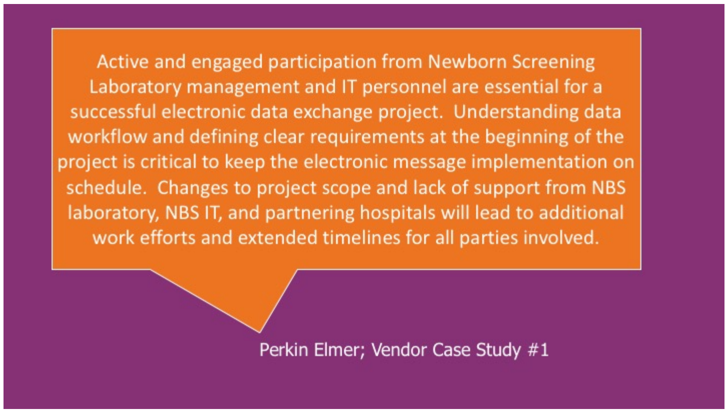

Once hospital leadership has signed off on the project, the hospital will need to prepare its systems and perform its own workflow analysis. If not already done, the hospital will have to review the constrained message guide closely to identify gaps in the current data extract. The hospital may need to update its EHR to address data gaps and implement the technical solution that the hospital will use. Depending on the IT structure, competing priorities, and the availability of program SMEs within the hospital, it may take a considerable amount of time to accomplish these tasks. In interviews, hospitals projected several weeks of actual work to implement the data exchange, but it may take several months to get the project on the IT project schedule. To the extent possible, the project manager should encourage the hospital to make healthcare impact of the project and the needs of the hospital’s nursery and laboratory system the priority, rather than deferring the project to accommodate the IT department.

Section II Conclusion

Working with hospitals can be challenging. This section highlights the elements of this partnership and describes some strategies from which the laboratory can draw when navigating this relationship. Expect delays. The laboratory should endeavor to identify project champions within the hospital who believe in the project and can influence decision makers. Fortunately, the hard work of establishing relationships with these individuals can pay off later, as the laboratory will be in a better position to move other data exchange projects forward with these partners.

Section III: Implementing a Data Exchange with a Messaging Partner

Section III Introduction

This section describes the general process that laboratories will follow when implementing NBS data exchange with a hospital or other messaging partner. First, as described in Section II: Managing Relationships with Hospitals, the laboratory must work with the hospital to plan the implementation, sign any partnership documents, and agree on the specific details of the technical solution (transport method, routing, etc.) that the hospital will use. Next, the laboratory will need to support the hospital's IT team as it establishes and tests connectivity with the laboratory. The messaging partners will then need to develop and validate test messages to ensure that the data each receives meets system and program requirements. Finally, the partners transition the data exchange to production and discontinue the legacy method of sending test orders and results. The steps in this section will likely need to be completed separately with each hospital or hospital system.

7. Establish Connectivity

Summary

Much of the effort to establish connectivity will have been accomplished in the preparation and planning stages. The technical effort is most likely the least time-consuming aspect of this implementation. The laboratory should have defined several artifacts prior to establishing connectivity:

- A messaging policy document
- A security policy review
- Technical design document
- A test plan (See Set Up Validation Method)

The technical approach to connectivity and transport will vary from laboratory to laboratory and possibly from hospital to hospital. Transport options include PHINMS, Secure File Transfer Protocol (SFTP), Web Services, Direct, Virtual Private Network (VPN) and Health Information Exchange (if available).

Testing and establishing the connectivity should take days while the preparation may take weeks or months. It will involve technical staff from both the laboratory and the hospital. Preparatory work will include security personnel as well as administration from both the hospital and laboratory.

Dependencies	Identify a Technical Solution	Set Up Validation Method
Resources	HL7 SME<br>Technical SME	Project Manager<br>Other SMEs consulted as necessary
Timeline	Preparation for establishing connectivity between the laboratory and hospitals can take weeks or months, while the implementation should only take a few days.	
Tools	DURSA, MOU, or other messaging policy document	
Key Outcomes	Transport capable of secure, bi-directional exchange of test orders and results	
Case Studies	None	

7.1 Develop Messaging Policy Document

The first artifact required for connectivity is the messaging policy document (e.g., an MOU or DURSA). It should document confidentiality, authorization, and non-repudiation constraints of the messaging system. For each electronically ordered newborn screening specimen, the laboratory will receive an electronic data set as well as the data set received on the physical form. The messaging policy document should outline how the laboratory will handle and prioritize these data sets. It should also document requirements for protecting personally identifiable information (PII) per state laws and regulations. The messaging policy document may describe policies, practices, and specific third-party security packages such as firewalls, intrusion detection software, and proxy servers that may impact the system.

It should identify the approach for encryption of payload as well as authentication of sender. The messaging policy should clearly define what data is required in the HL7 message and what data will be required on the order card. Additionally, the document should include a process to handle missing and/or conflicting data, as well as information about the intended retention and archiving strategy. It is recommended that the laboratory retain original HL7 order messages for at least six months.

Finally, the document should include an approach or strategy for uniquely identifying an ordering entity. Object Identifiers (OIDS) are recommended as a best practice. The messaging policy document should also define what constitutes a unique message (the key field or fields) as well as a unique order to avoid duplicates and handle updates correctly.

7.2 Review and Sign Off on Security Policy

The NBS messaging team should review existing IT Security Policies at both the laboratory and hospital. It is recommended that any security policy decisions are documented in the messaging policy document and approved by both the laboratory and hospital. Depending on the policies in place at the agency, it may be necessary to have a security officer or the laboratory director involved with this review and sign-off process.

7.3 Establish and Test Connection

Note that the initial connection between the hospital and laboratory will be used to execute the test plan as described in Set Up Validation Method. The testers must affirm that the data exchange and all test messages have passed validation before the laboratory establishes a connection in production. To test the connection, it is recommended to begin with a simple non-encrypted text message ("Hello, world"). Both parties essentially send a test to ensure that they can send and receive data from each other. Further testing should include validation of the encryption method. Various error test cases (malformed message, incorrupt file, data transmission interruptions) should also be performed to ensure the system handles errors correctly.

> This pilot project showed that the infrastructure for sending electronic results could be set up quickly and at little cost. This process could easily be expanded to send electronic results to multiple hospitals. Currently the data is being sent in PDF format, but the same infrastructure could be used to send HL7 messages.
>
> Ohio Newborn Screening Program; Case Study #8

8. Complete Message Validation

Summary

Completing the message validation will rely heavily on the implementation guide and test plans completed in Section I. At the end of this phase of implementation, the laboratory will be ready to exchange messages with its hospital partner. The laboratory will need to complete these tasks with each hospital. The process may change slightly, depending on the specific needs of each partner. As the laboratory repeats this process with multiple partners, it should document frequently encountered issues and the methods used to resolve them as well as any other lessons learned. This documentation will increase efficiencies and reduce the time and effort needed to complete the validation and onboarding process with other partners. If working with a third-party software vendor or through a state HIE, additional testing will be required to test the interface between hospital EHR and the third-party software, as well as the message transport between hospital and state laboratory.

Dependencies	Section I: Getting Ready (all)<br>Establish Connectivity	
Personnel Resources	HL7 SME<br>Lab Program SME<br>LIMS Administrator<br>Project Manager	Technical SME<br>Testers<br>Vocab SME
Timeline	The team should expect to spend at least a month validating messages with the partner hospital. This validation will be required of every partner that is brought on board.	
Tools	Message Validation Feedback Template<br>Message Validation Template	Communication Plan
Key Outcomes	The messages developed by the laboratory and hospital will have passed validation according to the criteria set up in the Test Plan.	
Case Studies	None	

Tasks

8.1 Perform Test Cases

Refer to the test plan (Section I, Chapter 5) to determine the test cases that need to be run during this phase. The laboratory should prepare and discuss a list of scenarios with the hospital partner. Due to the unique requirements of the partner hospital, some modifications to the plan may need to be made before beginning this process. These test cases will need to be run in a test system to avoid any confusion with real patient samples.

The laboratory may already have entered test samples into the LIMS test environment if they have on-boarded hospitals previously. The hospital can enter test cases into its EHR test environment even while the IT team is working on setting up the technical infrastructure to transmit the message. During this process, the project manager must ensure that the laboratory and hospital testers are clear about how to report issues identified during testing. To resolve these issues, the laboratory tester will need to coordinate with the tester on the hospital team as well with the developers and integration specialist on the laboratory team.

The team will need to test both incoming orders and outgoing results messages. Scenarios for orders and results should have been determined in the test plan.

A. Consume Test Orders

The first step in validation will be the ability to consume test orders that the hospital generates. The hospital partner should mock up orders in their test EHR that correspond to the test case scenarios defined in the test plan.

Once the order arrives at the laboratory, testers must verify that every field arrives as expected in the LIMS. Note some fields are kept "behind the scenes" and are not visible through the LIMS user interface, but by working closely with the integration SME, the tester can identify these scenarios. The tester must also make sure any defined triggers are working correctly. For example, if the test case requires the order be linked to a previous submission, the tester should assess this linkage, as well as any differences in test orders that may be required because of the linking.

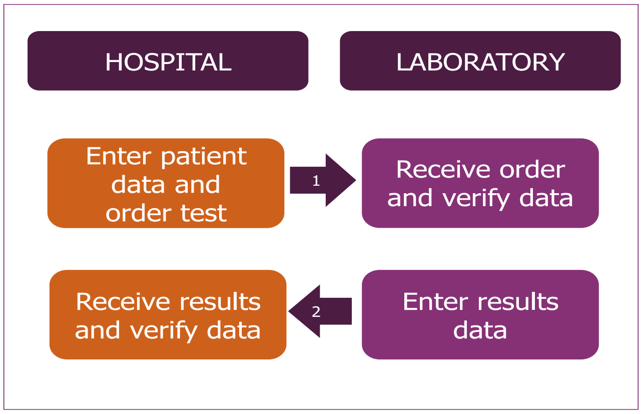

Figure 5: Thoroughly Test Mock Orders

During this phase of testing, the laboratory tester will have to work closely with the partner hospital to address any issues identified. Additionally, the tester must keep documentation on the results of the testing as well as any issues that arise and how they were addressed. Working closely with the quality assurance staff at the laboratory, the tester is responsible for producing the documentation needed to meet accreditation requirements.

B. Send Test Results from LIMS

The second phase of message validation is to run the tests through the LIMS and release results. The tester should have a mechanism to view the HL7 results messages and ensure the messages meet the specifications of the implementation guide and the test cases. The laboratory should have completed much of this testing before engaging the partner hospital, but the tester should still check every field for compliance with implementation guide and reporting requirements for the laboratory. Once verified, these messages should be sent to the test system of the partner hospital for verification. The laboratory should be in constant communication with the hospital to ensure that the messages were received and are being consumed correctly in the EHR.

Again, the laboratory tester should be responsible for documenting the results of the validation efforts. The laboratory may ask the hospital partner to send screen shots of the data as it appears in the EHR to record as part of the validation packet.

8.2 Incorporate Changes

All issues identified during the validation phases should be recorded. The laboratory tester should work closely with development staff to assist in addressing any issues. The laboratory development staff should also be prepared to work closely with their counterparts at the hospital to assist in resolving issues, if needed.

In some cases, the issues identified may reveal limitations in the hospital or laboratory systems that cannot be resolved by coding and development. The laboratory and hospital should work together to find business solutions to these issues, if needed. The resolution to each issue, whether it is a development effort or a business decision, should also be recorded to make sure any deviation from the test plan is understood. Some issues identified will be classified as enhancements to be addressed after the go-live event. The team should be careful to prioritize the issues that are required for go-live, versus those that can be addressed after, and work to maintain the project schedule by holding a hard line on not spending time on "nice-to-have" enhancements to system function.

It is important to note the changes made during the on-boarding of each hospital and to take care in assessing whether any of these changes will affect hospitals that are already in production.

8.3 Re-perform Test Messages

The laboratory and hospital partners should repeat test cases until all issues have been resolved and recorded as complete. In many situations, test cases might have to be adjusted during this process to meet the granularity needed to identify and correct issues, so the team may expect the total number of test cases performed to grow during this process. The team must continue with this iterative process until all test messages pass validation as defined in the test plan. The results of all these tests, as well as any issues and resolutions, should be documented and maintained according to the quality assurance requirements of the laboratory and hospital.

9. Preparation and Go-Live

Summary

Before scheduling the go-live event, all partner agreement documentation should be in place, the validation of the messages should be complete, and all documentation submitted and signed off. By the end of this chapter, the laboratory will have moved the required code into production. The laboratory should have developed a cutover plan during the project planning phase, but this plan may need to be adjusted for each hospital, depending on its particular needs.

Dependencies	Complete Message Validation	
Personnel Resources	Help Desk Staff<br>LIMS Administrator<br>Project Manager	Technical SME<br>Trainer
Timeline	Preparation for go live will take several weeks, but the cutover itself should only take a few hours to move the new code into the production environment.	
Tools	Change Management Tools	
Key Outcomes	The data exchange will be in production for the hospital partner.	
Case Studies	below<br>below	

Tasks

9.1 Train Laboratory Staff in New LIMS Functions

All laboratory staff who are involved in the new process will have to be trained in the new method. By using the tools found in Section I on change management, the laboratory staff should be prepared for these changes. These changes will be most acute as the first hospital is on-boarded to electronic messaging, but as additional hospitals are added, the corresponding changes should be reduced.

The first step in training the laboratory in the new functions is to prepare training materials. These materials should be gauged toward their audience to be most effective. For the personnel most closely involved in the receiving of electronic orders and sending of electronic results, one-on-one training may be best, while other laboratory staff may simply require a group presentation to be informed about the relevant changes that affect them. The project team should assist in the development or editing of formal standard operating procedures to reflect the new process. Laboratory staff may also require quick reference guides, especially for referencing questions that come from hospital submitters.

> "The Wisconsin NBS program worked with their contracted courier to provide differently colored specimen envelopes for the birthing hospitals participating in ETOR. In addition, we placed brightly colored stickers on the NBS cards themselves before shipping them. These two visual cues ensured specimens expected to be associated with an electronic order were identified upon receipt in the NBS lab."
>
> Wisconsin Newborn Screening Program; Case Study #4

The project team may be able to develop some training materials through the validation process, but in order to make sure the most up-to-date information is included in the training material, these should be finalized after the validation process is complete.

The project manager should ensure that all laboratory staff, including those who work outside the newborn screening laboratory, are aware of the updates in the NBS lab. If any unintended consequences are realized after the go-live event, such as dips in bandwidth or delays in other electronic messaging, the laboratory should know whom to contact with questions or issues.

As with the performance of the test cases, the laboratory trainers should document staff training to make sure the quality assurance requirements of the laboratory are being met. Staff with new job functions should be assessed for competency, and the results should be documented in their training records.

9.2 Cut Over to Production

A. Schedule Go-Live Event

The team should schedule the go-live event carefully, ensuring all essential personnel are available to assist. As with many of the tasks in this section, the on-boarding of the first hospital will require more attention during the go-live than subsequent hospitals. After identifying the essential personnel needed during the go-live the team can identify a time for the deployment. Management should be aware of the need for overtime and should secure preapproval for the staff, if needed. The laboratory team should know with whom to communicate at the partner site, both the IT and the administrative contacts, and how to reach them to resolve issues.

Frequently, moving new code into production will need to be scheduled after hours so not to affect the laboratory's daily processes negatively. If possible, the team should plan on testing basic function of the system after the deployment to ensure there are no negative consequences of the production move.

B. Develop Transition Plan

Well in advance of the go-live event, the project manager should have developed a transition plan. This will need to be reviewed with all affected personnel who will be responsible for the operation and maintenance of the system. The plan should also include any knowledge transfer activities and may need to be tweaked to accommodate the needs of each individual hospital. For example, depending on the needs and preference of the partner hospital, the team may adjust the length of time and scope of parallel reporting (both paper and electronic). During parallel testing, the team should rely on a clear communication plan between the laboratory and the hospital to complete and report the comparison between the paper and electronic reports. As with the test cases, the team should document any issues and their resolutions during the parallel testing phase.

9.3 Close Out

A. Turn Off Paper Reporting

Once parallel testing is complete, the laboratory team will need to cease the production of paper reports from their LIMS for that site. This may require significant LIMS changes or minor configuration, depending on the LIMS and its capabilities. The laboratory testers should validate this change to ensure all reports that should be printed are continuing to print, and those that are not, do not. The project manager should make sure the changes are clearly communicated to the staff responsible for reporting so that they are prepared for the change of job function.

B. Retire Legacy Systems

If, during the close out, legacy systems are retired, the team should make sure all data retention policies are met. The team must decide if the legacy data will be migrated to the new system or archived for retrieval using a different method. The team must clearly communicate to staff how the data must be retrieved from legacy systems.

C. Celebrate

A project of this scope should be celebrated once the goal has been met. The team can use this opportunity to present or publish the work of the team at a conference or in a journal. The project team and laboratory administration should recognize staff who have gone above and beyond for their service to the team and to the laboratory. If possible, include the partner hospital in a celebration recognizing their hard work. Boosting team morale by recognizing the hard work and achievement of the team will be useful to maintain energy for subsequent implementations.

D. Schedule Post Go-Live

During the test cases and parallel testing, the team will likely have identified enhancements to the data exchange function that will need to be addressed. The team should prioritize and schedule these post-implementation enhancements to make sure the system functions as smoothly as possible. The team may need to have several minor production releases after the go-live event to fix issues and provide enhancement to the function rolled out during go-live.

E. Post Go-Live Communication

The project manager will need to communicate clearly with all laboratory staff to make sure everyone is aware that the implementation phase of the project has ended and is moving into the maintenance and operations phase. Depending on the structure of the development team, these same personnel may transition their roles to maintenance and support, or new staff may take over these roles. The team should communicate clearly to the laboratory and partner sites about whom they must contact regarding issues in the maintenance and operations phase so that all staff can be focused on their roles identified after the go-live event.

Section III Conclusion

While the process to establish a data exchange with a message partner follows the same basic steps, the actual implementation is unique for each messaging partner. Setting up and testing the exchange is an iterative process that will require close coordination between the laboratory and hospital. Not only the network and system SMEs but the program SMEs must review and validate the data flow and the messages. The testing and validation process will continue as the teams identify and resolve issues. It is important to clearly establish the requirements for the messaging so that everyone concurs when the data exchange can move into production.

Section IV: Operations and Maintenance

10. Transition to Operations and Maintenance

Summary

Once the laboratory is in production with NBS data exchange with at least one hospital, the project enters a long-term maintenance and operations phase. During this phase, the development team will ramp down activities, and the team will focus on providing support for the users of the message. This phase also provides the opportunity for the laboratory to monitor the new processes for the purpose of documenting improvements to data integrity and turnaround times as a result of the implementation.

Dependencies	Section I Introduction, Section II: Managing Relationships with Hospitals, and Section III: Implementing a Data Exchange with a Messaging Partner	
Personnel Resources	Help Desk Staff<br>Project manager<br>QA Staff	Technical SME<br>Trainer
Timeline	Maintenance and Operations will continue as long as the electronic data exchange is in production.	
Tools	Change Management Tools	Communication Plan
Key Outcomes	The laboratory will have a method and process to successfully maintain electronic data exchange with their partners.	
Case Studies	below	

Tasks

10.1 Communication Post Go-Live

Although the laboratory should have laid out plans for monitoring and support during the planning phase of the project, these plans may have to be modified as the project progresses and more hospitals are participating in data exchange. As with paper reporting, the laboratory should have a clear plan for communicating issues with the partner hospital. These plans should include what type of monitoring the laboratory and hospital is responsible for and how issues will be reported

Since hospitals operate 24 hours a day, many laboratories are moving to a 6 or 7-day work week, with staff running NBS samples all hours of the day and night. Therefore, the laboratory will need to decide how to handle issues after regular laboratory hours. These issues may necessitate setting up an “on-call” schedule for providing real-time assistance or provide a help desk option where issues can be entered remotely by the partner.

Importantly, the laboratory should make every effort to keep the monitoring and support agreements identical for all hospitals to streamline the support provided by the laboratory. The laboratory may want to set up a method for entering and recording issues as they are uncovered and provide a clear method for their partners to report issues. The laboratory will also need to make sure any vendor contracts include this level of support, especially if there is a vendor who is providing IT support for messaging infrastructure.

Identifying the points of contact for each partner is vital to an effective communication plan and the speedy resolution of any issues that arise with the data exchange. The laboratory should assign specific personnel to handle issues within particular domains. For example, an issue with partner connectivity may be handled by IT staff, while an issue with the data may be addressed by a data entry supervisor. Additionally, the laboratory informatician can manage issues with usability, coded values or message structure. Ideally, the laboratory will be able to coordinate with a single point of contact for an entire hospital system who will communicate with each hospital regarding any connectivity or system-wide reporting issues or testing communications. The partner agreements with the hospitals should also include these same considerations, the laboratory should be very clear who to contact at the hospital in case an issue is found.

10.2 Monitor Data Exchange

The laboratory should monitor the data exchange process when it is in the maintenance and operation phase. Monitoring will allow the laboratory and its partners to proactively address issues and record statistics for quality assurance purposes.

Closely monitoring the delivery and receipt of abnormal and critical results will provide both partners with the assurance that the electronic messages are working properly. The laboratory should be aware of factors that might impact the exchange process and actively review the exchange after these events. Factors that might impact the exchange process include upgrades to IT hardware and software, power outages, and any other major disruptions to laboratory or hospital functions. Additionally, the laboratory may want to review any singular, unusual test results, such as abnormal or critical results that are extremely rare, or uncommon combinations of results to make sure the message accurately represents these results.

> "The monthly quality assurance reports provided to submitters by the NBS lab showed a dramatic decrease in the instances of missing key demographic information for the ETOR partners. Internal tracking also showed the elimination of amended reports for demographic changes requested by ETOR partners."
>
> Wisconsin Newborn Screening Program; Case Study #2

Newborn screening programs are encouraged to monitor the number of electronic orders received. By monitoring this information, the laboratory may be able to identify issues at the submitting hospital as well. For example, a sharp decrease in the number of orders received may indicate an issue in the test order process at the hospital. Proactively monitoring these electronic exchange indicators will allow the laboratory and its partners to identify issues and find solutions more quickly.

10.3 Evaluate and Improve Process

Electronic orders and results offer multiple opportunities to improve the quality of the data associated with newborn screening as well as the timeliness of the results. NewSTEPs has established quality indicators to measure multiple factors regarding newborn screening. Electronic messaging may directly affect the indicators related to timeliness and missing data. The laboratory should be able to measure the impact of electronic messaging on these indicators to demonstrate a return on investment.

Quality Indicator 2: Percent of dried blood spot specimens with at least one missing state-defined essential field upon receipt at the laboratory.
Because the implementation of electronic orders can enforce the data included in the message, incomplete submissions can be flagged before the order is sent to the laboratory. Having defined the required fields during the creation of constrained order profile, previous problems associated with missing required data fields should be eliminated by electronic messaging.

Related to this quality indicator, programs should also monitor and provide feedback to hospitals on the quantity of remote ordering errors per facility. Erroneous entry of key data elements into the HL7 order can result in specimen processing delays and a loss of the benefits of electronic ordering. Chronic system wide issues can also be used to identify potential improvements to the HL7 order validation requirements both on the laboratory receiving end and the hospital order entry interface.

Quality Indicator 5: Timeliness of newborn screening activities.
(c) Time from specimen receipt at your state's newborn screening laboratory to reporting out specimen results.
(d) Time from birth to reporting out specimen results.

Electronic messaging should decrease turnaround time for samples tested by reducing the time needed for manual data entry and avoiding the need to rely on the postal system for delivery of results. Additionally, electronic transmission of NBS results should eliminate delays that previously occurred as hard-copy reports were routed internally throughout the hospital after delivery.

By comparing the data from these quality indicators to the values collected before implementation, the laboratory can demonstrate the value of the implementation. The laboratory may also want to collect these data specifically for each hospital submitter. This analysis may reveal a marked difference in quality between hospitals participating in electronic data exchange and those that are not. The laboratory can then use these data to encourage additional hospitals to on-board and, as a proof of concept, to apply for funding opportunities as they arise. Many laboratories share these quality indicator data with their partners through a "report card" that measures the hospital's compliance with meeting data integrity and turnaround time goals. By sharing these quality data with the hospital, the laboratory provides a baseline against which the hospital can improve their practices and ultimately improve the health of its newborns.

Hospital report cards have been shown to be extremely effective in improving process and quality. Laboratories who share these quality data with their partners reported beginning a valuable dialogue with the hospital to provide additional training and services to the partner, as needed. Additionally, some laboratories have provided de-identified aggregate data of all the submitting hospitals, which allows an individual hospital partner to compare its performance to other hospitals in the area.

10.4 Perform Change Control

During the operation phase, the laboratory and its partner hospitals will occasionally need to make changes to the data exchange process. These changes can include new screening tests, changes to the constrained profile, LIMS changes, and hospital system changes. In order to mitigate any adverse effects, the laboratory should evaluate each change in light of the existing process.

The laboratory should consider forming a change control board whose members include laboratory, hospital and IT resources. These members can evaluate the proposed changes for the impact they may have on each of these sectors of the team. The team can review the change and approve or deny it (if possible). If approved, they can lay out a plan for implementation and testing of the change. It is important to include previously on-boarded hospitals as well as those currently being on-boarded so that the team can come to a consensus on the best option for the whole community. The figure below outlines this process. Further details on the format of a change request and detailed change control workflows can be found in the Tools Appendix.

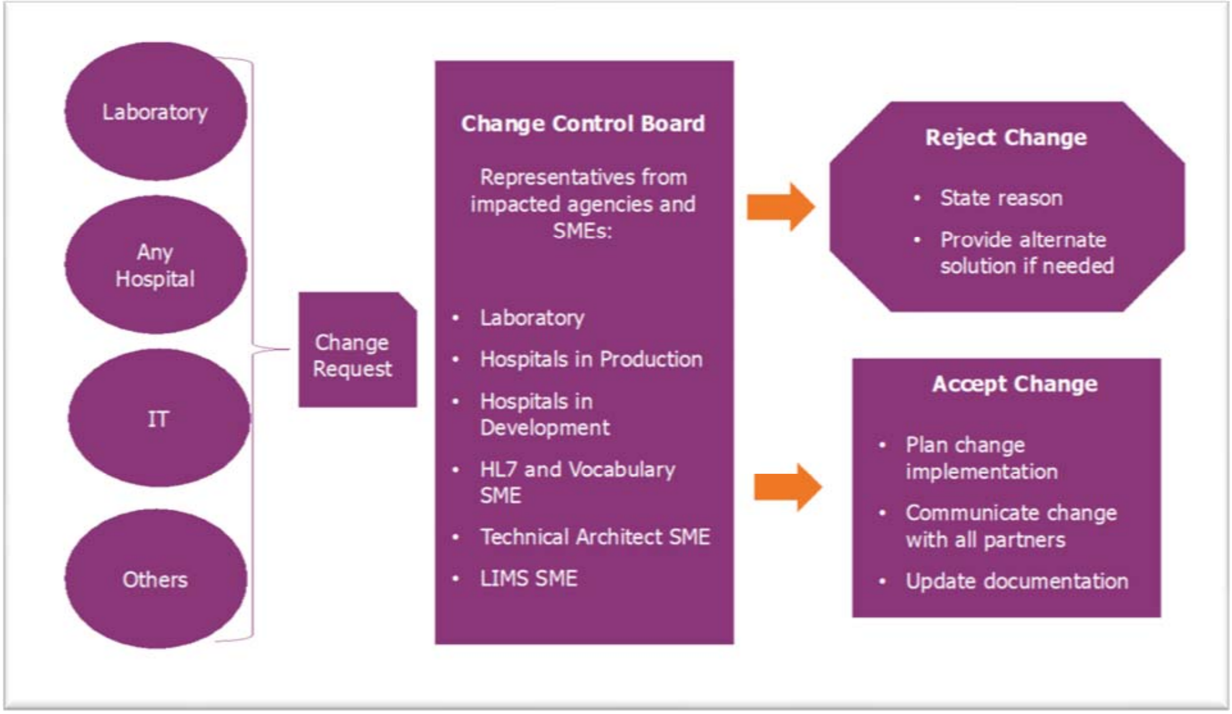

Figure 6: Change Control Workflows

10.5 Fulfill Ongoing Training Needs

The laboratory must determine the on-going training needs of its staff while in the maintenance phase. New employee training, regular competency assessment and updates are some of the artifacts the laboratory might consider including in its training plans. Additionally, the laboratory may want to reach out to its hospital partners to reiterate the importance of newborn screening and following the protocols involved in electronic data exchange. As the hospital experiences staff turnover, establishing consistent communication and reinforcement will provide a solid baseline for continued success.

Strong project documentation is vital for ongoing success of a data exchange project. As laboratory and hospital staff experience turnover, readily accessible and understandable documentation is paramount. The laboratory should consider setting up a document storage and sharing system (ex. SharePoint) to provide a project knowledgebase useful to current and future staff.

Section IV Conclusion

The maintenance and operations phase is an ongoing need for laboratories and their partners. As the laboratory becomes more familiar with electronic messaging and adds hospitals to the program, this effort will become a routine quality assurance task for the laboratory. Strong communication with partner hospitals and a commitment to continuous improvement will provide a long-term return on investment for electronic messaging.

Building Blocks Next Steps

It is the hope of the Building Blocks team that this Guide proves useful for public health laboratories and their partners that are considering or are in the process implementing NBS messaging. As laboratories begin to review and use the Guide, they will likely identify topics that are not covered or that would benefit from a more in-depth treatment. Moreover, we expect the Health Information Technology (HIT) industry to continue to develop and publish tools that the NBS community will find of value. NewSTEPs and its partners therefore consider the Building Blocks Guide a living document and designed the Guide with the hope that the NBS community will continue to expand and add to it over time.

Acknowledgements

The Building Blocks Newborn Screening Health IT Guide and Toolkit is a product of many people and organizations. We actively sought input from many stakeholders and would like to acknowledge and heartily thank those who gave generously of their time and ideas.

The members of the Newborn Screening Health IT Workgroup let us join their monthly calls, listened to our progress reports, and offered many valuable insights and suggestions on the direction of the Guide. Many also volunteered to write up the case studies included in Appendix C documenting their experiences implementing NBS data exchange and working with hospitals. As you will see in the Appendix A: Tools Reference Guide, many laboratories have chosen to share their tools and resources with the broader NBS community.

The Building Blocks team offers a special debt of gratitude to the NewSTEPs 360 awardees who attended the face-to-face meeting in January 2017 to discuss the challenges that laboratories face in standing up an NBS data exchange. The attendees stayed committed and involved throughout this process, and we thank all of them for providing valuable feedback on several drafts of this Guide.

Thank you especially to the staff of the Virginia DCLS for the many conversations with our team, and for letting us in on every aspect of its NBS messaging implementation project. This perspective was critical to the development of the Guide and informed our research. Thanks also to the NewSTEPs 360 meeting facilitators for letting us join the monthly calls. The conversations and especially the presentations by NewSTEPs 360 awardees were very informative. We followed up with several laboratories after the calls to learn more about their initiatives.

Finally, thank you to the editors who made thoughtful suggestions on every version of the Guide, and especially to Willie Andrews, Director of Laboratory Operations at the Virginia DCLS, and Joshua Miller, NewSTEPs 360 Project Manager, for their leadership, support, and contributions.

Abbreviations and Terms

The table below identifies the most common abbreviations used in the Guide. In addition, this table provides definitions and explanations for many of the informatics concepts discussed in the Building Blocks chapters. Please refer to Appendix A: Tools Reference Guide for definitions and descriptions of tools that are referenced in the Guide.

ABBREVIATIONS AND TERMS	
AIMS	The APHL Informatics Messaging Services Platform is a secure, cloud-based environment that provides shared services to aid in the transport, validation, translation and routing of electronic data. For more information, visit https://aimsplatform.com/.
APHL	The Association of Public Health Laboratories represents state and local governmental health laboratories in the United States and works to strengthen laboratory systems serving the public's health in the United States and globally. For more information, visit www.aphl.org.
Change Control	Change Control or Change Management is the process to manage changes that will affect the systems, processes and people supporting Newborn Screening data exchange. A change control process will define how changes will be proposed, accepted, monitored and controlled.
Conformance	HL7 defines a conformance statement as "a claim that the behavior of an application or application module agrees with the constraints stated in one or more message profiles."[•] For our purposes, it is an agreed upon set of rules for how NBS will be exchanged that specifies what data is provided in the message, the format of that data and the associated standard codes.
Constrain	HL7 defines standards to different types of data exchange that can be used across a variety of use cases and stakeholders. The HL7 standard must be further *constrained* to define the specific set of data exchange rules for a given use case and set of stakeholders – NBS for example. The rules in a constrained profile will provide a more granular definition for data exchange that will stay true to the original HL7 definition. Examples include a refinement of required/optional fields, specific value sets for given data elements, additional fields and related business rules. One example is the definition of a specific set of LOINC codes applicable to the test/assays conducted for newborn screening. The HL7 standard simply requires the use of a LOINC code. The NBS profile *constrains* the data exchange to specific LOINC codes.
CSV	A comma-separated values file stores tabular data in plain text. Data partners may need to use a data broker to transform HL7 2.5.1 messages into another file format, such as CSV, before the file can be consumed by the EHR or LIMS.

[•] HL7 Version 2.7 Standard: Chapter 02b - Control; Conformance Using Message Profiles. Available at http://www.hl7.org/implement/standards/product_brief.cfm?product_id=191.

Data Broker	In its simplest form, a data broker is a system or application that provides the capability to transform data from one format to another. For example, a broker can take data in a CSV format and transform it to HL7. Some example data brokers include Orion's Rhapsody, Mirth Connect and Cloverleaf. Please note that the term is used with different definitions in other industries. For our purposes, the data broker is a tool utilized by data senders/receivers to transform data from one format another. Note that the Guide uses the term "Data Broker" interchangeably with "Integration engine."
Data Exchange	Data exchange is the process whereby one stakeholder provides data from its source system in a format that is readable by a receiving system.
Direct	Direct is a national encryption standard for securely exchanging clinical healthcare data via the Internet. For more information, visit https://www.healthit.gov/policy-researchers-implementers/direct-project.
DURSA	Data Use and Retention Agreement. See the entry in the Tools Appendix for more information.
EHR	Hospitals use Electronic Health Record systems to manage patient records. The hospital's EHR is a key system in NBS data exchange.
Electronic Messaging	For our purposes, Electronic Messaging refers to the exchange of standard HL7 messages between a hospital / clinic and a public health laboratory.
ELR	Electronic Laboratory Reporting is generally understood to mean laboratory reports to public health.
ETOR	Electronic Test Order and Result. For the purpose of this Guide, hospitals send the "electronic" HL7 message test order for the NBS panel to the laboratory, and the laboratory returns the "electronic" HL7 message result to the hospital.
FTP	File Transfer Protocol. See SFTP.
HIE	In practice, the term Health Information Exchange typically refers to the organization or system within a region that facilitates the exchange of healthcare data across healthcare providers and public health professionals. The Office of the National Coordinator for Health Information and Technology (ONC) is working with states to establish state HIEs and thereby build the states' capacity to exchange health information quickly and securely. The maturity and scope of these HIEs varies considerably from state to state.
HL7	Health Level Seven is a standards-development organization that provides a comprehensive framework and related standards for the exchange of electronic health information. HL7 Messaging Standard Version 2.5.1 is the most common HL7 standard; it is the standard named in Meaningful Use guidelines.
Integration Engine	See "Data Broker." For our context, the Guide uses these terms interchangeably.
IT	Information Technology
JMC	J Michael Consulting, LLC
LIMS	A Laboratory Information Management System is software that supports laboratory operations and tracks a variety of laboratory data and workflows.
LOI	Laboratory Orders Interface. See the entry in the Tools Appendix for more information.
LOINC	The Logical Observation Identifier Names and Codes is a standard for identifying medical laboratory observations. LOINC codes are a critical part of the NBS ETOR message. For more information, visit https://loinc.org.
LRI	Laboratory Results Interface. See the entry in the Tools Appendix for more information.

Map, Mapper, Mapping	The process of data mapping establishes the relationship between data elements in a source system to a destination system. The mapping definition also includes any rules on transformations required. Patient gender provides a simple example. The source system captures the data in a field called "Newborn Gender." This field must be mapped to the HL7 field "PID-8 Administrative Sex" during message generation. The destination system then maps the data to a field called "Person Gender."
Message Validation	Message Validation is the process of inspecting (either via automated tools or visual inspection) the HL7 message file to evaluate how closely it adheres to the agreed upon data exchange rules in the conformance profile. During this process, validators may identify errors that must be corrected before the message can be processed.
MOU	Memorandum of Understanding. See the entry in the Tools Appendix for more information.
NBS	Newborn Screening
NDBS	Newborn Dried Bloodspot Screening refers to a panel of laboratory tests performed on specimens of blood from a finger stick or a heel stick that are collected onto absorbent filter paper.
NewSTEPs	The Newborn Screening Technical assistance and Evaluation Program is designed to provide data, technical assistance, and training to NBS programs across the country and to assist states with quality improvement initiatives. For more information, visit https://newsteps.org/.
NHIN	National Health Information Network
NLM	National Library of Medicine
OIDS	An Object Identifier is a numeric string that uniquely identifies an object in a directory. Both data exchange partners (the public health laboratory and the hospital) will need to use an OID in the NBS message to identify their organization and their application. HL7 maintains a registry of OIDs. For more information, visit http://www.hl7.org/oid/index.cfm.
Onboarding	The final goal of onboarding a messaging partner is to implement the HL7 message in production and cease the use of the previous mechanism for data exchange. The onboarding process may include steps to ensure that 1) data in the message adheres to the rules and requirements as defined in the NBS Message Implementation Profiles; and 2) the quality and completeness of the data is sufficient for the end user.
Orders	An NBS laboratory test *order* defines the specific tests/assays that are to be performed on the sample. The laboratory order also includes demographic information on the submitter and the patient. Information for the order may be drawn from an HL7 Laboratory Order Message, the physical order form or a combination of both.
PHII	Public Health Informatics Institute
PHINMS	The Public Health Information Network Messaging System is CDC-provided software that employs electronic business rules using Extensible Markup Language technology to send and receive any message type over the Internet securely, facilitating interoperability among myriad public health information systems. The PHIN Tools and Resources site provides specific PHINMS configuration and set-up steps. The laboratory may choose to send NBS messages via PHINMS, or via some other transport mechanism, depending on their internal security requirements and those of their messaging partner(s). For more information, visit http://www.cdc.gov/phin/tools/phinms/index.html.

Profile	An HL7 Implementation Profile is a document that specifies the agreed upon rules for data exchange between stakeholders. A profile further constrains an HL7 Standard to provide more granular rules for a specific data exchange agreement.
Results	An NBS laboratory test result message documents the tests/assays and associated conclusions performed by the public health laboratory.
SFTP	Secure File Transport Protocol is a network protocol and encryption standard that allows data exchange partners to securely access, transfer, and manage files.
SME	Subject Matter Expert
Symphonia EDI Parser	The parser performs a specific function within the Rhapsody integration to convert data from one Electronic Data Interchange (EDI) format to another.
TransportQ_out	A set of PHINMS database tables which store incoming and outgoing messages. For more information, visit http://www.cdc.gov/phin/tools/phinms/index.html.
Virginia DCLS	Virginia Division of Consolidated Laboratory Services
VPN	A Virtual Private Network allows users to share data across a shared or public network as if their computing devices were part of a private network.
Web Services	This data exchange mechanism utilizes web technology such as HTTP to securely transfer files between sender and receiver.
XML	Extensible Markup Language; data partners may need to use a data broker to transform HL7 2.5.1 messages into another file format, such as XML, before the file can be consumed by the EHR or LIMS.
XSD	XML Schema Definition

Appendix A: Tools Reference Guide

Introduction

Appendix A collects all the tools mentioned throughout the *Building Blocks Guide and Toolkit.* For each, we provide a description of the tool and an explanation of when to introduce and apply the tool. Where applicable, we offer publicly available templates and examples that the laboratory can use as a basis for its own project artifacts. The Building Blocks team has also developed certain resources, including an implementation workbook, a mapping document, and an example message, to help laboratories implement NBS data exchange; the list below identifies these resources and how to access them. In addition, several laboratories have graciously permitted us to reference examples of tools that they have developed internally.

Please note that the tools and resources developed by the Building Blocks team are currently hosted on the J Michael Consulting Box site. All resources will be posted to a permanent location on the NewSTEPs website soon.

CDC Unified Process

CDC created a framework and methodology known as the Unified Process to urge project managers to use best practices in the design and execution of their projects.[2] The templates, tools, and other resources that make up the framework enable project managers to adopt practices and processes that comply with good project management methodology and with Federal regulations and policies, including the Enterprise Performance Life Cycle (EPLC) framework. (Health and Human Services utilizes the EPLC in all its projects.)

CDC designed the UP framework specifically with informatics projects in mind, and the mission of the CDC in many respects mirrors that of Public Health Agencies and Laboratories. Therefore, while project management templates are available from many sources, the Building Blocks Toolkit provides multiple examples from the CDC UP.

None of the recommended tools or templates in this Appendix should be considered constrictive or authoritative. Often the laboratory will have a set of project management or other documents that should be used. The use of standard, consistent tools increases the efficiency and effectiveness of project management processes. The source of these artifacts is the decision of the project team, the laboratory and its partners.

[2] For more on the CDC Unified Process, visit https://www2.cdc.gov/cdcup/.

Tools Appendix Index

1. APHL Informatics Self-Assessment Toolkit
2. Architectural Diagram
3. Baby Steps towards Defining the Message
4. Business Case
5. Change Management Tools
6. Communication Plan
7. DURSA
8. Example Budget
9. Example Message
10. HL7 Validation Tool
11. Hospital Contacts Template
12. IHE QRPH White Paper
13. Implementation Guides (LOI and LRI)
14. Implementation Profile
15. Implementation Workbook (LOI and LRI)
16. Informational Package
17. Mapping Workbook
18. Memorandum of Understanding (MOU)
19. Message Flow Diagram
20. Message Validation Feedback Template
21. Message Validation Template
22. Newborn Screening Coding and Terminology Guide
23. Partner Assessment
24. PHII Communications Toolkit
25. Project Charter
26. Project Management Plan
27. Project Schedule
28. Risk Management Plan
29. Requirements Documents
30. Smart HL7 Viewer
31. Stakeholder Matrix
32. Test Plan
33. Value Set Companion Guide
34. Workflow Assessment Tools

1. APHL Informatics Self-Assessment Toolkit

The APHL Informatics Self-Assessment (SA) Toolkit is a web-based tool that the laboratory can use to assess its informatics maturity across 19 capability areas. Users can compare their laboratory's capabilities to that of other, similar laboratories and to the national average. The laboratory can use the SA Tool to identify gaps in current informatics capabilities, which may help to determine the laboratory's readiness to engage in a large-scale data exchange implementation project. The Tool also allows the laboratory to compare its capabilities over time to demonstrate the impact of development projects.

More information

APHL has published the Self-Assessment as both a pdf document and as a web-based tool. The web-based version lets registered users save their work and compare previous assessments; it also features robust visualization tools. Email informatics.support@aphl.org to set up an account.

- Web-based Tool: http://satool.aphl.org/.
- PDF SA Tool form: https://www.aphl.org/mrc/documents/lei_2013jun_informatics-self-assessment-tool-for-phls.pdf.

2. Architectural Diagram

The architectural diagram is a visual representation of the IT systems and infrastructure that will contribute to the technical solution. It depicts the process that the laboratory will use to receive, process, and deliver the message to the LIMS. The design should indicate the transport method and connection point, the integration engine and/or LIMS, and any other systems involved.

More information

See 3.2 for a Sample technical architecture diagram.

3. Baby Steps Toward Defining the Message
This document is a companion to Chapter 2 of the Building Blocks Guide. It describes the steps required for HL7 message definition and provides additional information on the tools and topics discussed in the Guide, including the Implementation Workbook and the Message Validation Template.
**More Information**
The Building Blocks team developed the Baby Steps document as a companion to Chapter 2 of the Guide: https://jmichaelconsulting.box.com/s/53u9zs2rnbup6wundvufv2v477gstwul

4. Business Case
A business case summarizes the justification for starting a new project. It defines the problem that the project is attempting to address and explains the proposed solution. The business case may include a basic cost benefit analysis; it may also review the pros and cons of alternative solutions. The business case is often the first project artifact to articulate and document the objectives of a project, and project sponsors may use the business case to authorize the initiative, or at a minimum to green light additional project planning.
**More Information**
The CDC UP framework provides a useful template for creating a business case. Visit https://www2.cdc.gov/cdcup/library/templates/default.htm to access the UP template library.

5. Change Management Tools
Change management is a discipline that prepares organizations to adopt change. By equipping individuals and teams with the tools needed to be successful, organizational change management increases the success of projects. To successfully implement a project on the scale of electronic messaging, change needs to take place at the level of the individual employee, the organization, and the entire enterprise. Furthermore, these changes need to be managed carefully to maintain employee morale and adoption of the required changes.
**More Information**
Multiple publicly available tools exist to help organizations with planning and executing successful change management. MITRE, a non-profit organization, has published an article on its website that offers a useful and informative introduction to change management: https://www.mitre.org/publications/systems-engineering-guide/enterprise-engineering/transformation-planning-and-organizational-change.

6. Communication Plan
Managing many stakeholders can be complicated. A communication plan documents how and when the project team will reach out to different stakeholders, as well as whose responsibility it is to do the communicating. The Communication Plan may be included as part of the project management plan. The project team should consider developing a communication plan concomitantly with the Stakeholder Matrix during the early planning stages. The team will refer to the Communication Plan continuously throughout the project lifecycle. The project manager may need to update the Communication Plan as stakeholders change and depending on the particular organizational structure of hospitals that are engaged.
**More Information**
The CDC UP framework provides a useful template for creating a change management plan. Visit https://www2.cdc.gov/cdcup/library/templates/default.htm to access the UP template library.

7. Data Use Sharing and Reciprocal Support Agreement (DURSA)
The Data Use and Reciprocal Support Agreement, a document developed by the NHIN Cooperative DURSA Workgroup in 2009, is a specific agreement signed by every participating National Health Information Network (NHIN). The DURSA can serve as a potential model for any multiparty trust agreement. The agreement lays out the responsibilities and expectations of each participant. Among other stipulations, the DURSA points out that all participants are Covered Entities as defined by HIPAA or Business Associates of Covered Entities, and are therefore protected by and must comply with HIPAA privacy rules. It stipulates that the data can only be used for specified purposes, and that each participant is responsible for maintaining a secure environment, and for obtaining necessary equipment and software. A DURSA may alleviate some of the concern that the laboratory or the hospital may feel about sharing data with external entities. In general, a DURSA documents the details of a data-sharing relationship more thoroughly and more rigidly than a MOU.
**More Information**
In 2009, the National Health Information Network (NHIN) IT released a draft DURSA that would govern the exchange of health data on the NHIN. To access the draft, visit https://www.healthit.gov/sites/default/files/draft_nhin_trial_implementations_production_dursa-3.pdf. The 2011 re-statement of the NHIN DURSA, released in 2011, is also available online at http://wnyhealthelink.com/files/pdf/Policies/DURSA_Restatement_1_5.3.11_FINAL_for_PARTICIPANT_SIGNATURE.pdf.

8. Example Budget
It may be necessary during the initial planning phases for the laboratory to estimate the costs of the proposed implementation project. In many cases, it is extremely difficult to accurate calculate the true budget of the project because all of the costs may not be known at the outset. Moreover, the expenses associated with staff time, IT services, software, licensing, HIE connectivity, consultants, developers, etc., will differ for every laboratory. For some laboratories, the effective cost for certain services will be $0. Despite the inherent challenges, the Virginia DCLS has shared an example budget for the Virginia NBS implementation project that offers estimates of the time required from both the Division's IT and laboratory teams.
**More Information**
The Virginia DCLS provided an example budget:<br>https://jmichaelconsulting.app.box.com/s/yeuz5qlv0403hwcfzeenirqik5bhumr3

9. Example Message
While each jurisdiction will create a unique HL7 profile for NBS messaging, it may be helpful for laboratories to review an example order and result message. The Building Blocks team has therefore developed an example of each type of message and populated the Message Validation Feedback Template to serve as a guide.
**More Information**
LOI Example Message: https://jmichaelconsulting.box.com/s/iuujkkmyg24lfzyvy7z4whkq956mzdpr<br>LRI Example Message: https://jmichaelconsulting.box.com/s/r6m2uo5kncbx9ac2lhtcid23ou6s3sww<br>Populated LOI Message Validation Feedback Template:<br>https://jmichaelconsulting.box.com/s/o07dbz2u11bl9fioak6fwa9g9ycld5l1<br>Populated LRI Message Validation Feedback Template:<br>https://jmichaelconsulting.box.com/s/luv4kaml9tyvnnuk7fh2haseyzmflcp0

10. HL7 Validation Tool
The National Institute of Standards and Measure (NIST) hosts several tools and utilities to support HL7 messaging. The laboratory and its testing partners can use the NIST Validation Tool to validate test messages against the HL7 V2 Lab Results Interface (LRI) Guide, Release 1 or Release 2. As of April 2017, the Lab Orders Interface (LOI) Guide had not yet been added to the NIST Validation Tool. Note that the NIST Tool validates messages against the general LRI Guide and does not test the conformance of newborn screening messages specifically.
**More Information**
The NIST HL7 V2 Resource Portal is available at http://hl7v2tools.nist.gov/portal/#/.

11. Hospital Contacts Template
A project roles template identifies the individuals who will perform each required role on the project. The same individual may perform several roles. The project manager may request that each hospital complete this template so that the team has a clear understanding of who is responsible for specific elements of the project.
**More Information**
The Building Blocks team developed a Hospital Contacts Template: https://jmichaelconsulting.box.com/s/n7rkm30vb158vxuvb9w51bl0i9rd8veb

12. IHE Quality Research and Public Health (QRPH) White Paper: Newborn Screening White Paper
The Integrating the Healthcare Enterprise (IHE) QRPH White Paper presents use cases that describe the processes, personnel, and events involved in NBS activities throughout a workflow. It advocates for increased integration and electronic communication across the systems and agencies involved in NBS (e.g., EHRs, LIMS, and surveillance systems). It describes NBS workflows in US Public Health, as well as in France, Germany, and Austria.
**More Information**
The IHE QRPH White Paper is available through the IHE website: https://ihe.net/Technical_Framework/upload/IHE_QRPH_Newborn_Screening_NBS_WhitePaper_Final_2009-09-01.pdf

13. Implementation Guides (LOI and LRI)
The Laboratory Order Interface (LOI) and Laboratory Results Interface (LRI) are HL7 Implementation Guides for creating a HL7 Version 2.5.1 OML^O21 message for laboratory test orders and results, respectively. HL7 Workgroups have created cohesive LRI and LRI Guides specific to the NBS reporting requirements. As of August 2017, these draft profiles are being balloted, with final publication anticipated in late August or early September. These profiles will be of significant value to laboratories that are implementing NBS orders and results.
**More Information**
The ballot versions of both Guides are on the HL7 ballot web site. Note that you will need to create a free HL7 account to download these documents. It is anticipated that HL7 will release the final versions of these Guides in September 2017.<br>LRI ballot version:<br>http://www.hl7.org/documentcenter/public/ballots/2017MAY/downloads/V251_IG_LRI_R1_D4_2017MAY.zip<br>LOI ballot version:<br>http://www.hl7.org/documentcenter/public/ballots/2017MAY/downloads/V251_IG_LABORDERS_R1_D3_2017MAY.zip

14. Implementation Profile
An Implementation Profile is a specification that further constrains the HL7 Implementation Guide based on the requirements of your system interface. The laboratory's IT staff and its messaging partners will need this profile to properly implement the data exchange and to generate a HL7 message that is structurally and syntactically valid. The Implementation Profile should present clear, precise requirements for data format and semantics. The Profile will serve as the gold standard against which the content and structure of test cases are validated.
**More Information**
The Wisconsin Public Health Laboratory has provided an example implementation profile: https://jmichaelconsulting.box.com/s/yfc0xrmhoihl216afysphx6w3nhon5z3.

15. Implementation Workbook (LOI and LRI)
This template merges information from the various sources that define the message format and associated standard codes. It serves as the worksheet to perform gap analysis (existing data vs. requested data) and map to the local data elements. It also serves as the underlying structure for the integration engine, provides mapping from local to standardized data elements, and links to the bound value set for vocabulary validation.
**More Information**
The Building Blocks developed an Implementation Workbook for the LOI order message and the LRI result message.<br>LOI Implementation Workbook:<br>https://jmichaelconsulting.box.com/s/x8ky2k8hvv49d78yhm3bgdu601he5vdp<br>LRI Implementation Workbook:<br>https://jmichaelconsulting.box.com/s/1dp67u2zjvczuwf04mqhmyl5mgx6gnqv

16. Informational Package
The laboratory will need a package of information to distribute to hospitals to help them understand the project and implement NBS electronic data exchange. This package should contain 1) material that introduces them to the project at a high, non-technical level; 2) partnership documents, such as a data use agreement or a memorandum of understanding (MOU), to formalize the relationship between the hospital and the laboratory, and 3) technical information about how to create and validate the messages, cut over to production, and discontinue the legacy feed(s).
**More Information**
The Virginia DCLS developed a comprehensive guide for its hospital partners and has posted it to the agency website for easy access: https://dgs.virginia.gov/division-of-consolidated-laboratory-services/resources/nbs-data-exchange/.

17. Mapping Workbook
In most instances, both the sender and the receiver will need to map standard codes that are used in the HL7 message to local codes used by the native system (i.e., the EHR or LIMS). A mapping workbook that lists out all the required and optional data elements in the message will facilitate the review and collection of this information. The technical team will then be able to use the mapping workbook to populate lookup tables and update system code as necessary to generate the message.
**More Information**
The Building Blocks developed a Mapping Workbook for the LOI order message and the LRI result message.<br>LOI Mapping Workbook: https://jmichaelconsulting.box.com/s/elkraoj6gnh22ilitu5dv4l57obgpe05<br>LRI Mapping Workbook: https://jmichaelconsulting.box.com/s/nda0d49bs7stsxew7gil7h119wpuje9x

18. Memorandum of Understanding (MOU)
A MOU establishes a partnership between two or more entities. While it is not legally binding, a MOU, it is stronger than an unwritten, so-called “gentleman’s agreement.” Its intention is to confirm that all parties have a common understanding of the terms of the partnership. The laboratory may choose to execute a MOU with its data exchange partners or other stakeholders.
**More Information**
The CDC UP framework provides a useful template for creating a memorandum of understanding between two or more parties. Visit https://www2.cdc.gov/cdcup/library/templates/default.htm to access the UP template library.

19. Message Flow Diagram
A message flow diagram is a visual representation of the how the message will travel from the sender’s systems to the receiver’s systems. In this case, it may depict the flow of the message as it is received by and processed by the laboratory’s messaging engine and is then delivered to the LIMS.<br><br>It is important to create a message flow diagram early in the planning process so that all stakeholders understand the process that is being considered, and IT leaders can explain the level of effort involved in setting up the data exchange process. Technical architects will rely on and modify the message flow diagram as they implement the technical solution. The project manager may also use the diagram while in planning and discussion with external partners, including HIE and hospital leadership.
**More Information**
The Michigan and Wisconsin Public Health Laboratories have provided examples of Message Flow Diagrams.<br>MI example: https://jmichaelconsulting.box.com/s/cjydbdpw4eewhiyqdb656o0cski3xuad.<br>WI example: https://jmichaelconsulting.box.com/s/j2p3ij8belcgof5isg0yfbhnn25rt76j.<br>Currently, these examples are hosted on the J Michael Consulting Box site. All resources will be posted to a permanent location on the NewSTEPs website soon.

20. Message Validation Feedback Template
This template documents suggested system updates that would address issues encountered during validation. This template will assist in tracking the issue and resolution or business decision that has been taken because of the issue.
**More Information:**
The Building Blocks team developed a Message Validation Feedback Template:<br>https://jmichaelconsulting.box.com/s/we340y1fyt50vy9b1huux6x5q2q1i85n

21. Message Validation Template
Testers can use these templates during validation to compare the expected value for each data element against the actual content of the message. The LOI and LRI templates are populated with data for the Virginia DCLS order and result message in order to demonstrate how laboratories can utilize this tool. It is expected that the laboratory will replace this data to reflect its own message values.
**More Information**
The Building Blocks team developed a Message Validation Template for the LOI order message and the LRI result message. As further guidance, the Building Blocks team has populated a copy of each template with an example message.<br><br>LOI Message Validation Template:<br>https://jmichaelconsulting.box.com/s/fju541ht5hgse8xr2cmjn6rijs7x94qw<br>LRI Message Validation Template:<br>https://jmichaelconsulting.box.com/s/yb0dgrm0q209hrbtz5q0nrhqyy8h2rsa<br>Populated LOI Message Validation Feedback Template:<br>https://jmichaelconsulting.box.com/s/o07dbz2u11bl9fioak6fwa9g9ycld5l1<br>Populated LRI Message Validation Feedback Template:<br>https://jmichaelconsulting.box.com/s/luv4kaml9tyvnnuk7fh2haseyzmflcp0

22. Newborn Screening Coding and Terminology Guide
The National Library of Medicine (NLM) has defined codes specifically for Newborn screening test panels. Users can peruse or download tables of the codes and value standards that are relevant for recording and transmitting the newborn tests and the conditions for which they screen. This Guide is a valuable resource for the Vocab SMEs at both the laboratory and hospital as they map local to standard codes for the NBS order and result messages.
**More Information**
The LOINC Panel for NBS is available through the NLM website:<br>https://newbornscreeningcodes.nlm.nih.gov.

23. Partner Assessment
Early in the engagement, the laboratory will need to assess whether the hospital hospital's systems and setup possesses the minimum technical capabilities to implement this data exchange. The assessment should inquire about any upcoming large-scale upgrades or releases that may affect the proposed timeline. If applicable, the assessment should ask if the hospital is prepared to work with a third-party vendor. The assessment is also an opportunity for the laboratory to gauge the hospital's overall commitment and interest, collect information about the hospital's EHR, understand, IT structure and process, and identify important points of contact. The laboratory may require the hospital complete and return the assessment, or the laboratory may choose to use the assessment as a script to guide the initial call with the hospital.
**More Information**
The Building Blocks team developed a partner assessment tool that laboratories can modify:<br>https://jmichaelconsulting.box.com/s/nlld7fg4dkfe7uj1btgj6gdtezokl30d.

24. PHII Communications Toolkit
The Public Health Informatics Institute (PHII) recently released the PHII Communications Toolkit, which provides recommendations for how to present and discuss informatics concepts to non-technical audiences. The project team may consider incorporating some of these communication strategies in the discussions with stakeholders.
**More Information**
The full text of the PHII report and toolkit are available at http://phii.org/informatics-communication-toolkit/introduction.

25. Project Charter
The project charter provides a high-level overview of the entire project. It states the objectives of the project, the justification or business need, timeline and resources required, and the critical success factors. In many ways, the project charter builds on the business case. The charter is often one of the first artifacts that the project team drafts and may be used to confirm that stakeholders understand and commit to the project.
**More Information**
The CDC UP framework provides a useful template for creating a project charter. Visit https://www2.cdc.gov/cdcup/library/templates/default.htm to access the UP template library.

26. Project Management Plan
The Project Management (PM) Plan is a document (or set of documents) that helps the team guide the project through its lifecycle, from planning through execution and close-out. It details the project objectives, scope and schedule, and documents the intended approach to managing various elements of the project. By clearly outlining the process for tracking milestones, mitigating risks, or approving changes, the PM Plan instills a formal management approach to project activities.
**More Information**
The CDC UP framework provides a useful template for creating both a project management plan and a project management plan “lite” for short time, straight forward initiatives. Visit https://www2.cdc.gov/cdcup/library/templates/default.htm to access the UP template library.

27. Project Schedule
The project schedule documents the planned timeline of milestones and activities. The schedule may be based on target milestone dates, individualized tasks, or the anticipated completion date of specific project deliverables. Typically, the project schedule includes start and end dates and identifies dependencies. For example, the team cannot start Task 2 until Task 1 has been completed. The project manager can choose to display a schedule in a variety of visual formats, such as a table, a timeline, a calendar, or a Gantt chart.<br><br>A project schedule is an essential tool to think through the activities that need to be completed (and in what order) to accomplish the goals of the project. In addition, the schedule can incorporate external factors, such as vacations, holidays or system upgrades that may affect the overall project timeline. Moreover, the project team will be able to assess the impact of delays on the project by revising dates in the schedule. It is highly recommended that the project team put together a project schedule early in the planning process and update it continually over the course of the project.
**More Information**
The CDC UP framework provides useful templates for creating a project schedule. The templates are available for download in either MS Project or xls format. Visit https://www2.cdc.gov/cdcup/library/templates/default.htm to access the UP template library.

28. Risk Management Plan
Every project is subject to uncertainty. The risk management plan itemizes known factors that may have a positive or negative impact on the project. It estimates the likelihood of the risk occurring, as well as the impact it may have on the project. While some risks are beyond the control of the project team, the risk management plan lays out a strategy for managing these risks and mitigating their impact. The risk management plan often presents this information in a tabular format; it generally forms a part of the project management plan.
**More Information**
Many simple risk management templates exist. Most can be adapted for a project regardless of the project's focus area. For example, a 2014 conference presentation on electric furnace rebuilding presents a useful and free example of a risk management plan: https://soroakoaace2014.wordpress.com/.

29. Requirements Documents
Careful and clear documentation of the requirements needed to implement electronic messaging is vital. The requirements define what is needed to the work of the electronic messaging. These requirements can help prioritize the work to be done and can be referenced in the scope of work of a contractor or vendor.<br>The requirements should define the "what" not the "how" of the system updates, and should be actionable by the system developer.
**More Information**
A 2006 article in *Scientific Computing*, an online computer technology magazine, provides a useful overview of how to define system requirements: http://www.atlab.com/docs/DefiningSystemRequriements.pdf.<br>The Public Health Informatics Institute (PHII) has also published an article on documenting requirements: http://www.phii.org/resources/view/9244/defining-and-validating-system-requirements.

30. Smart HL7 Viewer
SmartHL7 is a "brand" for a set of HL7 tools developed by a software design programmer in Australia. The tools are designed for those working with HL7 standard and protocol. The Message Viewer is a tool for casual inspection of HL7 messages. *(NOTE: Developer does not claim the tool to be standard-compliant.)*
**More Information**
The SmartHL7 viewer is a non-commercial tool that can be downloaded according to the terms and conditions outlined on the website. To learn more visit: http://smarthl7.com/tools.html

31. Stakeholder Matrix
A stakeholder matrix is a tool that allows the project manager to classify the people involved in a project. Typically, this information is presented in a tabular format that maps stakeholders to the amount of influence and impact each has over the project and lays out the strategy for engaging them. The stakeholder matrix facilitates and documents the stakeholder analysis that occurs early in the project planning phase, and should be updated regularly throughout the project lifecycle.
**More Information**
The CDC UP framework provides a useful template for performing a stakeholder analysis. Visit https://www2.cdc.gov/cdcup/library/templates/default.htm to access the UP template library.

32. Test Plan

Creating a test plan is essential to ensuring that system changes are properly tested. By planning out the testing phase, the team can provide the resources and time needed to test and document system functionality before the system is put into production.
**More Information**
The Office of the National Coordinator (ONC) has posted a testing plan template to the healthit.gov website that can be used for any health IT system testing activities: https://www.healthit.gov/providers-professionals/implementation-resources/electronic-health-record-ehr-system-testing-plan.

**33. Value Set Companion Guide**
This document, distributed by HL7, defines detailed value sets for each field of the LOI and LRI Implementation Guides where a coded value is required. These values are expected to apply to the message profile unless the laboratory has specifically decided and documented otherwise. The values in this Companion Guide, paired with the codes in the LOINC Panel for NBS, will provide the laboratory with standard codes for the majority of concepts relevant for NBS orders and results.
**More Information**
The Value Set Companion Guide is available for download on the HL7 website: http://www.hl7.org/implement/standards/product_brief.cfm?product_id=413.

**34. Workflow Assessment Tools**
Workflow assessments provide the basis for determining what changes will need to take place to accommodate the change in process. Documenting an “as-is” and “to-be” workflow shows the existing and future processes and can be used to demonstrate the tasks needed to achieve the new workflow.
**More Information**
The Public Health Informatics Institute (PHII) has developed a diverse toolkit to assist with setting up an EHR-based surveillance program; the toolkit includes tools to understand and accomplish workflow. To access the toolkit, visit: assessment http://www.phii.org/ehrtoolkit.<br>The Wisconsin laboratory created a high-level diagram outlining the workflow changes needed to accommodate electronic messaging:<br>https://jmichaelconsulting.box.com/s/kydluxnxxzr3h6xcd3wfd0xselvfw743<br>Currently, this example is hosted on the J Michael Consulting Box site. All resources will be posted to a permanent location on the NewSTEPs website soon.

Appendix B: SME Matrix

The matrix below defines the subject matter experts identified throughout the Guide and describes the role that each SME performs on the NBS implementation project. This information is organized in the first figure by personnel resource, and in the next by task. The project manager can use this tool to allocate resources for each step of the project. SMEs can use this tool to identify the sections of the Guide that discuss activities relevant to their areas of expertise quickly. Readers can also access the SME Matrix as an Excel spreadsheet to sort and filter task by personnel resource or topic area: https://jmichaelconsulting.box.com/s/tz5e7v2upl9o1ieo75ebbsdgrrybvoo9.

Personnel Resource	Description	I.1 Initiate & Plan the Project	I.2 Define the Message	I.3 Identify a Technical Solution	I.4 Prepare to Send and Receive Messages	I.5 Set Up Validation Method	II.6 Working with Hospitals	III.7 Establish Connectivity with Trading Partner	III.8 Complete Message Validation	III.9 Preparation and Go Live	IV. 10 Operations & Maintenance
Business Analyst	Collects and communicates requirements related to laboratory workflows, system functionality, and message content.	I.1	I.2	I.3							
Help Desk Staff	Assists hospitals and laboratory staff troubleshoot problems with the NBS data exchange.									III.9	IV.10
HL7 SME	Develops message profile that the laboratory will use for NBS data exchange; involved in the creation and validation of HL7 messages.		I.2		I.4	I.5		III.7	III.8		
Lab Leadership	Makes strategic decisions, and allocates resources. May be division chiefs, laboratory directors, and other senior management who need to be involved in major project planning and decisions.	I.1					II.6				
Lab Program SME	Offers guidance about laboratory workflows and processes pertaining to NBS testing.	I.1	I.2		I.4	I.5			III.8		
LIMS Administrator	Updates the LIMS as needed to support the project. Leads LIMS testing and validation efforts.		I.2		I.4				III.8	III.9	
Project Manager	Coordinates the daily activities of the project, monitors progress and risks, and as a liaison with project stakeholders, including laboratory leadership and the hospital project team. May be a member of the leadership team, a laboratorian, an IT resources, or some other SME, this person	I.1	I.2	I.3	I.4	I.5	II.6	III.7	III.8	III.9	IV.10
Quality Assurance Staff	Ensures that the test plan, documentation and quality monitors align with the laboratory's QA and accreditation requirements.					I.5					IV.10
Technical SME	Responsible for designing and implementing the technical solution that the laboratory will use to send and receive electronic NBS message. They are involved in testing and validating the data exchange and test messages, as well as in the onboarding process. Tech SMEs can include network administrators, developers, technical architects, integration engine administrators, and other IT staff.			I.3	I.4	I.5		III.7	III.8	III.9	IV.10
Testers	Executes the test plans and reviews the content and structure of the test messages. These individuals may be drawn from both the tech team and the NBS program.					I.5			III.8		
Trainer	Trains laboratory staff on changes to laboratory workflows and on any new software. Assists in recording staff competency									III.9	IV.10
Vocab SME	Offers guidance about standard codes such as SNOMED and LOINC that will be used in the message; maps local codes to standard codes; involved in the validation of HL7 messages.		I.2		I.4				III.8		

Figure 7: Detailed Description of Subject Matter Experts (SME)

Chapter	Description	Business Analyst	Help Desk Staff	HL7 SME	Lab Leadership	Lab Program SME	LIMS Administrator	Project Manager	Quality Assurance Staff	Tech SME	Testers	Trainer	Vocab SME
I.1 Initiate & Plan the Project	Create project documents, including project management plan; obtain authorization for project; assemble project team				X			X					
I.2 Define the Message	Select HL7 message guide and constrain for laboratory's needs; perform gap analysis, comparing the message against what is the NBS bloodspot card and the LIMS currently; identify needed updates	X		X		X		X					X
I.3 Identify a Technical Solution	Review laboratory's current technical architecture and design data flows for the order and result message			X			X	X		X			
I.4 Prepare to Send and Receive Messages	Update technical architecture and laboratory systems as needed to accommodate the message and proposed data flow; prepare to modify laboratory workflows as needed				X		X	X					
I.5 Set Up Validation Method	Create test plan to verify that the data exchange is functionally robust and that the order and result messages meet all requirements				X		X	X	X				
II.6 Working with Hospitals	Manage relationship with hospital partners; provide hospital team with support and documentation to move the project through legal review and approval				X	X		X					
III.7 Establish Connectivity with Trading Partner	Set up connection point to transport messages between the hospital and the laboratory				X			X		X			
III.8 Complete Message Validation	Review and validate test messages according to the test plan on both the hospital and the laboratory side; resolve issues in iterative process until all messages pass validation			X				X		X	X		
III.9 Preparation and Go Live	Prepare to move the data exchange into production		X					X		X	X	X	
IV. 10 Operations & Maintenance	Continue tasks to monitor and operate data exchange		X			X	X	X	X	X			

Figure 8: Subject Matter Experts (SME) by Chapter

Appendix C: Case Studies

Thank you to all who provided case studies for the Guide. These real-life stories instruct and inspire as they share lessons learned with other NBS programs around the country. We hope you find them helpful and are spurred on to contribute some of your own.

Case Study	Title	Application(s)	Contributor
1	Adapting Tools and Lessons Learned from Other Public Health Programs	Section I, Chapter 1	Minnesota Department of Health
2	Quantifying the Impact of Electronic Test Orders and Results (ETOR) with Quality Assurance Metrics	Section I, Chapter 1<br>Section III<br>Section IV, Chapter 10	Wisconsin Newborn Screening Program
3	Determining Which Demographic Fields Are Critical for Laboratory Result Interpretation and Follow-Up	Section I Chapter 2	Minnesota Department of Health
4	Distinguishing Specimens Associated with Electronic Orders upon Receipt to Facilitate Appropriate Accessioning	Section I, Chapter 4<br>Section III, Chapter 9	Wisconsin Newborn Screening Program
5	Streamlining the Accessioning Workflow for Specimens Associated with Electronic Orders	Section I, Chapter 4<br>Section III, Chapter 9	Wisconsin Newborn Screening Program
6	Maintaining Communication with Hospitals Throughout the Life of a Data Exchange Pilot Project	Section II	Virginia Newborn Screening Program
7	Test Results and Value Coding	Section I, Chapter 2	Michigan Department of Health and Human Services
8	Sending NBS Results in PDF Via Secure File Transfer Protocol (SFTP)	Section III, Chapter 7	Ohio Department of Health
Vendor 1	ADT Message Validation with a Hospital Partner	Section I, Chapter 5	OZ Systems
Vendor 2	Successes and Challenges of HL7 Data Exchange	Section II, Chapter 6	PerkinElmer, Inc.

CASE STUDY #1

by the Minnesota Department of Health

Adapting Tools and Lessons Learned from Other Public Health Programs

BACKGROUND:

The Newborn Screening (NBS) Program at the Minnesota Department of Health (MDH) began implementing electronic reporting of demographic information and point-of-care screening results in all birth hospitals in 2014. For this large project, we needed to track 91 different hospitals who were in various stages of engagement or onboarding.

PROBLEM:

Tracking contacts, project deliverables, status updates, and meeting notes for each hospital or health care system was too complicated for our SharePoint site and network drive. Our team struggled with communication internally.

SOLUTION:

We were put in contact with other programs in our agency who were actively onboarding health care systems for electronic reporting. They had adapted our agency's IT project and ticket tracking software (JIRA) for their specific project needs. We were able to adapt the workflow to our project and track necessary information for all hospitals in JIRA easily.

These programs serve different public health needs and our onboarding projects have different deliverables, but we learned from talking with other programs about pitfalls and successes in informatics. Most tools and lessons learned can be adapted to all public health electronic reporting projects. This is a lesson we can learn over and over again.

CASE STUDY #2

by the Wisconsin Newborn Screening Program

Quantifying the Impact of Electronic Test Orders and Results (ETOR) with Quality Assurance Metrics

BACKGROUND:

The Wisconsin Newborn Screening program piloted the exchange of electronic test orders and results (ETOR) with two birthing hospitals. The electronic order was configured to include all data fields on the newborn screening card that are entered into the newborn screening laboratory information system (LIS), pulling demographic information directly from the electronic health records of the mother and baby, as well as specimen collection information from the hospital LIS.

PROBLEM:

Establishing and maintaining the pilot exchange of ETOR required a significant investment from both partners in the relationship, including changes in workflows at both the hospitals and the NBS laboratory. We needed a means of quantifying the positive impact of ETOR to justify that effort as well as the effort that will be required to expand ETOR to additional partners going forward.

SOLUTION:

With the implementation of ETOR, the Wisconsin NBS program anticipated a decrease in the instances of missing or inaccurate key demographic information, and therefore, also a decrease in amended result reports due to erroneous demographics. In the immediate post-live validation phase of the pilot, NBS cards continued to be filled in completely by hospital staff, even though all fields were intended to be included in the electronic order. During the validation phase, the NBS lab compared all fields included in each electronic order to that provided on the associated card. They tallied all discrepancies and instances of information omitted from the order and provided the information to hospital staff for investigation. The hospital made modifications to workflows and/or the queries pulling the information into the order until it was confirmed that the electronic orders were routinely including complete and accurate information. The monthly quality assurance reports provided to submitters by the NBS lab showed a dramatic decrease in the instances of missing key demographic information for the ETOR partners. Internal tracking of amended result reports also showed the elimination of amended reports for demographic changes requested by ETOR partners.

CASE STUDY #3

by the Minnesota Department of Health

Determining Which Demographic Fields Are Critical for Laboratory Result Interpretation and Follow-Up

BACKGROUND:

The Newborn Screening (NBS) Program at the Minnesota Department of Health (MDH) was in the beginning stages of its interoperability project with one of the largest health systems. During initial meetings with key IT staff from this external partner, questions arose regarding which demographic information from the newborn screening card the hospitals were currently capturing in the electronic medical record (EMR).

PROBLEM:

The NBS learned that the hospitals were not capturing all fields in the EMR, and MDH staff and needed to make some decisions about how critical those missing fields were to laboratory result interpretations and/or short-term follow-up of abnormal results to the primary care provider. To make decisions about this, we set up meetings between the newborn screening operations supervisor, the laboratory supervisor, short-term follow-up supervisor, genetic counselors and senior laboratory staff. This resulted in a re-evaluation of the current demographic fields that were being collected on the screening card but omitted in the EMR, and their importance. It stretched the staff to think outside the box regarding what they needed to have versus what was nice to have.

SOLUTION:

Program staff reached a consensus and decided that many of these fields were important to continue collecting, but a few were not necessary moving forward. We decided that there was not a need to collect antibiotics, risk factors or specific feeding-type fields. The laboratory stated the need for date and time of collection, transfusion status (Y/N answer) and TPN feeding (Y/N answer) for result interpretations. The follow-up team stated the need for primary care provider information for those instances of abnormal result notification. The operations team would require the card barcode for identification purposes. This led to the next step, which was to look at the best method for getting that information to the NBS program via electronic lab ordering.

CASE STUDY #4

by the Wisconsin Newborn Screening Program

Distinguishing Specimens Associated with Electronic Orders Upon Receipt to Facilitate Appropriate Accessioning

BACKGROUND:

For the Wisconsin Newborn Screening program, specimens associated with electronic test ordering and results (ETOR) require a different accessioning workflow than others. In the typical workflow, the lab creates shell requisitions in the laboratory information system and the specimens are accessioned simultaneously in bulk. Conversely, in the ETOR workflow, the electronic orders create requisitions, and the specimens are subsequently accessioned individually once they are received.

PROBLEM:

Specimens associated with electronic orders need to be distinguished upon receipt and excluded from the batch requisition creation/accession process. Failure to do so would create duplicate orders for the specimens, bypass the demographic information sent with the electronic orders and fail to return the results electronically.

SOLUTION:

The Wisconsin NBS program worked with their contracted courier to provide differently colored specimen envelopes for the birthing hospitals participating in ETOR. In addition, we placed brightly colored stickers on the NBS cards themselves before shipping them to these birthing hospitals. Laboratory managers at each of the birthing hospitals ensured the proper envelopes and distinctly-labeled NBS cards were in stock prior to implementing ETOR. These two visual cues ensured specimens expected to be associated with an electronic order were identified upon receipt in the NBS lab and segregated prior to accessioning.

CASE STUDY #5

by the Wisconsin Newborn Screening Program

Streamlining the Accessioning Workflow for Specimens Associated with Electronic Orders

BACKGROUND:

When newborn screening specimens are received in the laboratory, they need to be processed in a timely manner. In the Wisconsin Newborn Screening Laboratory, specimens associated with electronic test orders and results (ETOR) are accessioned individually. Given the increased time to accession individually (compared to the batch accessioning process for non-ETOR specimens), it is essential that the accessioning workflow for ETOR specimens be as streamlined (and error-free) as possible to avoid any additional delays.

PROBLEM:

Errors with electronic orders, including missing orders, can potentially delay specimen processing, as can any difficulties laboratory staff experience finding the appropriate electronic orders for received specimens.

SOLUTION:

Electronic orders are reviewed by the Wisconsin NBS lab the day they are placed, allowing for any errors to be resolved by the time the specimens are received. We require that ETOR partners include the newborn screen card number (barcoded) with the electronic order. The card number is copied to order segment PID.18 (visit number) where it functions as an alternative requisition identifier (providing receiving staff with a 'link' to the electronic order). The card number is preferred over the hospital-assigned specimen number as a 'link' to the electronic order because it is unique. Multiple ETOR partners may use the same specimen numbering format (example: [year]-[day of year]-[collection of day]) and thus it is possible that multiple electronic orders from different submitters may share the same (external) specimen number. Specimens submitted from the ETOR partners are segregated as they are received, and receiving staff immediately confirm a requisition exists for every card using the barcoded card number. This check identifies any specimens received without an electronic order and allows time to obtain the missing order before accessioning. For ease of data entry flow, specimens are accessioned in blocks with those associated with ETOR (i.e. those requiring limited data entry) accessioned last, after all other non-ETOR specimens (i.e. those requiring full data entry) have been received into the laboratory information system.

CASE STUDY #6

by The Virginia Newborn Screening Program

Maintaining Communication with Hospitals Throughout the Life of a Data Exchange Pilot Project

BACKGROUND:

The Virginia Newborn Screening Program partnered with 12 birthing hospitals to pilot the exchange of electronic newborn screening orders and results. The project held a 3-year timeline to go from fact-finding with hospital systems to complete implementation of order and results transmission, with a sustainability plan for implementation of hospital systems beyond the pilot project.

PROBLEM:

In order to learn about the processes, challenges, and timelines of the electronic data exchange project at each hospital, the Virginia NBS Program needed to communicate with the hospital project team regularly. We set up a monthly meeting to include the hospital champions, the Virginia NBS Program, and third-party service providers working with the hospitals. However, low participation by the hospitals on these calls left the NBS Program uninformed and out of touch with their pilot partners. This resulted in a re-evaluation of the communication plan.

SOLUTION:

The Virginia NBS program has had success participating in shorter meetings with individual hospitals versus a monthly extended meeting with all project participants. Some hospitals were already holding regular internal project meetings that the NBS program was allowed to attend. For hospitals not holding project team touchpoints, the NBS program assisted in scheduling and coordinating these meetings. With just one hospital to speak to, sessions now took approximately 15 minutes, which meant more people were able to fit these meetings into their schedules. To maintain flexibility in scheduling these calls, team members shared responsibility in providing NBS program representation on each call. Project-wide meetings have been limited to a quarterly basis to allow hospitals a chance to learn from one-another's experiences since it is expected that they may meet similar challenges.

"Coming together is a beginning. Keeping together is progress. Working together is success." -Henry Ford

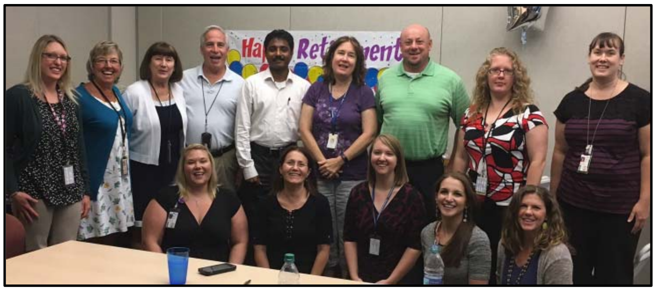

CASE STUDY #7

by the Michigan Department of Health and Human Services

Test Results and Value Coding

BACKGROUND:

The Michigan Department of Health and Human Services (MDHHS) Newborn Screening (NBS) program has been moving toward the goal to implement HL7 messaging for orders and results for several years. Over the last 18 months, we have dedicated additional staff time to the HL7 NBS project. As a part of the implementation, we launched a tremendous effort to map data points for the 53 disorders screened in Michigan from the HL7 build to an associated Logical Observation Identifiers Names and Codes (LOINC).

PROBLEM:

The NBS HL7 project took a long time to gain momentum. At the beginning of the HL7 NBS project, we underestimated the amount of work needed to complete such an endeavor. Additionally, due to competing priorities, we struggled to identify staff with the appropriate level of expertise. We later realized that we needed a person knowledgeable in NBS disorders who *also* had a working knowledge of the laboratory information management system (LIMS) to perform the data mapping.

SOLUTION:

We employed a laboratory scientist who was cross-trained in all MI NBS test areas to work on the HL7 project. After assigning an individual with the appropriate level of expertise, the project was back on track.

MICHIGAN Newborn Screening

MDHHS Michigan Department of Health & Human Services

CASE STUDY #8

by the Ohio Department of Health

Sending NBS Results in PDF Via Secure File Transfer Protocol (SFTP)

BACKGROUND:

Ohio receives 140,000 newborn screening specimens each year. Once testing is completed, results are reported to the birth facility, facility collecting the specimen, primary care providers, and medical specialists providing care to the child. In 2016, more than 303,000 results were reported by the newborn screening program. The majority (approximately 90%) of these were faxed with the remainder being sent by US mail. Due to the high cost of faxing, the Ohio Department of Health (ODH) was exploring alternative methods of reporting.

PROBLEM:

In September 2016 ODH reached out to a large pediatric hospital system to discuss alternatives to faxing newborn screening results. The hospital system had previously shown interest in receiving newborn screening results by HL7. A test message of newborn screening results (formatted following the National Library of Medicine) was sent to the hospital. After reviewing the message, the hospital determined the cost and time to implement HL7 messaging was unacceptable for the immediate need. The hospital was interested in exploring other options of transferring results electronically.

SOLUTION:

In January 2017, alternatives to HL7 were discussed and the decision was made to initiate a pilot program to send newborn screening results in PDF Format via secure file transfer protocol (SFTP). The goals of this project included:

- Demonstrate that this task could be implemented quickly and at minimal cost.
- Setup an agency infrastructure to send files in PDF or HL7 format.
- Demonstrate to hospital staff that this process would be faster and more efficient for receiving results and require less staff time in managing reports.

The PerkinElmer LIMS system used by the Newborn Screening Program already generated a PDF of all newborn screening results and batched them by fax number. A routine was written to pull all PDF assigned to a fax number associated with the hospital into a designated folder at ODH. A naming convention for the PDFs was agreed upon that contained identifying information that could be used to assign reports to hospital medical record. A secure SFTP connection was set up between ODH and the pediatric hospital.

The first batch of reports was transferred the hospital via SFTP the first week of March 2017. In order to validate that all records were included in the PDF folder, the hospital has continued to receive every result by both PDF and fax to reconcile any differences. Transmission reports were also developed to specify the number and IDs of PDFs included in the file transfer. Recently, two auditing routines were created to 1) assure that each report is sent and 2) assure that each report is received. This should eliminate the need for manual auditing.

This pilot project showed that the infrastructure for sending electronic results could be set up quickly and at little cost. This process could easily be expanded to send electronic results to multiple hospitals. Currently the data is being sent in PDF format, but the same infrastructure could be used to send HL7 messages. Because of the need for a bidirectional SFTP line, it is unlikely that this would be the method of electronic result transmission for primary care physicians in the community.

VENDOR CASE STUDY #1

by OZ Systems

ADT Message Validation with a Hospital Partner

BACKGROUND:

OZ Systems (OZ) partnered with the Virginia Department of Health (VDH) – Division of Consolidated Laboratory Services (DCLS) to improve newborn screening timeliness by implementing Telepathy™ Newborn Screening (NBS) at Virginia hospitals. A select group of hospitals purchased the Newborn Admission Notification Information (NANI) tool to automate the near to real time transmission of demographic data from hospital electronic health records (EHRs) to the Telepathy™ NBS application. NANI provides a baseline denominator of all hospital births, and reduces the amount of data entry on the newborn screening card. NANI is an Integrated the Healthcare Enterprise (IHE) profile that is based on Health Level 7 (HL7) Version 2 Admission, Discharge, and Transfer (ADT) messages.

PROBLEM:

Newborn screening cards are often missing data elements or are illegible. Handwriting discrepancies can lead to misspelling upon data entry and a longer time to follow-up on children if the data are illegible. While every baby in a hospital has an electronic health record, their data are not always recorded on the newborn screening card. The lab often spends time tracking down the correct information from the hospital due to missing data elements or illegibility.

SOLUTION:

The OZ solution, Telepathy™ NBS eliminates handwriting on the card, by populating data elements from the EHR, and sends electronic order messages to the Laboratory Information System (LIMS). To begin this process, OZ reached out to hospitals who were interested in the pilot project and contracted with them to implement NANI as part of the Telepathy™ NBS solution. While ADT messages are commonly used in hospitals, NANI requires message validation to identify minor modifications and ensure that the information is transmitted in the standard format. Initially OZ worked with DCLS to identify the required data elements for the program and those data elements that were required but could be left empty.

At each facility, OZ started with a kick-off to review the overall project and identify a project team. The facility received documentation outlining technical specifications and requirements. The hospital modified the facility's ADT messages to be compliant with the documentation.

OZ worked closely with facilities to identify the required data elements. Content testing occurred and issues were identified and resolved. The hospital had the ability to validate its own messages for compliance with the NANI documentation by using a proprietary online NANI validation tool. This allowed the hospital to have control over initial validation to determine changes. The OZ team further validated the messages to ensure they populate appropriately into the Telepathy™ NBS application. OZ Systems used free tools to validate content and connectivity such as SmartHL7 Viewer and SmartHL7 Sender (http://smarthl7.com/).

VENDOR CASE STUDY #1

by OZ Systems

ADT Message Validation with a Hospital Partner (continued)

SOLUTION (continued):

There were three control messages developed, an A01, an A03 and an A08. These messages are tested throughout the process. The team created test scenarios to ensure all data elements are appropriately validated including, but not limited to a healthy baby who was admitted, updated and discharged within the well-baby nursery; a sick baby who is admitted, moved the a higher level of care and discharged home; and a deceased baby who is born but passes away prior to discharge from the facility. Within these three scenarios robust demographic data elements are captured for patient and next of kin based on the requirements specifically identified by DCLS.

The most common findings through message validation were:

- Data elements such as relationship type, race and ethnicity required translation tables to ensure the use of HL7 standard codes. For example, relationship type should be sent as the code "MTH" to indicate Mother, however, some hospitals used M, Mother and Mom.
- Birth Weight required a conversion from kilograms to grams.
- Time of Birth required special testing and algorithms in cases where EHRs did not send time in their date/time of birth field.

Lessons learned showed that when developing timelines, the team needs to consider competing EHR related projects and the availability of facility staff, such as network engineers for VPNs and ADT testers. The understanding and expertise of hospital staff influence the speed of a NANI implementation.

oz systems

VENDOR CASE STUDY #2

by PerkinElmer, Inc.

Successes and Challenges of HL7 Data Exchange

BACKGROUND:

Newborn screening generates vast amounts of data that require continuous tracking. With effective information management, laboratories can increase process throughput and reduce costs. In response to this need, PerkinElmer, Inc. developed the world's first laboratory information management system specifically focused on the needs of NBS programs. Specimen Gate® software is designed to simplify the data collection and workflow processes involved with receiving specimens, screening for abnormalities, managing patient information, generating specimen reports, and following up abnormal and unsatisfactory specimens.

Over the years, PerkinElmer has successfully implemented 10 electronic messaging data flows with NBS laboratories across North America. Based on these experiences, PerkinElmer has prepared a high-level summary of the successes and challenges that these implementations entail.

CHALLENGES:

- NBS LIMS vendors may not have access to the end user result portal to view data sent from LIMS system. This access is important for end-to-end testing and validation.
- The National Library of Medicine Standard for Electronic Newborn Screening Result messages are known to the NBS community, but many outside organizations such as hospital IT vendors and Health Information Exchanges lack an understanding of and familiarity with this format.
- The NBS laboratory may not specify exactly what result data should be included in the electronic result message. By default, the NLM Standard is compiled to include all analyte, ratio, and disorder information, both quantitative and qualitative. After building the result message, the NBS laboratory may decide to only include data that appears on the printed patient report, resulting in customizations and additional validation.
- The NBS laboratory project manager does not prepare agendas, meeting minutes, or action items to maximize the efficiency of project meetings.
- Changes in project scope, such as committing to implement the NLM standard, then later deciding the standard does not meet NBS laboratory needs.
- Electronic result delivery to each hospital is difficult to configure and maintain by the NBS laboratory IT staff. A potential solution would be to only send results to a HIE, if available, and allow this entity to distribute electronic results to State hospitals and physicians.
- Custom message format that does not follow the NLM standard leads to unique configuration that may be more difficult to maintain and support long-term.
- The NBS laboratory does not have well-defined requirements on HL7 workflow and what data elements should and should not be included in the electronic message.
- The NBS LIMS vendor has limited access to development tools, restricted security access for development of the interface, or limited access to debugging tools for testing electronic messages.
- The NBS LIMS vendor does not have permissions to install the integration engine that is used to create and send electronic messages.

VENDOR CASE STUDY #2

by PerkinElmer, Inc.

Successes and Challenges of HL7 Data Exchange (continued)

Solution:

- Selecting a pilot hospital or small group of hospitals to receive the electronic result message helps to control scope and manage stakeholders.
- Order and result information workflows, connectivity and criteria are well-defined by the NBS laboratory.
- The NBS laboratory creates and executes a thorough test plan to validate all workflow and data exchange scenarios.
- The NBS laboratory project manager and IT staff are engaged and responsive throughout the project timeline.
- Utilization of an integration engine rather than a custom solution for electronic information exchange.
- Ability to have professional guidance and review from a nationally accepted and respected organization in the NBS community so that ALL electronic messages and workflows related to NBS adhere to a common standard.

In summary, active and engaged participation from NBS laboratory management and IT personnel are essential for a successful electronic data exchange project. Understanding data workflow and defining clear requirements at the beginning of the project are critical to keep the electronic message implementation on schedule. Changes to project scope and lack of support from the NBS laboratory or IT and partnering hospitals will lead to additional work efforts and extended timelines for all parties involved.

PerkinElmer
