# Supplementary material for: Newborn screening timeliness quality improvement initiative: Impact of national recommendations and data repository
Source: PLoS One. 2020 Apr 2;15(4):e0231050. doi: 10.1371/journal.pone.0231050 (PMC7117765; doi:10.1371/journal.pone.0231050)
Supplement: Attachment — Submitted filename: Response to Reviewers Sontag January 2 2020.docx [file pone.0231050.s004.docx]

Jacobus P. van Wouwe, MD PhD
Academic Editor
PLOS ONE

January 2, 2020

Dear Dr. van Wouwe and PLOS One Reviewers,

Thank you for your review of our manuscript, “Newborn screening timeliness quality improvement initiative: Impact of national recommendations and data repository.” We found the reviews very helpful, and we believe our manuscript is much stronger because of the suggestions provided. We have addressed the academic editor comments below, and the reviewers’ comments on the following pages.

Specific queries from the Academic Editor:

- Data Sharing: Data cannot be shared publicly because of the signed memoranda of understanding between newborn screening programs and the Association of Public Health Laboratories. Data requests are reviewed by the NewSTEPs Data Review Workgroup (contact via [*careema.yusuf@aphl.org*](mailto:careema.yusuf@aphl.org)) for individuals who meet the criteria for access to state-level data.
- Funding Sources and Financial Disclosure:
  - We have updated the financial disclosure statement to include additional funding from HRSA and the Cystic Fibrosis Foundation that was inadvertently omitted in the first draft. The edits are as follows:
    - **Funding Source:** The project described in this article was funded by the Department of Health and Human Services, Health Resources and Services Administration under Cooperative Agreements #UG8MC28554, #U22MC24078, and the Cystic Fibrosis Foundation under Grant Number SONTAG16Q10. HRSA provided support in the form of salaries for authors [MKS, JIM, SM, CY, RS, SE, SS, JO], but did not have any additional role in the study design, or data collection and analysis. The funder did participate in the decision to publish and in the preparation of the manuscript. The specific roles of these authors are articulated in the ‘author contributions’ section. The CFF did not have any role in the study design, or data collection and analysis, the decision to publish, or preparation of the manuscript.
  - Employment Status: We have edited the financial disclosure to indicate the employment status of all of the authors, and we have added the statement: "This does not alter our adherence to PLOS ONE policies on sharing data and materials.”
- Human Subjects Research: The NewSTEPs project was reviewed by the Colorado Multiple Institutional Review Board and deemed to be public health quality improvement and NOT human subject research. We have added a sentence to the methods section of the manuscript as well as the online submission system to reflect this.

We have made some edits to the abstract and some other minor edits to the body of the text to improve the flow and readability. All changes have been tracked, and no changes impact the overall conclusions and content of the report.

Thank you again for your review of this manuscript. Please contact us with additional questions.

Sincerely,

Marci Sontag, PhD

Director, Center for Public Health Innovation, CI International

Adjunct Associate Professor, Department of Epidemiology

Colorado School of Public Health

**Specific Comments from Reviewer #1:**

Abstract:

- Lines 67-69: Consider starting the abstract with the goal of newborn screening “Early identification and treatment of affected infants prior to the onset of symptoms”. Then define the necessary and time critical steps to accomplish the goal (i.e.…specimen collection, transport, testing, and reporting). The words “newborn screening activities” make it seem optional or less significant. These are steps are critically important to accomplishing the mission.

*AND*

Lines 77-78: Give a bit more detail on the necessary steps (Ie…specimen collection, transport, testing, and reporting). This will help lead into the results summary in line 79-90.

***Response:*** *Thank you for this comment. We have adjusted the abstract to reflect these changes in both the background and methods section of the abstract.*

- Line 91: This is the first time you mention NewSTEPs 360. Please define NewSTEPs 360, perhaps in the background or the methods section of the abstract.

***Response:*** *We have added NewSTEPs 360 in both the background and methods section of the abstract.*

Introduction:

- Line 101: Did you want to mention that clinics also collect newborn screening specimens, in addition to hospitals and midwives?

***Response*:** *The reviewer is correct. While most of the focus of the newborn screening timeliness activities were on the first specimen, clinics do indeed collect specimens. We have modified the introduction to reflect this.*

- Line 104: Consider rewording to “medical personnel to facilitate additional testing to confirm or exclude a diagnosis and also initiate the required appropriate intervention.” I see facilitation of additional testing as being the primary goal (first step). Intervention (although absolutely important under certain conditions) may or may not be needed depending upon the results.

***Response:*** *The reviewer brings up an excellent point. We believe the initiation of an appropriate intervention to be the ultimate goal for* ***affected*** *infants, and many times a confirmatory diagnosis may come months after an intervention starts. The reviewer accurately notes that classification of infants into affected vs unaffected is critically important. We have rephrased this sentence as suggested, to indicate diagnosis should occur first.*

- Figure 1: I struggled with figure 1. Your figure legend talks about NewSTEPs 360 and then lists five categories of activities performed by states to improve timeliness, but your figure only shows the process of newborn screening. I would suggest removing this text from the legend OR adding details to the figure to describe the five categories.

***Response:*** *The categories of activities are displayed in the linked boxes under each step in the NBS process. We believe figure one more clearly describes the process and the supporting activities within the process that were implemented within NewSTEPs 360.*

- Line 171-177: Consider rewording the last paragraph a bit to reflect each pieces of your review. You “summarized” the quality improvement initiatives. You “evaluated” the timeliness data for initial specimen collection, delivery from the birthing center, etc, over a certain time period. You “correlated” or “analyzed” or “assessed” the impact of individual program activities to improve timeliness.

***Response:*** Thank you, we have made the edits as suggested by the reviewer.

Methods:

- Lines 193-203: Consider rewording. State first what is available to the public, followed by what is available to registered users and then finally what states have access to after providing the MOU. The paragraph is a bit confusing.

***Response:*** This paragraph has been modified to better describe the processes in place for data sharing and data access.

- Line 210-224 and table 3: Did you want to use the benchmark of two calendar days for the timing between “specimen collection to receipt at lab”? Or only one day in transit, as shown in table 3?

***Response:*** This is a great observation. To avoid additional complicated descriptions we have chosen to present one day transit within column C of Table 3. We could have also modified the birth to specimen collection window to be one day, rather than two days. Each of these benchmarks is a bit arbitrary. We wanted to derive a metric to allow NBS programs to assess their performance in laboratory processes, and we did so using the metrics already defined by the ACHDNC, resulting in two days of laboratory testing for time-critical and four days for non-time-critical.

- Line 210: You mentioned in line 210 that you have added two additional timeliness benchmarks, what are the two benchmarks (2 days in transit and time to reporting?), but one is a modification to a metric and the other is a new metric? Please consider rewording the paragraph to provide clarity.

***Response:*** *This is an excellent point. We edited the paragraph to reflect that we modified one benchmark and added a new one.*

- Line 247: You refer to PDSA as a personality tool. I really thought it was a tool to assess a change that is implemented. The word personality confused me as well as how this tool could support team growth and team member’s roles.

***Response:*** *The PDSA tool that we have developed is a tool that does assess personalities, based on the quality improvement technique that you refer to. We have added a sentence that we believe clarifies the use of the tool.*

Results:

- Table 5: Define the “N” as the number of programs that provided data for a give category. Maybe in a footnote?

***Response:*** *Excellent point. We have added a note to the table legend to reflect the N is the number of programs that provided data for the quality indicator.*

- Table 5: Please clearly differentiate between reporting of results “presumptive positive for time critical disorders” “presumptive positive for non-time critical disorders”, and reporting of “all (normal and presumptive positive) screening results” OR reporting of “all normal results”?

***Response:*** *Thank you for noting this, we have made the changes as suggested.*

- Line 323: I just want to make sure the difference between each of the three categories (time critical/non-time critical/all) is clearly communicated. Plus…maybe this in inherently obvious…but I think it is necessary to state again…that the reporting of presumptive positive NBS results doesn’t mean that the patient is affected with the disease.

***Response:*** *The definitions of time-critical, non-time-critical are provided in the background of the document. We have added a sentence to the legend in Table 5 to define presumptive positive results.*

- Figure 2: Image is not clear/fuzzy. And it could also be larger. But maybe this will be addressed during the formatting process.

***Response:*** *Thank you, we will confirm that the figure is appropriately formatted upon final submission.*

- Line 344: “did not demonstrate change” OR “did demonstrate change”. It appears that the percentage when up slightly…but maybe this is not a significant change?

***Response:*** *The reviewer is correct, the program median for reporting was not significantly different (88.9 to 89.5% across the 3 years).*

- Table 7: This table is a bit confusing. For the six laboratories open 7 days a week, it is correct to read that 3 laboratories accession specimens all seven days and 3 laboratories accession specimens ONLY on Saturday? For the laboratory only open Monday through Friday, why do they have activities such as receiving specimens on Saturday? I believe I know what you mean to convey…but it is not inherently clear.

***Response:*** *Thank you for this comment. We have revised the table to make is less confusing. Specifically:*

- *We added M-F to the columns that indicate that testing occurs on Saturday or Sunday (M-F and Saturday, M-F and Sunday).*
- *We revised the table to reflect that the number of days a laboratory is open is defined by the number of days the laboratory tests specimens.*

- Line 407-408: The last sentence regarding the overall median for all results reported measures, comparing external versus state lab, is hard to appreciate from figure 5. It appears that the median would be closer to 80% for external labs. Maybe I would remove this sentence or clarify.

***Response:*** This sentence was removed as suggested.

Discussion:

- General comment: I really appreciate how you linked specific programmatic activities to the potential improvement seen within certain metrics.

***Response:*** *Thank you!*

- Line 521: Include within the limitations section of the discussion how second tier (or even third tier) testing (molecular or biochem) performed by laboratories may impact the time to reporting, even for critical conditions.

***Response:*** Thank you for this comment. This is an important word of caution for newborn screening programs, we have added it to the limitation section.

**Specific Comments from Reviewer #2:**

As suggestions I would like to put forward that I think the manuscript might be in use of extra context. The relevance of the topic is undisputed, but if the benchmark and the international context is presented more clearly, the reader would be able to grasp the relevance better. For example:

- How many cases are missed due to lack of timeliness? Which is hard to make concrete, because it would also require post-mortem genetic testing?

***Response:*** There have been some reports of tragic cases in which an infant has died or had poor outcomes due to a delay in newborn screening reporting, however it is hard to really assess this in a systematic way.

- What was the starting point of timeliness; there is mentioning of increasing time-critical disorders, but is this due to the type of disorders added to the RUSP or the revision of what a time-critical disorder is?

***Response:*** We added more details to the section regarding the expansion of the panel of NBS disorders (in the Introduction, Section on Timeliness in Newborn Screening) to reflect that the change really occurred with the introduction of MS/MS)

- How is this embedded or does it compare to international systems? Not all countries have birthing facilities / different facilities / deliveries at home, and the time of collection also differs quite a lot between countries.

***Response:*** This manuscript is definitely focused on the US systems. Many other countries do not screen for disorders using MS/MS and therefore they do not have the same urgency for early detection. Further, as the reviewer points out, other countries may have more babies born outside of traditional birthing facilities, and may also have newborn screen specimens collected at different times, coinciding with a home-visit from a nurse. These differences are beyond the scope of this manuscript.

- Was an implementation theory or model used? How did all parts fit into that?

***Response:*** No implementation theory or model was used but state activities addressed structural characteristics and the NewSTEPs 360 team provided ongoing implementation support activities (e.g. coaching) and building CQI self-efficacy for the NBS team

- Why was the selection based on a competitive application process?

***Response:*** Participation was based on a competitive process because each program had to demonstrate that they were prepared to make changes in their program and collect data to monitor progress.

- How is the quality and standardization of data in the repository ensured?

***Response:*** The data entered into the NewSTEPs repository are checked via data validation on each field upon data entry, and data inspection by the NewSTEPs staff, and NBS programs are queried to clarify identified outliers or suspected errors.

- Furthermore, I think the manuscript could be quite a bit shorter on the results if the tables are made more legible. Especially tables 5 and 6 are hard to read, because as a reader you are not always sure what you are looking at. Especially combined with the figures and the text, it seems there is a lot of double information, but the sources (how many labs answered which questions, i.e. how was dealt with missing values) are not always clear while potential stratification and/or combination of the variables is not made. Perhaps some information is better to put into tables instead of figures?

***Response:*** We have edited tables 5 and 6 to be clearer. We believe the stratification of the data into the tables and figures is important as there are nuances in these data that are important in the interpretation of the results of the study.

- Taking the above into account, I think it is important and interesting work, but to be an international scientific publication it seems to be in need of a better handle on highlighting only or more of the really interesting results to the reader; what tables and figures are really relevant?, and better embedding in international scientific literature, unless it is more or less solely aimed at US readers, but still evidence-based implementation theories / model could be applied.

***Response:*** The ACHDNC guidelines are intended for U.S. newborn screening programs, and many of the processes that are described in the manuscript are unique to the U.S.; however, we believe that many lessons can be gleaned for international audiences. We have better described the tables and figures, and we believe that the figures are necessary for the reader to understand the nuances of NBS programs in the U.S.

**Specific Comments from Reviewer #3:**

- Line 180: Participants in NewSTEPs. Selecting states is not selecting others. Can you explain what states were not selected, why, and whether this could introduce selection bias? (e.g., at limitations of study).

***Response:*** We have added a sentence to the limitations section to indicate that the application to NewSTEPs 360 could introduce a selection bias.

- Line 407: overall median was observed. Median of what?

***Response:*** This sentence was deleted in response to comment from Reviewer 1.

- Line 491 -500.
  I would like to know whether at the site of the laboratory, these CQI reports were generated automatically, querying the databases of the individual laboratories.. This information is important as it superior to manual transfer of data. If the data is entered manually in the files to upload in the repository, is it curated or checked at the site of the repository? This also applies on ln 204-section Quality Indicator data.

***Response:*** The reviewer has identified a critical point in the sharing of electronic data. Unfortunately, there are no methods available to automatically query the databases of all of the individual laboratories. The NewSTEPs team has made progress within some states and some of the Laboratory Information Management Systems (LIMS) vendors have individual solutions, this is not available broadly. Data checks are also implemented for data uploaded using automated upload tools.

- The discussion is quite lengthy and contains repeats of what was already said in the reminder of the paper. Moreover, the discussion is used to describe developments within the programs (e.g. a lengthy description of the efforts made to aIso work on weekend days feel that the authors can be more concise and ( Lines 512-520This is true, but does not relate to the study results.) . I feel the Conclusions section is a double of what has been said before in the paper, especially the discussion.

***Response:*** *Thank you for noting this. We have shortened both the conclusion and discussion section in light of your suggestions and we believe it reads better in its more concise form.*
