# Supplementary material for: Newborn screening timeliness quality improvement initiative: Impact of national recommendations and data repository
Source: PLoS One. 2020 Apr 2;15(4):e0231050. doi: 10.1371/journal.pone.0231050 (PMC7117765; doi:10.1371/journal.pone.0231050)
Supplement: Attachment — Submitted filename: Newborn screening timeliness quality improvement initiative_version 2 comments.doc [file pone.0231050.s005.doc]

- Line 102
  - Consider rewording to “in the first week of life”, instead of “first days or weeks of life”. Current recommendations target identification within the first week of life.
- Line 113 (figure 1)
  - Consider adding numbers to the figure to link the legend. For example, labeling the box within the figure “QI Solution 1”, which would link to the “1)” in the legend.
- Line 157
  - Consider rewording to “…barriers to timely NBS through technical and financial assistance”. I don’t understand what you mean by “utilization of technology”, unless you are referring to the visualization of performance metrics, quality indicators?
- Line 210
  - State that role-based access control was determined/granted at the individual NBS program level.
- Line 220-224
  - Wording is a bit unclear and could be made more concise
- Line 268 (table 4)
  - I “want” to link the various strategies (education, courier, NBS laboratory, etc) to the QI solutions listed in figure 1. It is possible to reconfigure this table to assign each strategy to the five QI solutions, highlighted in table 1.
- Line 310 (table 5)
  - I think the year should be “2016” instead of “2015”
- Line 313 (figure 2)
  - I am not sure if showing the lines on the graph for each individual state (connecting between the years and box plots) adds additional value. It may be more “clean” to only show the box and whisker plots. But this is just a preference…
- Line 377-379
  - These couple of sentences were confusing. The cases with confirmed diagnosis were identified between 2016-2018, so please consider including 2018 data to lines 377 to 379. Is there a different way to visualize the data, rather than text? Another table perhaps? Or maybe just consider removing, as you have the summary information above in table 6.
- Line 394
  - Spelling error “operations”
- Line 394 (table 7)
  - I am still struggling with table 7. For example, it doesn’t make sense that a state would report testing 7 days a week…but only receiving specimen on six days (M-F and Saturday) OR only reporting non-time critical results on Monday through Friday.
  - Because it is difficult to make sense of the table and because it is somewhat arbitrary (based upon reported practices of a given state), I think I would remove the table.
  - The text can remain as is…except maybe to highlight that each state “reported whether they were open 5, 6, or 7 days” and that “activities performed on a given day, vary between states”.
  - If you wanted to keep the table, please add additional sentences to the results or discussion that suggest how various activities performed 5, 6, or 7 days a week may have impacted the timeliness.
- Line 405
  - What do you mean by “laboratories open seven days demonstrated better outcomes than those open six or five days”? What is the “outcome” by which you used to evaluate whether a state performed better.
- Line 420-426 and 430-437
  - I don’t think it is necessary to include this information on whether it was a state laboratory or an external laboratory, unless you are trying to make the argument that “people might perceive that one is better than another…but in reality, both state labs and private labs can achieve the same success”? Again, just trying to understand the value of including this section.
- Line 451-460
  - Again, can we link these six different approaches listed to the 5 different QI solutions? Also see Line 268 (table 4) comment above.
- Line 463
  - Spacing error in text
- Line 520
  - Instead of the words “consult parents”, maybe consider using “educate parents”.
- Lines 522-528
  - Wording is a bit unclear and could be made more concise
