# Supplementary material for: Newborn screening timeliness quality improvement initiative: Impact of national recommendations and data repository
Source: PLoS One. 2020 Apr 2;15(4):e0231050. doi: 10.1371/journal.pone.0231050 (PMC7117765; doi:10.1371/journal.pone.0231050)
Supplement: Attachment — Submitted filename: Response to Reviewers Sontag March 2020.docx [file pone.0231050.s006.docx]

Jacobus P. van Wouwe, MD PhD
Academic Editor
PLOS ONE

March 10, 2020

Dear Dr. van Wouwe and PLOS One Reviewers,

Thank you for your comments on our revised version of our manuscript, “Newborn screening timeliness quality improvement initiative: Impact of national recommendations and data repository.” We found the reviews very helpful, and we believe our manuscript is much stronger because of the suggestions provided. We have addressed the academic editor comments below, and the reviewers’ comments on the following pages.

Thank you again for your review of this manuscript. Please contact us with additional questions.

Sincerely,

Marci Sontag, PhD

Director, Center for Public Health Innovation, CI International

Adjunct Associate Professor, Department of Epidemiology

Colorado School of Public Health

- Line 102
  - Consider rewording to “in the first week of life”, instead of “first days or weeks of life”. Current recommendations target identification within the first week of life.

**RESPONSE: Changed as suggested**

- Line 113 (figure 1)
  - Consider adding numbers to the figure to link the legend. For example, labeling the box within the figure “QI Solution 1”, which would link to the “1)” in the legend.

**RESPONSE: We have made the change to number to QI solutions as suggested, here and throughout the manuscript. Thank you for this suggestion.**

- Line 157
  - Consider rewording to “…barriers to timely NBS through technical and financial assistance”. I don’t understand what you mean by “utilization of technology”, unless you are referring to the visualization of performance metrics, quality indicators?

**RESPONSE: Changed as suggested**

- Line 210
  - State that role-based access control was determined/granted at the individual NBS program level.

**RESPONSE: Changed as suggested**

- Line 220-224
  - Wording is a bit unclear and could be made more concise

**Response: Changed as reflected here: Benchmarks were adapted directly from the ACHDNC recommendations. Few newborn screening programs were able to meet the ambitious 24-hours from specimen collection to receipt by laboratory benchmark set by the ACHDNC. In response to this, NewSTEPs created an additional benchmark of two calendar days to assess time elapsed from specimen collection to receipt at the laboratory as an intermediary step.**

- Line 268 (table 4)
  - I “want” to link the various strategies (education, courier, NBS laboratory, etc) to the QI solutions listed in figure 1. It is possible to reconfigure this table to assign each strategy to the five QI solutions, highlighted in table 1.

**Response This is a great suggestion. We have modified Table 4 to reflect the numbered QI solutions in Figure 1.**

- Line 310 (table 5)
  - I think the year should be “2016” instead of “2015”

**Response: You are correct. Thank you for catching this.**

- Line 313 (figure 2)
  - I am not sure if showing the lines on the graph for each individual state (connecting between the years and box plots) adds additional value. It may be more “clean” to only show the box and whisker plots. But this is just a preference…

**Response: We have left this figure in its initial format as we believe the changes in individual programs is important in quality improvement projects such as this one.**

- Line 377-379
  - These couple of sentences were confusing. The cases with confirmed diagnosis were identified between 2016-2018, so please consider including 2018 data to lines 377 to 379. Is there a different way to visualize the data, rather than text? Another table perhaps? Or maybe just consider removing, as you have the summary information above in table 6.

**Response: We have removed these sentences as suggested. They do not significantly add to the content.**

- Line 394
  - Spelling error “operations”

**RESPONSE: Changed as suggested**

- Line 394 (table 7)
  - I am still struggling with table 7. For example, it doesn’t make sense that a state would report testing 7 days a week…but only receiving specimen on six days (M-F and Saturday) OR only reporting non-time critical results on Monday through Friday.
  - Because it is difficult to make sense of the table and because it is somewhat arbitrary (based upon reported practices of a given state), I think I would remove the table.
  - The text can remain as is…except maybe to highlight that each state “reported whether they were open 5, 6, or 7 days” and that “activities performed on a given day, vary between states”.
  - If you wanted to keep the table, please add additional sentences to the results or discussion that suggest how various activities performed 5, 6, or 7 days a week may have impacted the timeliness.

**RESPONSE: We believe this table is an important table in the context of this manuscript. We have added text in the results section to describe some of the nuances of individual programs, and we have made additional edits as suggested by the reviewer to highlight that the laboratory hours are reported by an individual patient and that daily activities vary between states.**

- Line 405
  - What do you mean by “laboratories open seven days demonstrated better outcomes than those open six or five days”? What is the “outcome” by which you used to evaluate whether a state performed better.

**RESPONSE: The outcome highlighted here is result reporting. We have changed the text to remove the ambiguity. Thank you for identifying this.**

- Line 420-426 and 430-437 - I don’t think it is necessary to include this information on whether it was a state laboratory or an external laboratory, unless you are trying to make the argument that “people might perceive that one is better than another…but in reality, both state labs and private labs can achieve the same success”? Again, just trying to understand the value of including this section.

**Response: The reviewer brings up a good point. We have modified this section to indicate that both state and private labs can achieve good timeliness outcomes. We believe that this is important to note as there are a number of stakeholders that would like to suggest the privatization of NBS programs.**

- Line 451-460
  - Again, can we link these six different approaches listed to the 5 different QI solutions? Also see Line 268 (table 4) comment above.

**Response: We rearranged the numbering and modified the wording to be consistent with the approaches listed in Figure 1. We have not called it out directly, but it is now in parallel which we believe will be more satisfying for the astute reader.**

- Line 463
  - Spacing error in text

**RESPONSE: Changed as suggested**

- Line 520
  - Instead of the words “consult parents”, maybe consider using “educate parents”.

**RESPONSE: Changed as suggested**

- Lines 522-528
  - Wording is a bit unclear and could be made more concise

**RESPONSE: We modified the paragraph to be more concise.**
